# A Novel Benzocoumarin-Stilbene Hybrid as a DNA ligase I inhibitor with *in vitro* and *in vivo* anti-tumor activity in breast cancer models

**DOI:** 10.1038/s41598-017-10864-3

**Published:** 2017-09-06

**Authors:** Mohd. Kamil Hussain, Deependra Kumar Singh, Akhilesh Singh, Mohd. Asad, Mohd. Imran Ansari, Mohammad Shameem, Shagun Krishna, Guru R. Valicherla, Vishal Makadia, Sanjeev Meena, Amit Laxmikant Deshmukh, Jiaur R. Gayen, Mohammad Imran Siddiqi, Dipak Datta, Kanchan Hajela, Dibyendu Banerjee

**Affiliations:** 1 Medicinal and Process Chemistry Division, CSIR-Central Drug Research Institute (CSIR-CDRI), Lucknow, 226031 India; 2 Molecular and Structural Biology Division, CSIR-CDRI, Lucknow, 226031 India; 3 Biochemistry Division, CSIR-CDRI, Lucknow, 226031 India; 4 Academy of Scientific and Innovative Research (AcSIR), New Delhi, India; 5 Pharmacokinetics and Metabolism Division, CSIR-CDRI, Lucknow, 226031 India; 6 Department of Pharmaceutics, National Institute of Pharmaceutical Education and Research, Raibarelly, India; 7 Department of Chemistry Govt. Raza Post Graduate College, Rampur, 244901 India

## Abstract

Existing cancer therapies are often associated with drug resistance and toxicity, which results in poor prognosis and recurrence of cancer. This necessitates the identification and development of novel therapeutics against existing as well as novel cellular targets. In this study, a novel class of Benzocoumarin-Stilbene hybrid molecules were synthesized and evaluated for their antiproliferative activity against various cancer cell lines followed by *in vivo* antitumor activity in a mouse model of cancer. The most promising molecule among the series, i.e. compound (E)-4-(3,5-dimethoxystyryl)-2H-benzo[*h*]chromen-2-one **(19)** showed maximum antiproliferative activity in breast cancer cell lines (MDA-MB-231 and 4T1) and decreased the tumor size in the *in-vivo* 4T1 cell-induced orthotopic syngeneic mouse breast cancer model. The mechanistic studies of compound **19** by various biochemical, cell biology and biophysical approaches suggest that the compound binds to and inhibits the human DNA ligase I enzyme activity that might be the cause for significant reduction in tumor growth and may constitute a promising next-generation therapy against breast cancers.

## Introduction

Molecular hybridization is an important strategy in drug design and development based on the combination of pharmacophoric moieties of different bioactive molecules to produce a new hybrid compound with improved medicinal properties as compared to the parent molecules. The concept of hybrid molecules or molecular hybridization is now being increasingly used by pharmaceutical chemists in their quest for potent new drugs, as evidenced by the large number of recent reports on the synthesis of new bioactive hybrid molecules with the goal of creating new chemical entities that are medically more effective than their parent molecules^1–3^.

Combining the concept of molecular hybridization (MH) and biology-oriented synthesis (BIOS) we have synthesized a library of 30 stilbene (Resveratrol) and Neo-tanshinlactone based natural-product inspired hybrid molecules. The rationale behind the synthesis of natural product based hybrid molecules is the reduced general toxicity of the natural products because of their co-evolution with the targets and increased efficacy and selectivity. Out of 30 compounds one compound i.e. compound **19** showed better antiproliferative activity against the tested cancer cell lines as compared to their parent compounds (Neo-tanshinlactone and Resveratrol). The compound **19** is orally active in breast cancer syngeneic mouse model and shows good pharmacokinetic parameters to be considered as a lead molecule for future targeted therapy. Our *in-silico* studies showed that compound **19** may preferentially interact with human DNA ligase I (hLigI) protein, among various replication proteins that were tested. *In-silico* studies validated by *in-vitro* and *ex-vivo* biochemical assays showed that compound **19** specifically inhibits hLigI activity whereas FEN1 and PARP1 proteins were unaffected. Human DNA ligases are important proteins that maintain the genomic integrity of cells by joining the DNA strand breaks during DNA replication and repair processes^4–8^. An inhibition of hLigI activity leads to deficiency of DNA joining during DNA replication and repair process that leads to accumulation of DNA strand breaks leading to cell death^7, 9^. Since most adult cells in the human body do not replicate, or do so only sporadically (e.g., hematopoietic stem-cells), the highly proliferating cancer cells would be the primary target for hLig1 inhibitors while the side-effect on non-dividing normal cells is predicted to be minimal.

There are three major forms of human DNA ligases (human DNA ligase I, III and IV) that have been described in the literature^8, 10^. Among the three, hLigI is the major form in replicating cells. Elevated levels of DNA ligase I have been found in several cancer cells like breast, lung and ovarian cancer cells^11^. Several attempts have been made to search for specific inhibitors against DNA ligases since the year 2004 after the discovery of the crystal structure of hLigI^12^. Some ligase inhibitors exhibiting anticancer activity have been reported recently^13–20^, however none are presently used in therapy. Here, for the first time, we demonstrate an *in-vivo* active hLigI inhibitor which could be a lead molecule for the development of successful drug candidates.

## Results

### Chemistry results

**Figure 1:**
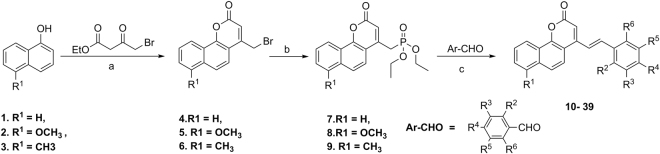
Synthesis of Benzocoumarin-Stilbene hybrid molecules 10–39 via Horner- Wadsworth- Emmons (HWE) reaction. Reagents and conditions**:** (**a**) H_2_SO_4_, RT, 3 h; (**b**) P(OEt)_3_, 120 °C, 2-3 h, () MeONa, DMF 0 °C −RT, 70 °C, 72–98%.

We designed and synthesized a library of *E*-benzocoumarin-stilbene hybrids *via* Horner-Wadsworth-Emmons (HWE) reaction (Fig. 1). Acid catalyzed condensation of 4-bromo ethylacetoacetate^21^ with 1-napthols (**1–3**)^22^ led to the formation of 4-bromomethyl-benzocoumarin derivatives **4–6** in quantitative yields. Subjecting the 4-bromomethylbenzocoumarin derivatives **4–6** to Michaelis-Arbusov reaction^23, 24^ with triethylphoshphite afforded the phosphonic acid diethyl esters **7–9** as crystalline compounds in 82–95% yield. Subsequently, HWE reaction^25^ of the phosphonic acid diethyl esters **7–9** with substituted aldehydes in dry DMF/NaOMe formed the pure compounds, (E)-4-styryl-2H-benzo[h]chromen-2-ones **10–39** in 72-96% yield (Table 1).

**Table 1:** Synthesis of benzocoumarin-stilbene hybrid system via HWE reaction.

Comp. No	R^1^	R^2^	R^3^	R^4^	R^5^	R^6^	Yield (%)
10	H	H	H	H	H	H	95
11	H	H	H	OCH_3_	H	H	80
12	H	H	H	N(CH_3_)_2_	H	H	75
13	H	H	H	NO_2_	H	H	96
14	H	H	H	CN	H	H	92
15	H	H	H	H	OCH_3_	H	85
16	H	OCH_3_	H	H	H	OCH_3_	90
17	H	H	OCH_3_	OCH_3_	H	H	82
18	H	H	OCH_3_	OCH_3_	OCH_3_	H	86
**19**	**H**	**H**	**OCH**_**3**_	**H**	**OCH**_**3**_	**H**	**95**
20	H	H	OCH_3_	H	H	OCH_3_	80
21	H	H	H	OCH_3_	H	OCH_3_	78
22	OCH_3_	H	H	H	H	H	92
23	OCH_3_	H	H	OCH_3_	H	H	80
24	OCH_3_	H	H	NO_2_	H	H	94
25	OCH_3_	H	H	CN	H	H	92
26	OCH_3_	H	OCH_3_	H	OCH_3_	H	84
27	OCH_3_	H	OCH_3_	OCH_3_	H	H	82
28	OCH_3_	OCH_3_	H	H	H	OCH_3_	78
29	OCH_3_	H	OCH_3_	H	H	OCH_3_	75
30	OCH_3_	H	H	OCH_3_	H	OCH_3_	72
31	OCH_3_	H	OCH_3_	OCH_3_	OCH_3_	H	82
32	CH_3_	H	H	H	H	H	95
33	CH_3_	H	H	NO_2_	H	H	95
34	CH_3_	H	OCH_3_	H	OCH_3_	H	95
35	CH_3_	H	H	OCH_3_	H	OCH_3_	80
36	CH_3_	H	OCH_3_	H	H	OCH_3_	75
37	CH_3_	OCH_3_	H	H	H	OCH_3_	72
38	CH_3_	H	OCH_3_	OCH_3_	OCH_3_	H	76
39	CH_3_	H	H	OCH_3_	OCH_3_	H	70

### Biology results

#### Effect of Benzocoumarin-Stilbene hybrids on viability of different cancer cell lines

**Figure 2:**
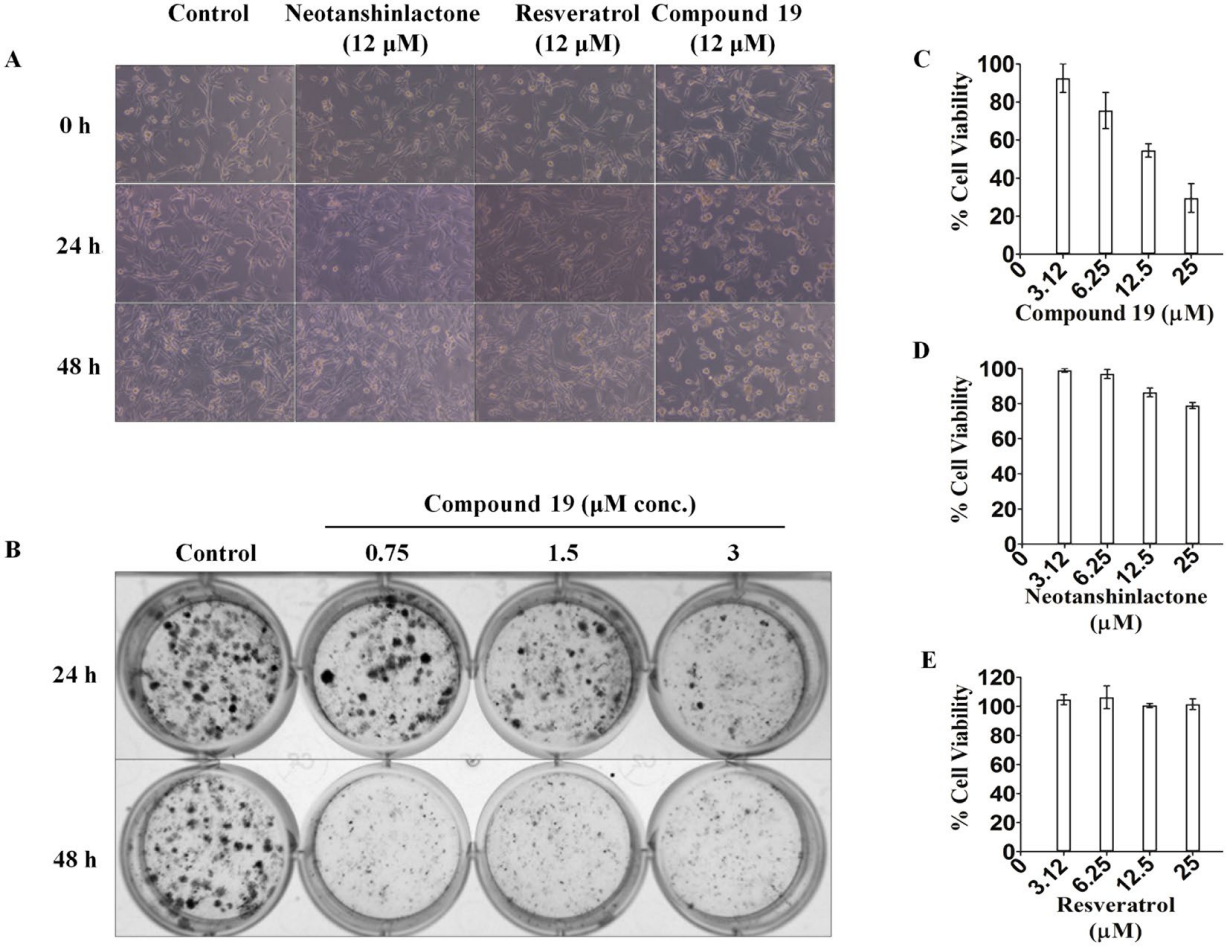
(**A**) Bright field images of MDA-MB-231 cells treated with vehicle control, Neo-tanshinlactone, Resveratrol and compound **19**. The cells treated with compound **19** showed changes in cellular morphology. (**B**) Images of colony forming assay showed growth inhibition of colonies after the treatment with different concentrations of compound **19**. Graphs C, D and E show the activities of Compound **19**, Neo-tanshinlactone and Resveratrol respectively in MDA-MB-231 cells.

The cytotoxicity of all 30 compounds (Benzocoumarin-stilbene hybrids) were evaluated in breast cancer cell lines (MDA-MB-231) (Supplementary Table 1). At 10 μM concentration, compounds 16, **19** and 26 showed significant activity ( > 50% inhibition) in MDA-MB-231 cells (Supplementary Table 1). The activity of these compounds were further checked in different cancer cell lines such as Colorectal adenocarcinoma (DLD-1), Liver hepatoma (PLC/PRF/5), Lung carcinoma (A549), Ovarian adenocarcinoma (SK-OV-3), Brain glioblastoma (A-172), Pancreatic adenocarcinoma (PANC-1), and triple negative mouse breast cancer cell line (4T1) (Supplementary Table 2). Of all the compounds tested, compound 19 showed maximal activity in different cancer cell lines including MDA-MB-231 and 4T1 with IC_50_ values 12.5 ± 2.8 and 11.6 ± 1.7 μM respectively. Although the compound showed activity against MCF10A (human mammary epithelial cell line), but due to the non-toxic and cytostatic nature of the compound, we decided to test the compound in our *in vivo* breast cancer mouse model developed using syngeneic 4T1 cells, against which the compound showed best activity (Fig. 2B).

We studied the compound **19** induced morphological changes in MDA-MB-231 cells. The bright field images of cells shown in Fig. 2A clearly indicate that the compound induces conspicuous morphological changes in cells after 24 and 48 h, as compared to its parent compounds (Neo-tanshinlactone and Resveratrol) or vehicle control, at its IC_50_ concentration (12 μM). Compound **19** inhibited colony forming ability of cells (Fig. 2B) and after 24 and 48 h of treatment (at 3, 1.5 and 0.75 μM concentrations) also reduced the size of the limited colonies that formed. This indicated that the compound may have a cytostatic activity against MDA-MB-231 cells at these concentrations. We performed comparative cell viability assays for compound **19** and its parents (Neo-tanshinlactone and resveratrol) and found that compound **19** was more active as compared to parent compounds (Fig. 2C–E).

### Compound 19 induced apoptosis in MDA-MB-231 cells and arrested cell cycle progression at S and G2/M phases

**Figure 3:**
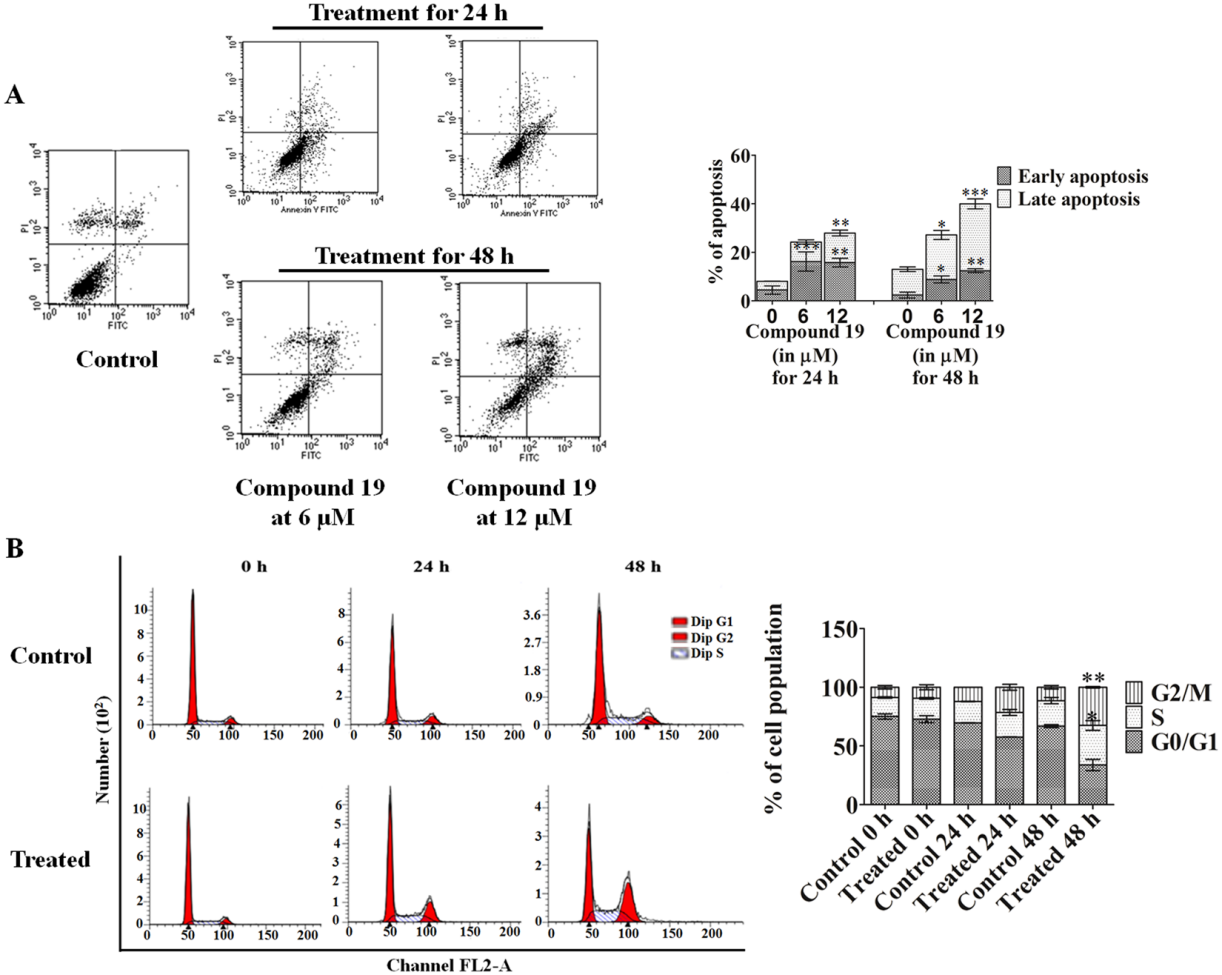
(**A**) Quantification of apoptosis induced by different concentrations of compound **19** in MDA-MB-231 cells at 24 and 48 h. The percentage population of early and late apoptotic cells at various concentrations of compound **19** are represented graphically. (**B**) The quantitative distribution of cells in different phases of the cell cycle is represented graphically. DNA histograms clearly show that compound **19** (at 12 µM for 0, 24 and 48 h) induced cell cycle arrest at the S and G2/M phase of cell cycle. The data shown are mean ± SEM of three independent experiments. Significant difference from control was observed by ANOVA: *P < 0.05, **P < 0.01, ***P < 0.001.

Quantitative analysis of the induction of apoptosis was performed in MDA-MB-231 cells using flow cytometry. MDA-MB-231 cells were treated with different concentrations (6 and 12 µM) of compound **19** for 24 and 48 h. Percentages of cells undergoing apoptosis increased with increasing concentrations of compound **19** and their treatment duration (Fig. 3A). At 24 h of treatment, 10.78 ± 1.21% and 16.37 ± 1.18% apoptosis was observed in MDA-MB-231 cells at 6 and 12 µM concentrations, whereas after 48 h of treatment a much higher 18.68 ± 4.86% and 36.17 ± 3.43% of cells were observed to undergo apoptosis at the same concentrations.

To study the effect of compound **19** on cell cycle progression, MDA-MB-231 cells were synchronized by serum starvation for 48 h. Thereafter, cells were released into serum containing media and treated with 12 µM of compound **19** for 0, 24 and 48 h. At 0 h, both treated and untreated cells were arrested at G0/G1 phase (75.12 ± 2.23%), with only 16.18 ± 0.65% cells in the S-phase and 8.71 ± 1.57% cells in the G2/M phase (Fig. 3B). After 24 h, in control samples, 69.63 ± 0.21% cells were still present in G0/G1 phase while 18.31 ± 0.13% cells in S phase and 12.06 ± 0.08% cells were in G2/M phase. However, in the treated sample a much lower 57.61 ± 0.1% cells were in the G0/G1 phase while 20.91 ± 2.61% and 21.48 ± 2.50% cells were present in S and G2/M phases of cell cycle respectively. Figure 3B, also shows similar results after 48 h of treatment with 66.87 ± 1.23% cells in G0/G1 phase, 21.8 ± 2.72% cells in S phase, and 11.32 ± 1.48% cells in G2/M phase in control cells whereas in treated cells a significantly lower 33.78 ± 4.77% cells were in G0/G1 phase, 33.7 ± 4.2% in S phase and 32.53 ± 0.58% cells were in G2/M phase, respectively. The graph in Fig. 3B, clearly shows that at different time points, compound **19** increased the population of cells in S and G2/M phase and decreased the cell population in G0/G1 phase, indicating that it blocks the cell cycle at S and G2/M phase.

### Compound 19 inhibits migration of MDA-MB-231 cells

**Figure 4:**
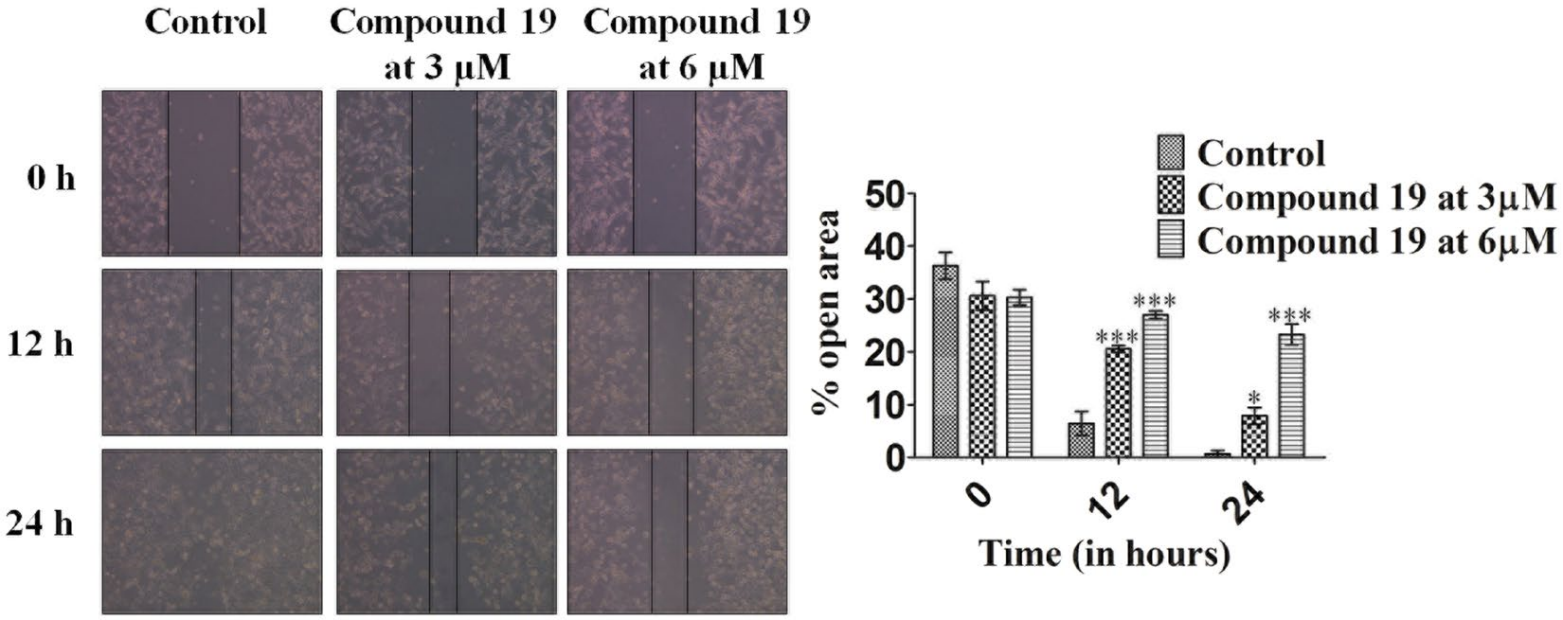
Bright field images of MDA-MB-231 cells treated with compound **19** demonstrate the inhibition of cellular migration. The graph on the right clearly shows the decrease in the percentage of open area in control cells versus treated cells at different time points. The data shown are mean ± SEM of three independent experiments. Significant difference from control was observed by ANOVA: *P < 0.05, **P < 0.01, ***P < 0.001.

Cancer cells possess the property of migration from their origin to distant sites which is called metastasis^26^. It is particularly dangerous for patients as this leads to the spread of cancer from the site of formation to other organs of the body and leads to flaring up of the condition and poor disease prognosis. We performed the scratch assay to check if the migration ability of MDA-MB-231 cells was altered in the presence of different concentrations (3 and 6 µM) of compound **19** (when treated for 12 and 24 h). We calculated the percent open area in control and treated cells at different time points with the help of TScratch software. The bright field images and graph in Fig. 4 show that the percent open area decreases more in control cells as compared to treated cells. The percent reduction in open area indicates the increase in migration ability of cells. In Fig. 4, we clearly demonstrate that compound **19** could significantly inhibit the cell migration at concentrations of 3 and 6 µM where no significant antiproliferative activity was observed.

### Compound 19 induced DNA damage and activated the intrinsic pathway of apoptosis in MDA-MB-231 cells

**Figure 5:**
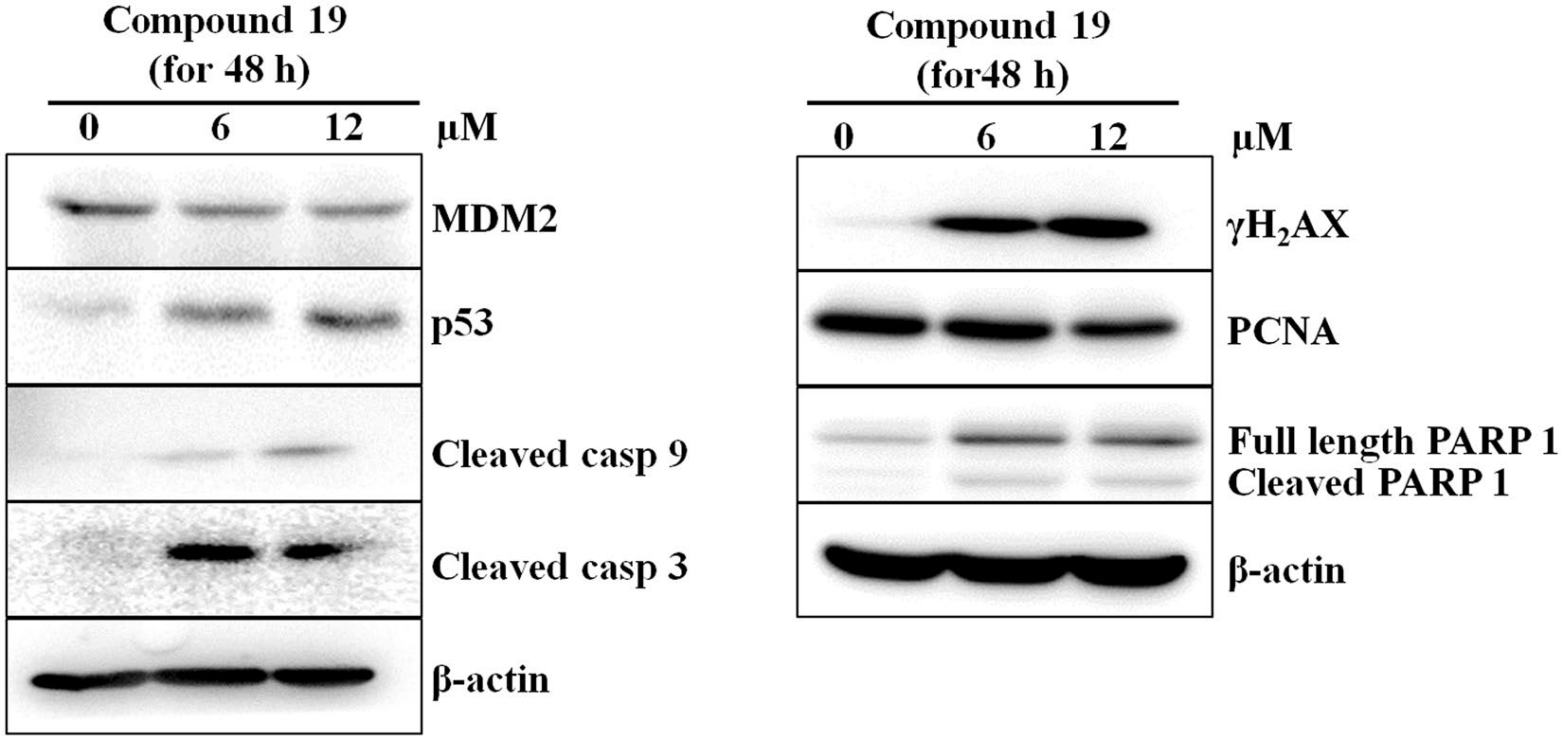
Western blotting images show differential expression of proteins after the treatment of compound **19** in MDA-MB-231 cells. The blot images are representative of three independent experiments.

We performed western blotting experiment to study the compound **19** induced apoptotic signaling events in MDA-MB-231 cells. As shown in Fig. 5, compound **19** increased DNA damage inside the cells as demonstrated by increased expression of γH_2_AX in cells^27^. We also performed comet assay to study the DNA damage caused by compound **19** and found a significant increase in the tail length of treated cells (increased tail length of DNA occurs due to DNA damage) as compared to control (Supplementary Fig. 71). Cells try to repair the damaged DNA by increasing the expression of PARP-1 protein; the level of PARP-1was found elevated cells treated with compound 19. However, during extensive DNA damage, PARP-1 is cleaved into 89 kDa and 24 kDa fragments by a caspase 3 mediated pathway that promotes the cells to undergo apoptosis instead of necrosis^28^. This was observed in our western blotting results in Fig. 5. Our western blotting results also showed an increase in expression of p53, decrease in expression of MDM2 (negative regulator of p^53^) and PCNA (cell proliferation marker). In addition, the compound promotes the cleavage of caspase 9 and caspase 3 in MDA-MB-231 cells. These results indicate that compound **19** promotes p53 mediated intrinsic pathway of apoptosis in MDA-MB-231 cells at the tested concentrations.

### Assessment of pharmacokinetic properties of the novel bioactive anticancer compound 19

**Figure 6:**
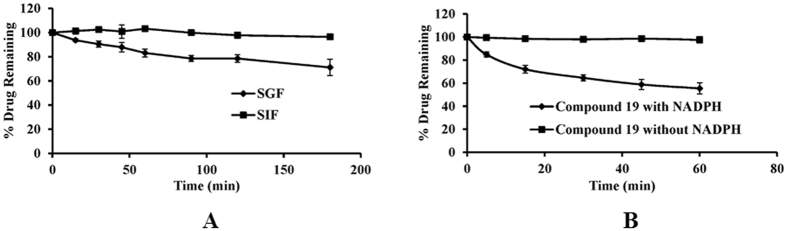
Stability studies of compound **19**. (**A**) Simulated gastric fluid (SGF) and simulated intestinal fluid (SIF) stability of compound **19** at 2 µg/mL concentration. (**B**) Metabolic stability of compound **19** at 5 µM concentration in rat liver microsomes (RLM). The data shown are mean ± SEM of three independent experiments.

The oral route of administration is a more preferred route than others, and the drugs must therefore be stable in SGF (simulated gastric fluid) and SIF (simulated intestinal fluid) respectively. Compound **19** showed 83.19 ± 3.22% and 96.45 ± 0.79% stability in SGF and SIF up to 180 min (Fig. 6A). Thus, compound **19** was suitable for oral administration as it was stable in SGF and SIF.

In microsomal metabolic stability study, compound **19** was found to be stable in control reaction in the absence of cofactor, confirming the chemical stability as well as cofactor dependent degradation. The compound was 55.5 ± 4.77% stable up to 60 min (Fig. 6B).

The pharmacokinetic parameters followed by the oral and I.V administration were calculated using non-compartmental model listed in Table 2. After oral administration (50 mg/kg) the compound exhibited a C_max_ of 13.38 ± 2.92 ng/mL at 0.5 hr (t_max_). Following I.V (5 mg/kg) dosing, the plasma concentration of compound **19** was found to be 325.30 ± 101.88 ng/mL at 5 min. *In-vivo* t_1/2_ of compound **19** was found to be 19.25 ± 11.75 h and 22.77 ± 4.69 h for I.V and oral PK study, respectively. AUC_0-t_ of compound **19** was found to be 457.15 ± 130.59 hr*ng/mL and 341.57 ± 18.64 hr*ng/mL after I.V and oral PK study, respectively. The percentage bioavailability of compound **19** was found to be 7.85 ± 0.65%. After oral dosing, compound **19** showed detectable plasma concentrations up to 72 h, and it could provide chronic onset of action at the detectable levels. This chronic onset of action could be an advantage in chronic clinical conditions like cancer.

**Table 2:** Pharmacokinetic parameters of compound 19 after intravenous and oral administration.

Pharmacokinetic parameters	I.V route	Oral route
Dose (mg/kg)	5	50
T_1/2_ (hr)	19.25 ± 11.75	22.77 ± 4.69
T_max_ (hr)	—	0.5
C_max_ (ng/mL)	325.30 ± 101.88	13.38 ± 2.92
AUC_0-t_ (hr*ng/mL)	457.15 ± 130.59	341.57 ± 18.64
Bioavailability (%F)	—	7.85 ± 0.65

### Compound 19 arrested breast tumor progression *in-vivo*

**Figure 7:**
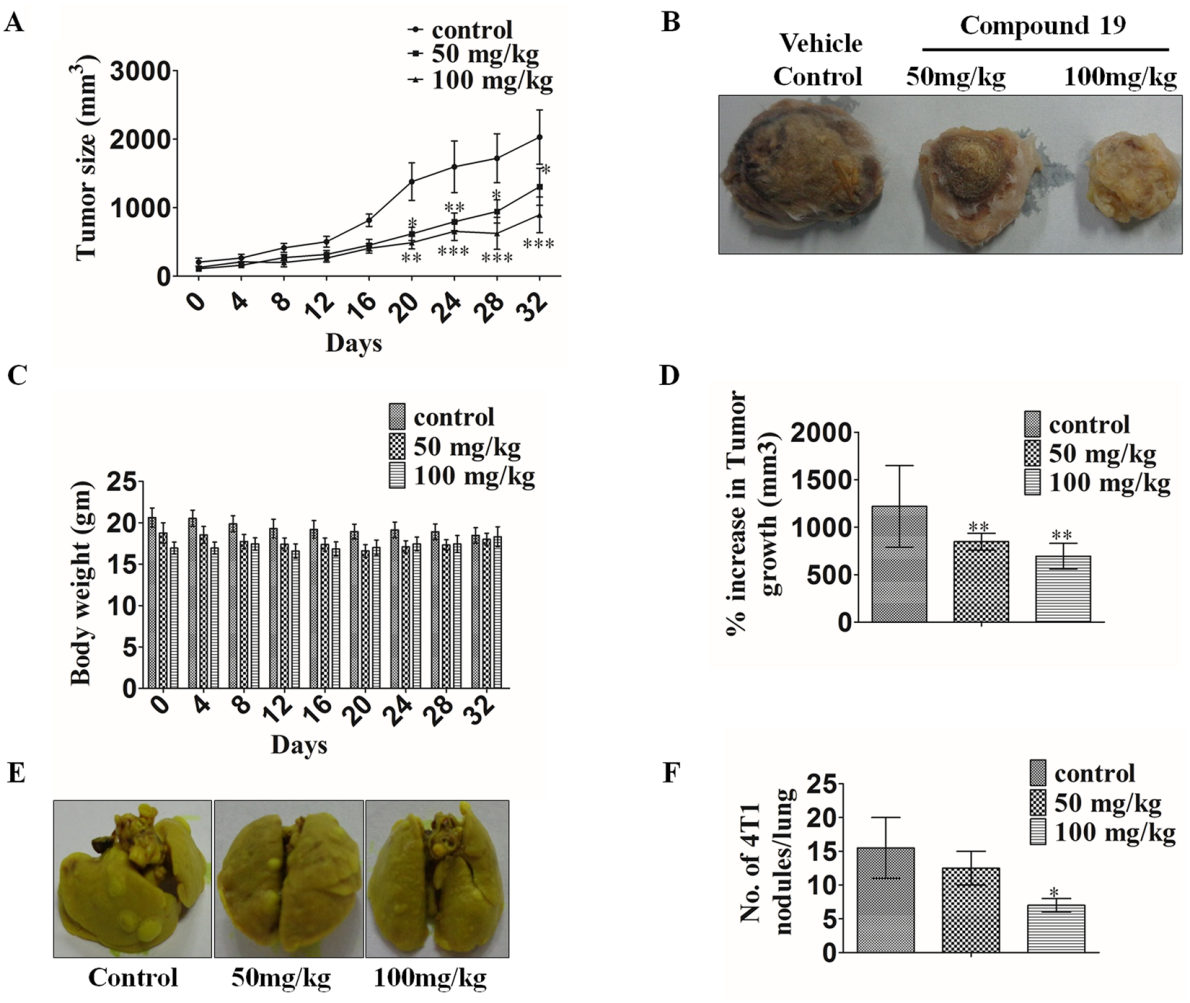
*In-vivo* antitumor activity of compound **19** in a syngeneic (4T1 induced) orthotopic mouse model of breast cancer. **(A)** Effect of the compound on breast tumor progression is represented graphically for every fourth day. **(B)** Representative images of control and treated tumors at 50 and 100 mg/kg body weight. **(C)** Effect of the compound on body weight of mice is shown graphically. **(D)** Graphical representation of % increase of tumor size in control and treated mice. **(E)** Representative image of the lungs of mice showing 4T1 nodules in control and treated mice. **(F)** Number of 4T1 nodules in each lung represented by a graph. The data shown are mean ± SEM of N = 6 mice of each group in two different experiments. Significant difference from control was observed by ANOVA: *P < 0.05, **P < 0.01, ***P < 0.001.

Having validated the *in-vitro* activity of compound **19**, we utilized the syngeneic 4T1 mouse orthotopic breast cancer model to evaluate the *in-vivo* antitumor efficacy at 50 and 100 mg/kg doses. The compound inhibited breast tumor growth *in-vivo* in a dose dependent manner as compared to vehicle treated animals when it was administered daily via oral gavage for 32 days (Fig. 7A,D). Pictures of harvested tumors (Fig. 7B) clearly demonstrated a marked reduction in tumor size in animals treated with the compound compared to vehicle control group. There was no significant change in the average body weight of control and treated animals for the duration of the experiment, suggesting the apparent non-toxic nature of the compound (Fig. 7C). A marked decrease in breast cancer metastasis to the lungs was also observed (Fig. 7E,F). A marked reduction in the number of 4T1 nodules on the lungs of treated mice can be seen as compared to vehicle control mice. This indicates that the compound inhibits the migration of breast cancer cells from their site of origin to the lungs as earlier indicated by our *in-vitro* scratch assay (Fig. 4). These experiments clearly demonstrate the antitumor and anti-metastatic activity of compound **19**.

### Possible mode of action of compound 19

Our cell based assays showed that compound **19** arrested the cell cycle progression of MDA-MB-231 cells at S and G2/M phases and promoted the accumulation of DNA damage as seen by an increase in the level of γH_2_AX (Figs 3,5 and Supplementary Fig. 71). DNA replication and repair processes occurs mainly at S and G2/M phase of cell cycle^29–32^ suggesting that compound **19** might be targeting one or more of the DNA replication and repair proteins as its cellular target/s. This prompted us to carry out *in-silico* docking studies for compound **19** with various DNA replication and repair-related proteins to predict the possible cellular target/s of this compound.

**Figure 8:**
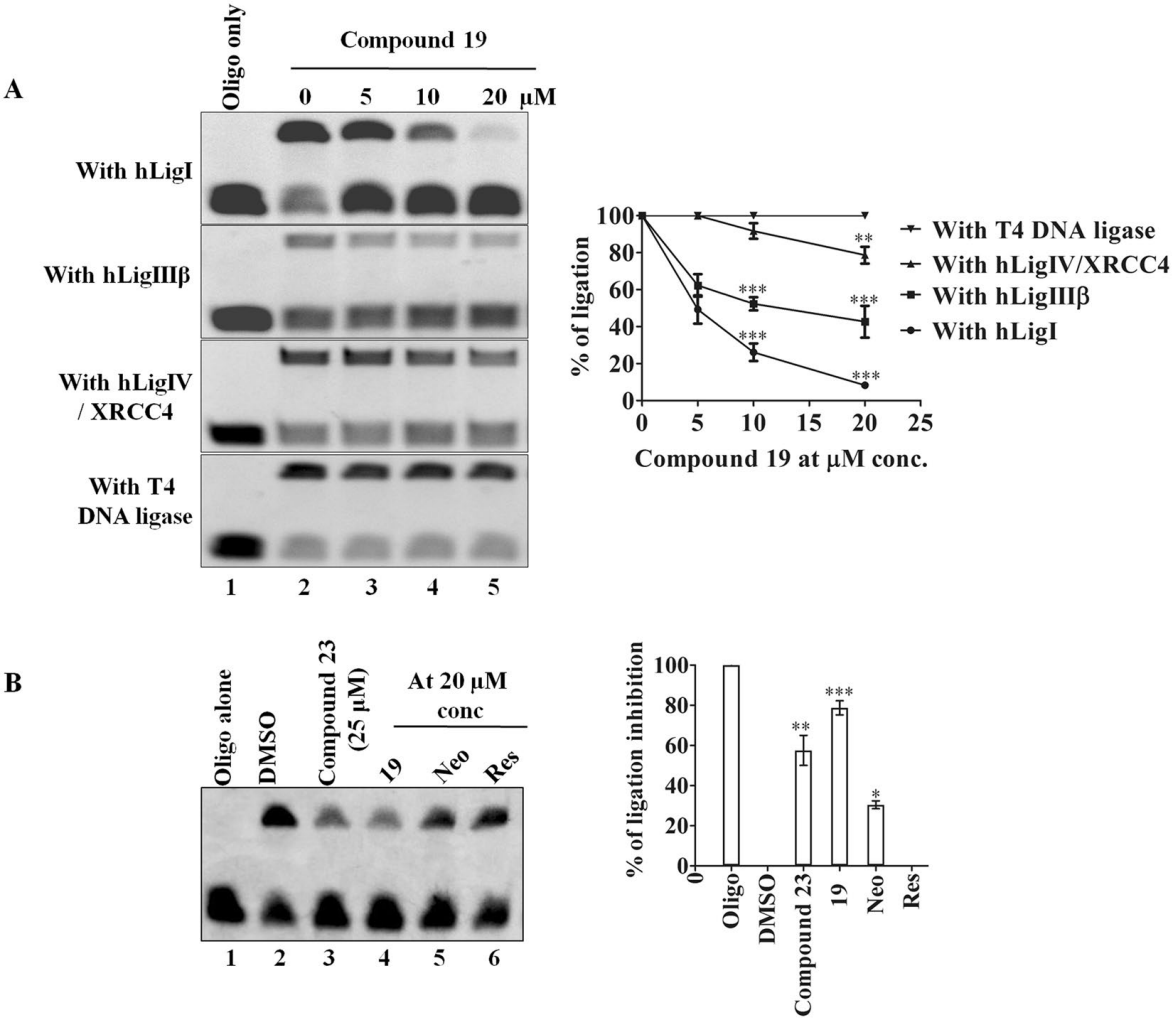
(**A**) Concentration dependent inhibition of DNA ligase activity by compound **19** at 0, 5, 10 and 20 µM concentrations against different human and non-human ligases. The gel picture and graph clearly showed specific hLigI inhibition activity of compound **19**. (**B**) A representative gel image and graph for inhibition of ligation by compound **19** and their parent molecules at 20 µM concentration. Compound **19** showed significantly better antiligase activity as compared to Neo-tanshinlactone (Neo) and Resveratrol (Res). Compound 23 was used as a positive control. The data shown are mean ± SEM of three independent experiments. Significant difference from control was observed by ANOVA: *P < 0.05, **P < 0.01, ***P < 0.001.

On the basis of docking scores (Supplementary Table 3) obtained from our *in-silico* studies we predicted hLigI (lowest docking energy) as a possible cellular target for compound **19**. We performed *in-vitro* ligation assay to confirm our *in-silico* prediction and found that compound **19** could inhibit hLigI activity. The ligation assays further proved compound **19** specifically inhibits the activity of hLigI (at 5, 10 and 20 µM concentrations), since the other human (hLigIIIβ and hLigIV/XRCC4) and non-human ligases (T4 DNA ligase) were unaffected by the compound at these concentrations (Fig. 8A). We also checked the functional inhibition of other proteins (PARP1 and FEN1) in the presence of compound **19** and found that the compound was not able to inhibit the function of FEN1 or PARP1 proteins even at 20 µM concentration (Supplementary Fig. 72 and 73). Compound **19**, which is hybrid of Neo-tanshinlactone and Resveratrol also showed better antiligase activity when compared to parent compounds Neo-tanshinlactone and Resveratrol. We used compound 23, a known antiligase compound reported in our previous study^19^ as a positive control for antiligase activity (Fig. 8B).

The above *in-vitro* results were supported by our *ex-vivo* assays where the cell lysate of MDA-MB-231 cells treated with different concentrations of compound **19** showed reduced capacity for DNA ligation (performed by hLigI) whereas flap cleavage activity (performed by FEN1) remained unaffected after treatment (Supplementary Fig. 74). These experiments indicate that compound **19** can specifically inhibit hLigI activity and could be its major cellular target.

### Compound 19 inhibits ligation by direct interaction with hLigI protein

**Figure 9:**
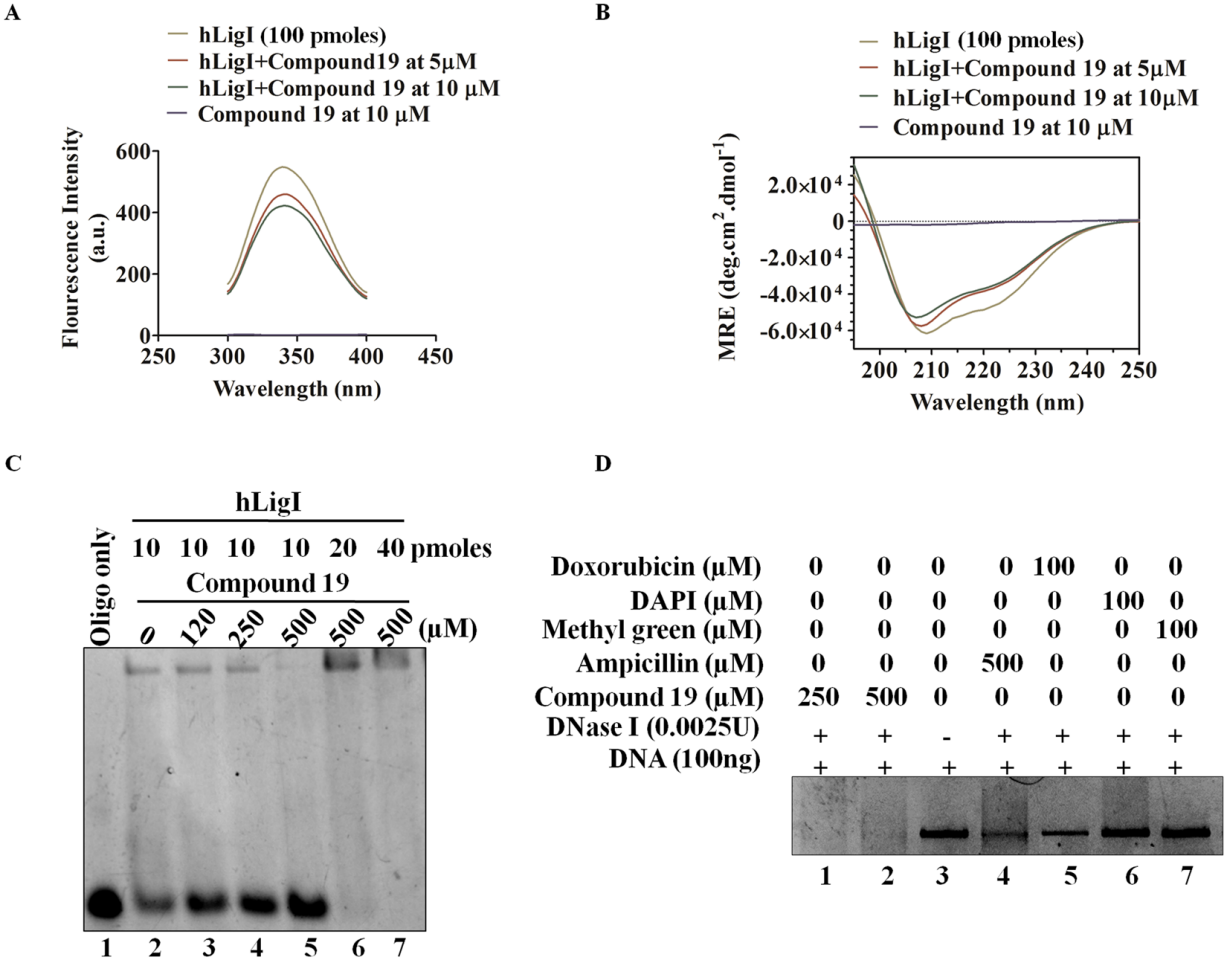
Interaction studies of compound **19** with hLigI and DNA. Fluorescence spectra of **(A)** hLigI in the presence of compound **19**, upon excitation at 280 nm. Fluorescence intensity is indicated in arbitrary units (A.U.). **(B)** CD spectroscopy shows conformational change in hLigI after addition of compound **19**. Spectra was recorded at the wavelength of 250-195 nm. **(C)** EMSA shows that compound **19** breaks the interaction between DNA and hLigI protein (lanes 3-5). For complementation, increasing concentrations of hLigI, when added back to the reaction, could rescue the hLigI-DNA complex even in the presence of the inhibitor (lanes 6-7). **(D)** DNase I cleavage assay rules out the possibility of any kind of interaction between compound **19** and DNA. The gel images are representative of three independent experiments.

Interestingly, a compound can inhibit ligation either by direct interaction with the protein where it blocks the DNA binding site (or another active or allosteric site) thereby blocking or changing its activity, or by binding with the substrate DNA, thereby occluding protein binding. In order to check both possibilities, we performed both protein and DNA interaction studies with compound **19** using various biophysical methods. Protein binding was checked by performing fluorescence quenching experiments followed by circular dichroism (CD) studies. In the presence of compound **19**, fluorescence spectra of the full length hLigI protein changed as compared to control samples, indicating protein binding (Fig. 9A). The Circular Dichroism (CD) studies also showed that the compound promoted conformational changes in protein (Fig. 9B) and decreased the hLigI and DNA complex formation (Lane 2 versus Lane 3-5, Fig. 9C). Moreover, the complex formation could be rescued by addition of a higher amount of hLigI (Lane 6 and 7, Fig. 9C). This again suggested protein binding. The key amino acid residues of hLigI involved in interaction with compound **19** are shown in Supplementary Fig. 75.

DNA binding of compound **19** was tested by the DNase I cleavage protection assay. The results from this experiment clearly suggested that unlike doxorubicin (a known DNA intercalator), methyl green (a known DNA major groove binder) or DAPI (a known DNA minor groove binder), the DNA incubated with compound **19** offered no protection from cleavage by DNase I up to 500 µM concentration (Fig. 9D, lanes 1-2 versus lanes 5-7). Thus, we may safely exclude any form of covalent interaction between compound **19** and DNA. Hence, from the above experiments, we may deduce that compound **19** exhibits its inhibitory activity through direct binding to hLigI and shows no interaction with the DNA substrate.

## Discussion

In this study, a novel class of compounds belonging to the Benzocoumarin-Stilbene hybrid family have been synthesized with the help of molecular hybridization approach. The most active compound of this series (compound 19) showed significantly better antiproliferative activity as compared to their parent compounds (Neo-tanshinlactone and Resveratrol) in MDA-MB-231 (hormone receptor negative) cells (Fig. 2). Our studies showed that compound 19 could arrest cell cycle progression at S and G2/M phases of cell cycle (Fig. 3), increase DNA damage, increase PARP cleavage, and finally promote a p53 mediated intrinsic pathway of apoptotic cell death in the TNBC cell line MDA-MB-231(Fig. 5).

The compound was also tested for its pharmacokinetic properties where it demonstrated stability in SGF and SIF and was found to be metabolically stable. The compound fulfills the Lipinski’s rule of five (Supplementary Table 4) which is necessary for a compound to be considered as a drug like compound^33^. All these are indications that compound **19** (or new compounds derived from it) may be developed into *in vivo* active drug like compounds (Table 2 and Fig. 6). Since compound **19** inhibits the proliferation of MDA-MB-231 cells and is suitable for oral dosing, we tested the *in-vivo* efficacy of compound **19** in mouse 4T1 cell-induced orthotopic breast tumor model by oral dosing (Fig. 7). Compound **19** arrested tumor progression but could not induce tumor regression indicating the somewhat cytostatic nature of compound **19** (Figs 2B and 7B). The compound also reduced metastasis of the highly metastatic 4T1 cells into the lungs *in-vivo* (Fig. 7E,F) which was observed by significantly less number of nodules in the lungs of animals treated with the compound. This was indicated earlier in our *in-vitro* scratch assay results, where MDA-MB-231 cells were observed to migrate significantly more slowly after treatment with compound **19**, as compared to control (vehicle treated) cells (Fig. 4). These results confirm the oral suitability, anti-breast cancer activity and anti-metastatic activities of compound **19**.

The mode of action and target specificity of compound **19** was studied by several methods. The observations of *in-vitro* studies (arrest of cell cycle progression and increase in DNA damage) prompted us to explore DNA replication and repair proteins as possible targets of this compound. *In-silico* docking studies revealed that compound **19** preferentially interacts with hLigI as compared to other DNA replication and repair proteins that were studied (Supplementary Table 3). The top hits from *in-silico* studies were validated by biochemical assays *in-vitro* and *ex-vivo* and revealed the highly specific ligase-inhibition activity of compound **19**. This activity of compound **19** was entirely different from that of the parent compounds which showed no ligase inhibitory activity (Fig. 8). The target specificity of compound **19** was validated by the functional activity against various proteins like hLigIIIβ, hLigIV/XRCC4, T4 DNA ligase, FEN1 and PARP1 in the presence of different concentrations of compound **19** (Fig. 8 and Supplementary Figs 72 and 73). Cell lysate from MDA-MB-231 cells treated with compound **19** demonstrated the inhibition of ligase activity whereas flap cleavage activity remained the same (Supplementary Fig. 74). This demonstrated the cellular activity of compound **19** and its specific targeting of ligase I inside the cells.

Further, we went ahead and studied the direct binding of compound **19** with hLigI by various biophysical interaction studies such as fluorescence quenching assay, CD and EMSA. Our experiments showed that compound **19** interacts specifically with hLigI but not with DNA (Fig. 9). This specific interaction of compound **19** with hligI also explains the specific antiligase activity of compound **19**.

**Figure 10:**
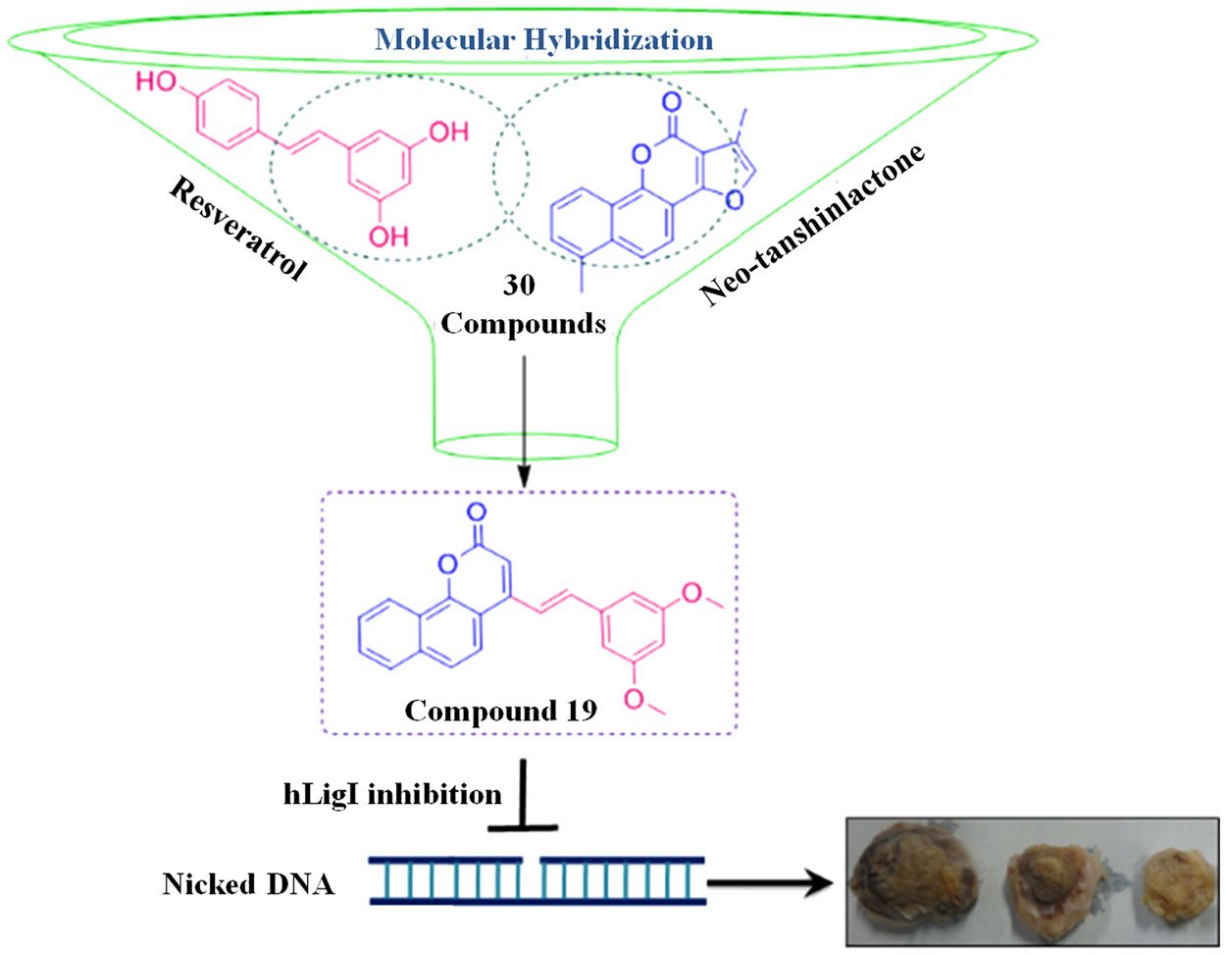
The proposed mode of action of compound **19** on 4T1 induced *in vivo* breast cancer model.

In conclusion, this is the first report of an active hLigI inhibitor with *in-vivo* anti-breast cancer activity. The path to discovery and activity of compound **19** is summarized in Fig. 10. The compound was found to have stable pharmacokinetic parameters and drug like properties to making it a good lead for the synthesis of even better compounds in the future. Therefore, hLigI inhibitors have the potential in the future to help in the treatment of highly metastatic breast cancers that are hormone receptor negative (e.g., TNBC) or otherwise refractory to endocrine treatments. It is likely that in the future, DNA repair proteins (including hLigI inhibitors) will be used as adjuvant therapy with existing chemotherapeutic agents and may likely demonstrate excellent synergistic activity with different DNA damaging agents used in combination therapy^34, 35^. This aspect is currently under active investigation in our laboratory.

## Materials and Methods

### Chemistry

#### Typical procedure for the synthesis of *E*-benzocoumarin-stilbene hybrids (10-39) through Horner-Wadsworth-Emmons (HWE) reaction

To a solution of a suitable phosphonic acid diethyl ester (10–12) (1.0 mmol) in dry DMF (3 mL) at 0 °C MeONa (1.5 mmol) was carefully added. The mixture was stirred at the same temperature for 1 h, then the suitable aldehyde (1.0 mmol) in DMF (3 mL) was added drop wise and the reaction mixture was stirred at room temperature for 1.5 h and heated at 80 °C for 1 h. After completion of the reaction, the cooled reaction mixture was poured in to ice water and left to stand for 3 h. The solid precipitate was obtained by filtration, washed with diethyl ether. Crystallization from ethyl acetate gave the desired stilbene derivative as a pure E-isomer.

#### (E)-4-(3,5-dimethoxystyryl)-2H-benzo[*h*]chromen-2-one (19)

Light yellow solid; yield: 92% mp 180-181 °C; Anal.(%) for C_23_H_18_O_4_: Calcd., C, 77.08; H, 5.06;, found, C, 77.14; H, 5.03; IR (KBr) 3021, 2401, 1706, 1658, 1548, 1215, 928, ν/cm^−1^,^1^H NMR (300 MHz, DMSO-*d*_6_) δ 8.42-8.40 (m, 1 H), 8.25 (d, *J* = 8.8 Hz, 1 H), 8.09-8.06 (m, 1 H), 7.91 (d, *J* = 8.8 Hz, 1 H), 7.82 (d, *J* = 16 Hz, 1 H), 7.75- 7.72 (m, 2 H), 7.63 (d, *J* = 16 Hz, 1 H), 7.04 (d, *J* = 2.2 Hz, 2 H), 6.88 (s, 1 H), 6.55 (t, *J* = 2.1 Hz, 1 H), 3.82 (s, 6 H);^13^C NMR (CDCl_3_, 75 MHz) δ 161.2, 161.1, 151.2, 151.0, 137.8, 137.5, 134.8, 128.7, 127.6, 127.2, 124.1, 123.4, 122.7, 121.4, 120.1, 113.9, 109.9, 105.6, 101.6, 55.5; ESI- MS: (m/z); 358, found [M + H]^+^ 359; HRMS-ESI: C_23_H_19_O_4_ [M + H]^+^ calcd 359.1283, found 359.1284.

### Biological experiments

#### Reagents, media and antibodies

DNA oligos were purchased from Integrated DNA Technologies (IDT, India) MTT (3-[4, 5-dimethylthiazol-2-yl]-2, 5-diphenyltetrazolium bromide), trypsin-EDTA, propidium iodide, DMSO and protease inhibitor cocktail, were purchased from Sigma-Aldrich (St. Louis, MO, USA). Cell growth media DMEM and RPMI were purchased from Sigma-Aldrich (St. Louis, MO, USA), FBS antibiotic and antimycotic solution were purchased from Invitrogen (Whitefield, Bangalore). Fluorescein Isothiocyanate (FITC)-labelled Annexin V (Annexin V-FITC) kit was obtained from BD Biosciences (San Diego, CA). Antibodies against human poly (ADP-ribose) polymerase (PARP), γH2AX, cleaved Caspase 3, Caspase 9, P^53^, and MDM2 were purchased from Cell Signalling Technology (CST, Beverly, MA). PCNA and secondary antibodies were purchased from Santa Cruz Biotechnology (Santa Cruz, CA), β-actin antibody was purchased from Sigma-Aldrich (St. Louis, MO)

#### Cell viability and clonogenic survival assay

Cell viability assay was performed as described previously^19^. Briefly, cells were seeded in 96 well plates and treated with different concentrations of compound **19** for 48 h. After drug treatment, MTT reagent was added in each well of the plate and cell viability was quantified by measuring the absorbance at 590 nm.

Clonogenic survival assay was performed as described previously^16^ with modifications. MDA-MB-231 cells (100 cells) were seeded in 12 well plates for 24 h. Cells were then treated with different concentrations of compound **19** (0.75, 1.5 and 3 µM) for 24 and 48 h. After drug treatment, culture medium was replaced with fresh medium and cells were cultured for another 10 days. After 10 days, the cells were washed with PBS carefully and stained with a solution containing 6% glutaraldehyde and 0.5% crystal violet.

#### Apoptosis and cell cycle distribution analysis

Apoptotic cell death of MDA-MB-231 cells upon treatment with compound **19** was measured using a commercially available Annexin V-FITC apoptosis detection kit according to the manufacturer’s protocol (BD Biosciences, San Diego, CA). Briefly, cells were treated with different concentrations (0, 6 and 12 µM) of compound **19** for 24 and 48 h. The cells were then harvested, washed with cold PBS and stained with Annexin V-FITC and propidium iodide (PI) in binding buffer at room temperature in the dark. The stained cells were analyzed by fluorescence-activated cell sorting using a FACS-Calibur instrument (BD Biosciences, San Diego, CA) equipped with CellQuest 3.3 Software.

For the study of cell cycle distribution, cells were synchronized by serum starvation for 48 h and then grown in serum containing media in the presence or absence of 12 µM compound **19**, after 0, 24 and 48 h, cells were harvested, washed twice with cold PBS and fixed with 70% ethanol. The fixed cells were washed with chilled PBS and then stained with 50 μg/mL of Propidium iodide. Cell cycle distribution was analyzed by using FACS-Calibur (BD Biosciences, San Diago, CA).

#### Scratch assay

The migration capacity of highly metastatic human breast cancer MDA-MB-231 cells was assessed by *in-vitro* scratch assay as described previously^36^ with some modifications. Briefly, cells were plated in 12 well plates and a scratch was created by scraping the cells with a sterile 10 μL pipet tip in the middle of the culture well. After removal of cellular debris, the cells were treated with different concentrations of the test compound. Images of the wound area were captured at 0, 12 and 24 h post-treatment. Wound area was measured by TScratch software and data were expressed as percent (%) open area of control and treated cells.

#### Preparation of cell lysate and Western blotting

Cells (MDA-MB-231) were seeded in 6 well plates and treated either with vehicle control (DMSO) or with different concentrations (6 and 12 µM) of compound **19** for 48 h. After the treatment cell lysate was prepared and western blotting was performed as described previously^37^. Briefly, the cells were lysed by very brief sonication. Sonicated samples were centrifuged and the supernatant was collected and used for ligation assay and western blotting.

#### *In-vitro* stability of compound 19 in simulated gastrointestinal fluids

Simulated gastric fluid (SGF) and simulated intestinal fluid (SIF) were prepared according to USP specifications. The compound **19** was incubated in SGF (0.2 g NaCl, 0.32 g pepsin, 0.7 mL HCl and triple distilled water up to 100 mL, pH 1.2) or SIF (0.680 g KH_2_PO_2_, 0.616 g NaOH, 1 g pancreatin and triple distilled water up to 100 mL, pH 6.8). From the stock solution of compound **19** (1 mg/mL), equivalent concentration of 2 μg/mL of compound **19** were added to 1 mL of preincubated reaction mixture of SGF and SIF, at 37 °C in a shaking water bath in triplicate. 100 μL aliquots of sample was collected at each time point at 0, 5, 15, 30 and 60 min for SGF and 0, 5, 15, 30, 60 90, 120 and 180 min for SIF according to the human gastrointestinal transit time, followed by quenching with acetonitrile, then vortexed and centrifuged at 12000 rpm for 5 min. The supernatant was collected and analyzed by HPLC. The graph was plotted between % drug remaining and incubation time for SGF and SIF.

#### Metabolic stability of compound 19

Major drug clearance in the body occurs due to its metabolism in the liver. Metabolic stability in rat liver microsomes (RLM) was conducted in triplicate. Compound **19** (5 µM) was incubated in reaction milieu which contains RLM (0.5 mg/ml), MgCl_2_ (10 mM), NADPH (10 mM) in a 1.5 ml phosphate buffer solution (pH 7.4). Reaction was started by the addition of NADPH and kept in a shaking water bath at 37 °C. Organic content in reaction mixture was kept less than 1%. Time points at which samples were withdrawn were 0, 5, 15, 30, 45, 60 min. Testosterone was used as control to know the RLM enzyme activity. Control reaction without NADPH was performed to explore any chemical instability or non-cofactor dependent enzymatic degradation. At each time point 150 µL aliquots were withdrawn and added in 150 µL ACN, vortexed at 10,000 g for 5 minutes, 100 µL samples was pipetted out & analyzed in HPLC-PDA.

#### *In- vivo* pharmacokinetic study of compound 19

Male Sprague Dawley (SD) rats weighing 200–220 g were obtained from the Laboratory of Animal Division of CSIR-CDRI (Lucknow, India). Animals were kept in well ventilated cages in hygienic conditions at room temperature (24 ± 2 °C), 40–60% relative humidity and 12 h light-dark cycle. The animals were acclimatized for a minimum period of 3 days prior to the experiments. Pharmacokinetic study was conducted according to protocol approved by the CSIR-CDRI Institutional Animal Ethic Committee (IAEC approval no. IAEC/2012/91). Compound **19** was dissolved in DMSO:Poly Ethylene Glycol-400: 0.9%w/v saline in the ratio of 1:1:2 and was intravenously administered at the dose of 5 mg/kg to each rat (n = 6). The rats were kept on fasting overnight with water alone for oral pharmacokinetic studies. Compound **19** was dissolved in DMSO:Poly Ethylene Glycol-400: 0.5% w/v Sodium carboxymethyl cellulose in TDW in the ratio of 3:1:4 and was orally administered at the dose of 50 mg/kg to each rat (n = 6). Blood samples were collected in microcentrifuge tubes for each time point at 0.083, 0.167, 0.333, 0.5, 0.75, 1.0, 2.0, 4.0, 6.0, 8.0, 10.0, 12.0, 24.0, 48.0 and 72 h post dosing. The blood samples were collected after light ether anesthesia from the retro-orbital plexus of rats using heparin as anticoagulant. Plasma was separated after centrifugation at 5000 rpm for 10 min and all the samples were stored at −80 °C until analysis. Rat plasma samples were processed using liquid-liquid extraction. 100 µL aliquot of plasma mixed with 150 µL of acetonitrile and 100 µL of 5 mM NaHCO_3_ solution was added and vortexed to mix well. In each sample, 2.5 mL of tert-Butyl methyl ether (TBME) (extraction solvent) was added and vortexed for 10 min at 2500 rpm on vibramax (Heidolph, Schwabach, Germany) followed by centrifugation (Eppendorf, Hamburg, Germany) at 5,000 rpm for 5 min. An aliquot of 2.0 mL of supernatant organic layer was separated and evaporated on vacuum concentrator (Thermo scientific, Asheville, USA) to complete dryness. The residue was reconstituted with 100 µL of mobile phase and injected into the LC-MS/MS system for analysis. The plasma samples (100 mL) were assayed along with calibration standards and QC samples and the levels of compound **19** was calculated using Analyst software. The pharmacokinetic profile and parameters were evaluated by non-compartmental model approach using Pheonix 6.3 WinNonlin (Pharsight corporation, USA) including t_1/2_, elimination half-life; C_max_, maximum plasma concentration; t_max_, time to reach maximum plasma concentration; AUC_0-t_, area under concentration curve from zero to last time point.

#### Determination of *in-vivo* tumor regression

Animal studies were conducted under the protocol approved by the CSIR-CDRI Institutional Animal Ethics Committee (IAEC approval no. IAEC/2015/03). The *in-vivo* efficacy studies were performed using BALB/c mice with 4T1 mouse breast cancer induced syngeneic breast tumor model. Mouse tumor model was generated as described by Pulaski *et al*.^38^. Three different groups of 6 mice each were created for the experiment. Mouse breast cancer cells (4T1) were grown in tissue culture flask upto 70% confluence. Cells were then trypsinized, washed with serum containing medium to quench trypsin activity and then washed and resuspended in PBS. Then cells were injected orthotopically (1 × 10^6^ cells in 100 μl PBS) into the mammary fat pad on the right flank of each female BALB/c mouse. Within 2 weeks, palpable tumors were formed and tumor bearing mice were divided randomly into one control and two treatment groups of six mice each. The treatment schedule began on day 16 of the injection and continued for another thirty two days. Compounds were administered by oral gavage once daily at 50 and 100 mg/kg. Tumor volumes were measured every fourth day. The group of vehicle treated animals served as control. The tumor volume, body weight and any behavioral changes in mice were monitored and recorded for the entire duration of the experiment (32 days). Tumor volumes were calculated using the formula (a × b^2^)/2, where a, b are two longest perpendicular diameters.

#### *In-silico* docking studies of compound 19

The crystal structures of DNA replication proteins viz., human DNA Ligase I (hLigI), Human Topoisomerase 1 (Top1), Flap Endonuclease 1 (Fen1), Proliferating cell nuclear antigen (PCNA) and Poly [ADP-ribose] polymerase 1 (PARP1) were retrieved from the PDB [PDB ID: 1 × 9 N, 1T8I, 5FV7, 3WGW and 4ZZZ]. All the crystal structures were pre-processed before docking by eliminating any co-crystallized moieties. Subsequently hydrogen atoms were added and the structures were subjected to an energy minimization step using Poling algorithm with default settings in Sybyl7.1^39^. The structure of compounds was also drawn using Sybyl7.1 followed by an energy minimization of 1000 steps using MMFF94 force field with other default settings. For all selected targets, co-crystal structure of the protein and the inhibitor was available and the inhibitor binding site was used as active site for docking experiments except for hLig1 for which the docking site was selected as done in our previous study^17^.

#### Purification of proteins (hLigI, hLigIIIβ, hLigIV/XRCC4)

The clones for hLigI (_P_RSFDuet-ligI), hLigIIIβ (_P_QE32-hLigIIIβ) and hLigIV/XRCC4 (_P_RSFDuet-hLigIV/XRCC4) were kind gifts from Prof. Alan Tomkinson (University of New Mexico, Albuquerque, USA). hLigI was purified as described previously^19^. The clone for hLigIIIβ was transformed into Rosetta2 (DE3) pLacI cells (Novagen) and the protein was expressed at 16 °C for 18 h with 1 mM IPTG. The clone for hLigIV/XRCC4 was transformed into Rosetta2 (DE3) pLacI cells (Novagen) and the protein was expressed at 25 °C for 12 h with 1 mM IPTG. The proteins were purified following the same procedure as for hLigI.

#### *In-vitro* DNA ligation assay

DNA ligation assay was performed as described previously^17^. Briefly, in the presence of nicked DNA substrate, different concentrations (5, 10 and 20 µM) of compound **19** incubated with different proteins (hLigI, hLigIIIβ, hLigIV/XRCC4 and T4 DNA ligase) at 37 °C for 30 minutes. After the incubation, gel images were captured and analyzed by Image Quant LAS4010 software.

#### *In-vitro* fluorescence quenching assay

*In-vitro* fluorescence quenching assay was performed as described previously^16^ with brief modifications. At the fixed excitation wavelength 280 nm emission spectra of hLigI (100 pmol) was recorded from 300 to 400 nm in the presence (5 and 10 µM) or absence of compound **19** by spectrofluorometer. Compound 19 was used at 5 and 10 µM concentration with hLigI (100 pmol). Spectra was acquired for buffer alone (50 mM Tris-HCl, pH 7.5, 50 mM NaCl, 1 mM ATP, 10 mM MgCl2, 2 mM DTT and 5% glycerol), buffer with vehicle control, buffer with inhibitor alone and for buffer with protein and inhibitor.

#### Circular Dichroism (CD) study

We followed previously described protocol for CD studies^40^ with small modifications. Briefly, emission spectra of hLigI (100 pmol) was recorded at a wavelength of 190-250 nm in the presence (5 and 10 µM) or absence of compound **19** by spectropolarimeter. The raw values in mdeg were obtained and used for mean residual ellipticity [θ] calculation. The graph was generated by the plotting the value of mean residue ellipticity in deg cm^2^ dmol^−1^ with respect to wavelength.

#### Electrophoretic mobility shift assay (EMSA)

EMSA was performed as described previously^17^. Briefly, in 20 μl reaction volume 2 pmol non-ligatable nicked DNA substrate was incubated with DMSO (vehicle control) or with different concentrations (120, 250, 500 µM) of compound **19** incubated with 10 pmol of hligI in ligation buffer. After the incubation, samples were separated by 6.5% native PAGE and bands were detected and imaged by Image Quant LAS 4010 (GE Life sciences).

#### DNaseI cleavage protection assay

We performed DNaseI cleavage assay as described in our previous study^37^. Briefly, 100 ng of plasmid DNA (pUC18) was incubated with 100 µM concentration of doxorubicin (a known DNA intercalator), DAPI (DNA minor groove binder), methyl green (DNA major groove binder) and compound **19** (at 250 and 500 µM concentrations) for 30 min at 37 °C in an incubation buffer similar to ligation buffer. After incubation, the DNA was cleaved by adding 0.0025U of DNaseI in each reaction mixture for 5 min at 37 °C and the reactions were stopped by adding stop buffer which contained 7 M Urea, 20 mM Tris-Cl (pH. 7.5), 50 mM EDTA, 20% glycerol and Bromophenol blue. The samples were resolved on 1% agarose gel and visualized under UV transilluminator and image taken with GE Image quant LAS4010.

## Electronic supplementary material

Supplementary information
