# Supplementary material for: A Novel Benzocoumarin-Stilbene Hybrid as a DNA ligase I inhibitor with in vitro and in vivo anti-tumor activity in breast cancer models
Source: Sci Rep. 2017 Sep 6;7:10715. doi: 10.1038/s41598-017-10864-3 (PMC5587642; doi:10.1038/s41598-017-10864-3)
Supplement: Supplementary file 1 — Supplementary information [file 41598_2017_10864_MOESM1_ESM.doc]

*Supporting Information for*

**A Novel Benzocoumarin-Stilbene Hybrid as a DNA ligase I inhibitor with in vitro and in vivo anti-tumor activity in breast cancer models**

Mohd. Kamil Hussain^a,†‡^, Deependra Kumar Singh^b‡^, Akhilesh Singh^c^, Mohd. Asad^a^, Mohd. Imran Ansari^a^, Mohammad Shameem^b^, Shagun Krishna^b^, Guru R Valicherla^e,d^, Vishal Makadia^f^, Sanjeev Meena^c^, Amit Laxmikant Deshmukh^b^, Jiaur R Gayen^e,d^, Mohammad Imran Siddiqi^b,d^, Dipak Datta^c,d,*^, Kanchan Hajela^a,d,*^, Dibyendu Banerjee^b,d,*^

^‡^Both authors contributed equally.

*Corresponding authors’ email: dipak.datta@cdri.res.in; kanchan_hajela@cdri.res.in; d.banerjee@cdri.res.in

^a^Medicinal and Process Chemistry Division, CSIR-Central Drug Research Institute (CSIR-CDRI), Lucknow-226031, India.

^b^Molecular and Structural Biology Division, CSIR-CDRI, Lucknow-226031, India.

^c^Biochemistry Division, CSIR-CDRI, Lucknow-226031, India.

^d^Academy of Scientific and Innovative Research, CSIR, India.

^e^Pharmacokinetics and Metabolism Division, CSIR-CDRI, Lucknow-226031, India.

^f^Department of Pharmaceutics, National Institute of Pharmaceutical Education and Research, Raibarelly, India.

†Present Addresses: Department of Chemistry Govt. Raza Post Graduate College Rampur-244901, India.

| **Table of Contents** | | |
| --- | --- | --- |
|  | General information | **S2-S3** |
|  | Synthesis of 5-methyl-1-naphthol (3) | **S3-S4** |
|  | Synthesis of 4-bromo-ethylacetoacatate | **S4** |
|  | General procedure for the synthesis of 4-bromomethyl benzcoumarins (4-6) and spectroscopic data | **S4-S5** |
|  | General procedure for the synthesis of compounds 7-9 and spectroscopic data | **S5-S6** |
|  | Typical procedure for the synthesis of compounds 10-39 and spectroscopic data | **S6-S17** |
|  | ^1^H NMR and ^13^C NMR Spectra intermediate compounds 4, 5, 7, 8, 9 | **S18-S22** |
|  | ^1^H NMR and ^13^C NMR Spectra of compounds 10-39 | **S23-S52** |
|  | Biological Experiments | **S53-S57** |
|  | In-silico study of compound 19 with hLigI | **S57-S58** |
|  | Tables | **S59-S64** |
|  | References | **S65-S66** |

**1. General information**

All chemicals of adequate purity were purchased from Sigma-Aldrich and Across Organics, and were used without further purification. ACME silica gel (100-200 mesh) was used for column chromatography and thin-layer chromatography was performed on Merck-precoated silica gel 60-F254 plates. All other chemicals and solvents were obtained from commercial sources and purified using standard methods. Melting points were recorded with COMPLAB melting point apparatus and are uncorrected The IR spectra of all compounds were recorded on a Perkin-Elmer Spectrum GX FTIR spectrometer. The IR values are reported in reciprocal centimeters (cm^-1^). ^1^H NMR and ^13^C NMR spectra were recorded on Bruker DRX-300 (300 MHz for ^1^H and at75 MHz for ^13^C) or DPX-200 (at 50 MHz for ^13^C) spectrometers using CDCl_3_, DMSO-*d*_6_ or TFA-d_1_ (see the Supporting Information). Chemical shifts (δ) are reported in parts per million, using TMS as an internal standard. ESI-MS spectra were recorded on a LCQ Advantage Ion trap mass spectrometer (Finnigan thermo fischer scientific) and High-resolution mass spectra (ESI-HRMS) were recorded on Agilent 6520 ESI-QTOP mass spectrometer. All products reported showed ^1^H NMR and ^13^C NMR spectra in agreement with the assigned structures. Elemental analyses were performed using a Carlo Erba EA-1108 micro analyzer / Vario EL-III C, H, N analyzer and the results obtained were within ± 0.4% of calculated values, confirming their ≥95% purity

**2. Synthesis of 5-methyl-1-naphthol (3)**

**Reagnt and conditions:**(a) MeMgI, ZnCl_2_, THF, rt; (b) Pd/C, triglyme, reflux; (c) BBr_3_,CH_2_Cl_2_

5-Methoxy-1-tetralone (10 g, 56.5 mmol) was dissolved in 160 mL of anhydrous diethyl ether, and methyl magnesium bromide (46 mL, 140 mmol) was slowly added to the solution at 0 ^0^C. The mixture was then heated to reflux and stirred for 3 h. After cooling, the reaction mixture was extracted with diethyl ether. Without further purification the crude product (11 g) was subjected to dehydration and aromatization. 10% Pd/C (12 g) was added to a solution of (**a)** in triglyme (70 mL), and the mixture was heated to reflux for 3 days to furnish compound (**b)** (8.20 g). To a solution of **5c** in anhydrous dichloromethane (100 mL) was added a solution of boron tribromide in dichloromethane (1.0 M, 124 mL) dropwise at 0 ^0^C. The mixture was heated to reflux and stirred for 3h to give the desired naphthol intermediate **3** after silica gel chromatography as solid yield 72% , mp 93-94 ^0^C. (Dong et al., 2010)

**3. Synthesis of 4-bromo-ethylacetoacatate.**

Br_2_ (80 g, 500 mmol) dissolved in acetic acid (500 mL) was added to the mixture of ethylacetoacetate (72 g, 500 mmol) acetic acid (500 mL) in a 5L flask with stirring at room temperature. The mixture was stirred for 3h at room temperature and extracted with DCM, then the organic layer was dried (Na_2_SO_4_) and concentrated in vacuo to give pure 4-bromoethyl acetoacetate (Choi and Chi, 2003).

**4. General procedure for the synthesis of 4-bromomethyl benzcoumarins (4-6)**

A mixture containing 1-napthol (50 mmol), ethyl 4-bromoacetoacetate **1** (80 mmol), and a catalytic amount of conc. H_2_SO_4_ was stirred at room temperature for 3 h. After completion of the reaction ice cold water (200 ml) was added to the reaction mixture, the resulting precipitate was filtered off, washed with diethyl ether and dried under vacuum, providing of 4-bromomethyl benzcoumarins (**4-6**) as a white solid.

**4-(bromomethyl)-2H-benzo[h]chromen-2-one (4):** White solid; yield: 94 %; mp 190- 192 ^0^C. ^1^H NMR (300 MHz, CDCl_3_) δ 8.60-8.58 (m, 1H), 7.92-7.69 (m, 5H), 6.61 (s, 1H), 4.59 (s, 2H), ^13^C NMR (100 MHz, CDCl_3_) δ 159.2, 150.4, 149.8, 133.9, 128.0, 126.3, 123.4, 122.2, 121.6, 118.8, 114.2, 111.6, 26.1; ESI-MS (m/z ):289 found 289 [M ]^+^.

**4-(bromomethyl)-7-methoxy-2H-benzo[h]chromen-2-one (5):** White solid; yield: 85 %; mp 195-196 ^0^C. ^1^H NMR (300 MHz, CDCl_3_) δ 8.19-8.11 (m, 2H), 7.67 (d, *J* = 9.0 Hz, 1H), 7.59-7.75 (m, 1H), 7.01(d, *J* = 7.7 Hz, 1H), 6.61 (s, 1H), 4.58 (s, 2H), 4.05(s, 3H), ^13^C NMR (100 MHz, CDCl_3_) δ 159.3, 154.1, 150.1, 149.8, 126.6, 125.9, 123.2, 117.9, 114.4, 113.5, 112.1, 106.0, 54.7, 26.1; ESI-MS (m/z ): 319, found 319 [M ]^+^.

**4-(bromomethyl)-7-methyl-2H-benzo[h]chromen-2-one (6):** White solid; yield: 98 %; mp 188-189 ^0^C. ^1^H NMR (300 MHz, CDCl_3_) δ 8.47 (d, *J* = 7.6 Hz, 1H), 7.87 (d, *J* = 9.0 Hz, 1H), 7.64 (d, *J = 8.9* Hz, 2H), 7.57-7.48(m, 1H), 6.39 (s, 1H), 4.56 (s, 2H), 2.73 (s, 3H), ^13^C NMR (100 MHz, CDCl_3_) δ 158.3, 156.1, 152.1, 148.4, 134.6, 124.6, 122.2, 116.9, 115.4, 113.8, 112.4, 105.0, 26.1, 20.6; ESI-MS (m/z ):302, found 302 [M ]^+^.

**5. General procedure for the synthesis of phosphonic acid diethyl ester derivatives through Michaelis-Arbusov reaction (7-9):** A mixture of 4-bromomethyl derivative 7-9 (15 mmol) and triethylphosphite (15 mL) was heated at 130 ^0^C for 3 h, after completion of the reaction, reaction mixture was cooled at room temperature and left to stand for 2 h. The solidified reaction mixture was heated with 10 ml DCM was added and diluted with100 ml hexane. The desired compound precipitates white solid that was collected by filtration, washed with hexane and dried.

**diethyl (2-oxo-2H-benzo[h]chromen-4-yl)methylphosphonate (7):** White solid; yield: 95 %; mp 165-166 ^0^C; Anal.(%) for C_18_H_19_O_5_P Calcd., C, 62.43; H, 5.53; found, C, 62.31; H, 5.58; ^1^H NMR (300 MHz, CDCl_3_) δ 8.62-8.58 (m, 1H), 7.91- 7.88 (m, 1H), 7.78-7.72 (m, 2H), 7.68-7.65 (m, 2H), 6.52 (d, *J* = 4.2 Hz, 1H), 4.18-4.08 (m, 4H), 3.43(d, *J* = 23.6 Hz, 2H), 1.29 (t, *J* = 7.0 Hz, 6H); ^13^C NMR (75 MHz, CDCl_3_) δ 160.1(d, *J* = 3.2 Hz), 151.1, 148.0 (d, *J =*9.3 Hz), 134.9, 128.9, 127.6, 127.2, 124.0, 123.1, 122.7, 120.9, 116.3(d, *J* = 8.4Hz), 114.2 (d, *J* = 3.5 Hz), 62.8 (d, *J* = 6.7 Hz), 30.8 (d, *J* = 136.2 Hz), 16.4 (d, *J* = 6.2 Hz); ESI-MS (m/z ):346 found 347 [M+H ]^+^.

**diethyl (7-methoxy-2-oxo-2H-benzo[h]chromen-4-yl)methylphosphonate (8):** Yellow solid; yield: 82 %; mp 159-160 ^0^C; Anal.(%) for C_19_H_21_O_6_P Calcd., C, 60.64; H, 5.62; found,C, 60.46; H, 5.57; ^1^H NMR (300 MHz, CDCl_3_) δ 8.15 (d, *J* = 8.7 Hz, 2H), 7.70 (d, *J* = 9.0 Hz, 1H), 7.59-7.54 (m, 1H), 7.01 (d, *J* = 7.6 Hz, 1H), 6.51 (d, *J* = 4.3 Hz, 1H), 4.17-4.07(m, 4H), 4.05(s, 3H), 3.42 (d, *J* = 23.2 Hz, 2H), 1.28 (t, *J* = 7.0 Hz, 6H); ^13^C NMR (75 MHz, CDCl_3_) δ 160.2 (d, *J* = 3.3 Hz), 150.2, 150.7, 148.0 (d, *J =*9.3 Hz), 127.5, 126.9, 124.2, 120.0, 118.2, 116.5, 116.4, 114.6, 107.0, 62.8 (d, *J* = 6.8 Hz), 55.7, 30.8 (d, *J* = 136.2 Hz), 16.3 (d, *J* = 6.0 Hz); ESI-MS (m/z ):376 found 377 [M+H ]^+^.

**Diethyl(7-methyl-2-oxo-2H-benzo[h]chromen-4-yl)methylphosphonate(9):** White solid; yield: 92 %; mp 155-156 ^0^C; Anal.(%) for C_19_H_21_O_5_P Calcd., C, 63.33; H, 5.87; found,C, 63.41; H, 5.82; ^1^H NMR (300 MHz, CDCl_3_) δ 8.47 (d, *J* = 6.7 Hz, 1H), 7.89 (d, *J* = 7.3 Hz, 1H), 7.77 (d, *J* = 7.4 Hz, 1H ), 7.56-7.51 (m, 2H), 6.51 (s, 1H), 4.12 (m, 4H), 3.42 (d, *J* = 22.8 Hz, 2H), 2.73(s, 3H),1.30 (t, *J* = 6.4 Hz, 6H), ^13^C NMR (75 MHz, CDCl_3_) δ 160.2 (d, *J* = 3.2 Hz), 151.3, 147.9 (d, *J =*9.3 Hz), 134.3, 134.1, 129.6, 126.9, 123.3, 120.8, 120.6, 120.3, 116.2 (d, *J* = 8.3 Hz), 113.9 (d, *J* = 3.5 Hz), 62.8 (d, *J* = 6.7 Hz), 55.7, 30.8 (d, *J* = 136.0 Hz), 16.4 (d, *J* = 5.9 Hz); ESI-MS (m/z ):360 found 361 [M+H ]^+^.

**Typical procedure for the synthesis of *E*-benzocoumarin-stilbene hybrids (10-39) through Horner-Wadsworth-Emmons (HWE) reaction:** To a solution of a suitable phosphonic acid diethyl ester (10–12) (1.0 mmol) in dry DMF (3 mL) at 0 ^0^C MeONa (1.5 mmol) was carefully added. The mixture was stirred at the same temperature for 1 h, then the suitable aldehyde (1.0 mmol) in DMF (3 mL) was added drop wise and the reaction mixture was stirred at room temperature for 1.5 h and heated at 80 ^0^C for 1 h. After completion of the reaction, the cooled reaction mixture was poured in to ice water and left to stand for 3 h. The solid precipitate was obtained by filtration, washed with diethyl ether. Crystallization from ethyl acetate gave the desired stilbene derivative as a pure E-isomer.

**(E)-4-styryl-2H-benzo[h]chromen-2-one (10):** Light yellow solid; yield: 95%; mp 125 ^0^C; Anal.(%) for C_21_H_14_O_2_ Calcd., C, 84.54; H, 4.73; found,C, 84.48; H, 4.78; IR (KBr) 1705, 1382, 1310, 1150, 955; ν/cm^-1^ ^1^H NMR (300 MHz,CDCl_3_) δ 8.63-8.61 (m, 1H), 7.91-7.90 (m,1H), 7.82-7.72 (m, 2H), 7.68-7.63 (m, 4H), 7.50-7.34 (m, 5H), 6.70 (s, 1H); ^13^C NMR (CDCl_3_, + DMSO-*d*_6_,75 MHz) δ 160.5, 151.3, 150.7, 138.5, 136.2, 134.8, 129.8, 129.2, 129.0, 128.4, 128.2, 127.6, 124.3, 123.0, 122.2, 121.5, 120.9, 114.2, 109.21; ESI- MS: (m/z); 298,found [M+H ]^+^ 299; HRMS-ESI: C_21_H_15_O_2_ [M + H]^+^ calcd 299.1072, found 299.1066.

**(E)-4-(4-methoxystyryl)-2H-benzo[h]chromen-2-one (11).** Light yellow solid; yield: 80%; mp 142-144 ^0^C; Anal.(%) for C_22_H_16_O_3_ Calcd., C, 80.47; H, 4.91; found,C, 80.52; H, 4.96; IR (KBr) 2934 1708, 1372, 1312, 1162, 985; ν/cm^-1^, ^1^H NMR (300 MHz,CDCl_3_) δ 8.60 (brs, 1H), 7.89-7.87 (m, 1H), 7.80 (d, *J*= 8.71 Hz, 1H), 7.73 (d, *J*= 8.5 Hz, 1H), 7.67-7.64 (m, 2H), 7.58 (d, *J*= 8.4 Hz, 2H), 7.33 (brs, 2H), 6.99 (d, *J*= 8.3 Hz, 2H), 6.66 (s, 1H), 3.89 (s, 3H); ^13^C NMR (CDCl_3_ + DMSO-*d*_6_ 75 MHz) δ 161.0, 160.6, 151.5, 150.7, 138.4, 134.9, 130.2 129.1, 129.0, 128.3, 127.7, 124.2, 130.2, 123.0, 122.2, 121.6, 118.2, 114.7, 108.1, 55.7; ESI- MS: (m/z); 328, found [M+H ]^+^ 329; HRMS-ESI: for C_22_H_17_O_3_ [M + H]^+^ calcd 329.1178, found 329.1171.

**(E)-4-(4-(dimethylamino)styryl)-2H-benzo[h]chromen-2-one (12)** Light orange solid; yield: 75%; mp 138-139 ^0^C; Anal.(%) for C_23_H_19_NO_2_ : Calcd., C, 80.92; H, 5.61; N, 4.10 found,C, 80.78; H, 5.64; N, 4.12; IR (KBr )2890, 1705, 1344, 1350, 1170, 955; ν/cm^-1^, ^1^H NMR (300 MHz,CDCl_3_) δ 8.62 (brs, 1H), 7.87-7.83 (m, 2H), 7.73 (d, *J* = 8.8 Hz, 1H), 7.66-7.65 (m, 2H), 7.54 (d, *J* = 8.3 Hz, 2H), 7.37 (d, *J* = 15.8 Hz, 1H), 7.22 (s, 1H), 6.75 (d, *J* = 8.2 Hz, 2H), 6.66 (s, 1H), 3.06 (s, 6H); ^13^C NMR (CDCl_3_, 50 MHz) δ 161.6, 151.6, 151.3, 151.0, 138.3, 134.6, 129.1, 128.4, 127.5, 126.9, 123.8, 123.6, 123.4, 122.7, 120.2, 114.9, 114.2, 112.0, 107.3, 40.1; ESI- MS: (m/z); 341,found [M+H ]^+^ 342; HRMS-ESI: C_23_H_20_NO_2_ [M + H]^+^ calcd 342.1494, found 342.1502.

**(E)-4-(4-nitrostyryl)-2H-benzo[h]chromen-2-one (13)** Light yellow solid; yield: 96%, mp-212 ^0^C; Anal.(%) for C_21_H_13_NO_4_ : Calcd., C, 73.46; H, 3.82; N, 4.08, found, C, 73.55; H, 3.71; N, 4.0; ^1^H NMR (300 MHz, CDCl_3_ +TFA-*d*) δ 8.53-8.51 (m, 1H), 8.35 (d, *J* = 8.7 Hz, 2H), 7.97-7.95 (m, 1H), 7.86-7.66 (m, 7H), 7.51 (d, *J* = 16.2 Hz, 1H), 6.95 (s, 1H); ^13^C NMR (CDCl_3_ TFA-d_1_ 75 MHz) δ 161.2, 161.1, 153.5, 150.8, 148.1, 141.5, 136.9, 135.3, 129.9, 128.4, 123.0, 122.4, 119.9, 119.4, 116.2, 114.4, 112.4, 108.8, 108.6, ESI- MS: (m/z); 343,found [M+H ]^+^ 344; HRMS-ESI: C_21_H_14_NO_4_ [M + H]^+^ calcd 344.0923, found 344.0902.

**(E)-4-(2-(2-oxo-2H-benzo[h]chromen-4-yl)vinyl)benzonitrile (14).** Light yellow solid; yield: 92%, mp ^0^C; Anal.(%) for C_22_H_13_NO_2_ : Calcd., C, 81.72 ; H, 4.05; N, 4.33, found, C, 81.67; H, 4.08; N, 4.35; ^1^H NMR (300 MHz, CDCl_3_) δ 8.63-8.60 (m, 1H), 7.92-7.90 (m, 1H), 7.75- 7.68 (m, 8H), 7.58 (d, *J* = 15.9 Hz, 1H), 7.34 (d, *J* = 15.9 Hz, 1H), 6.71 (s, 1H); ^13^C NMR (CDCl_3_, 75 Hz) δ 161.0, 151.7, 150.6, 140.0, 135.7, 135.1, 132.9, 129.1, 128.1, 127.9, 124.9, 124.5, 123.5, 122.9, 120.1, 118.6, 113.7, 112.9, 111.0; ESI- MS: (m/z); 323,found 324 [M+H ]^+^; HRMS-ESI: [M + H]^+^ C_22_H_14_NO_2_ calcd. 324.1025, found 324.1038.

**(E)-4-(3-methoxystyryl)-2H-benzo[h]chromen-2-one (15):** Light yellow solid; yield : 85%, mp 145-146 ^0^C; Anal.(%) for C_22_H_16_O_3_ : Calcd., C, 80.47; H, 4.91;, found, C, 80.53; H, 4.88; IR (KBr ) ν/cm^-1^, 2994, 1702, 1385, 1316, 1155, 965; ^1^H NMR (300 MHz, CDCl_3_) δ 8.52 (brs, 1H), 7.80-758 (m, 5H), 7.40-7.29 (m, 2H), 7.22-7.06 (m, 3H), 6.88 (d, *J*= 8.2 Hz, 1H), 6.60 (s, 1H), 3.81 (s, 3H); ^13^C NMR (CDCl_3_ 75 MHz) δ 161.1, 160.0, 151.1, 151.0, 137.6, 137.0, 134.7 130.0, 128.7, 127.6, 127.1, 124.1, 123.3, 122.7, 121.1, 120.1, 115.1, 112.9, 109.7, 55.4; ESI- MS: (m/z); 328, found [M+H ]^+^ 329; HRMS-ESI: C_22_H_17_O_3_ [M + H]^+^ calcd 329.1178, found 329.1177.

**(E)-4-(2,6-dimethoxystyryl)-2H-benzo[*h*]chromen-2-one (16):** Light yellow solid; yield, 90% mp 174-175 ^0^C; Anal.(%) for C_23_H_18_O_4_: Calcd., C, 77.08; H, 5.06;, found, C, 77.16; H, 5.0; IR (KBr) 2998, 2395, 1712, 1668, 1592, 1218, 938 ν/cm^-1^, ^1^H NMR (300 MHz, CDCl_3_) δ 8.64-8.61 (m, 1H), 7.99 (d, *J* = 16.2 Hz, 1H), 7.91-7.88 (m, 1H),7.83-7.78 (m, 2H), 7.73 (d, *J* = 8.8 Hz, 1H), 7.67-7.64 (m, 2H), 7.32 (d, *J* = 8.4 Hz, 1H), 6.75 (s, 1H), 6.64 (d, *J* = 8.1 Hz, 2H), 3.97 (s, 6H); ^13^C NMR (CDCl_3_, 75 MHz) δ 161.2, 159.4, 153.5, 150.9, 134.7, 130.5, 128.9, 127.5, 126.9, 123.8, 123.4, 122.7, 120.4, 120.6, 113.4, 106.5, 103.56, 55.9; ESI- MS: (m/z); 358, found [M+H ]^+^ 359; HRMS-ESI: C_23_H_19_O_4_ [M + H]^+^ calcd 359.1283, found 359.1289.

**(E)-4-(3,4-dimethoxystyryl)-2H-benzo[*h*]chromen-2-one (17):** Light yellow solid; yield : 82%, mp 180-182 ^0^C; Anal.(%) for C_23_H_18_O_4_ : Calcd., C, 77.08; H, 5.06;, found, C, 77.18; H, 5.03; IR (KBr) 3021, 2405, 1704, 1648, 1544, 1212, 924 ν/cm^-1^, ^1^H NMR (300 MHz, CDCl_3_) δ 8.62-8.59 (m, 1H), 7.91-7.88 (m, 1H), 7.82 (d, *J* = 8.7, 1H), 7.74 (d, *J* = 8.7 Hz, 1H), 7.67-7.64 (m, 2H), 7.32 (s, 2H), 7.21-7.16 (m, 2H), 6.95 (d, *J* = 8.2 Hz, 1H), 6.67 (s, 1H), 4.01 (s, 3H), 3.96 (s, 3H); ^13^C NMR (CDCl_3_, 75 MHz) δ 160.6, 151.6, 150.9, 150.7, 149.5, 138.8, 134.8, 129.2, 129.0, 128.2, 127.6, 124.1, 123.0, 122.2, 121.7, 118.3, 114.4, 111.9, 110.7, 108.1, 56.2, 56.0; ESI- MS: (m/z); 358, found [M+H ]^+^ 359; HRMS-ESI: C_23_H_19_O_4_ [M + H]^+^ calcd 359.1283, found 359.1286.

**(E)-4-(3,4,5-trimethoxystyryl)-2H-benzo[*h*]chromen-2-one (18).** Light yellow solid; yield, 86% mp 172-173 ^0^C; Anal.(%) for C_24_H_20_O_5_: Calcd., C, 74.21; H, 5.19;, found, C, 74.32; H, 5.17; IR (KBr) 3012, 2385, 1702, 1654, 1538, 1210, 922 ν/cm^-1^, ^1^H NMR (300 MHz,CDCl_3_) δ 8.63-8.60 (m, 1H), 7.91-7.89 (m, 1H), 7.82-7.73 (m, 2H), 7.69-7.66 (m, 2H), 7.37 (d, *J* = 15.9 Hz, 1H), 7.31 (s, 1H), 6.85 (s, 2H), 6.67 (s, 1H), 3.98 (s, 6H), 3.93 (s, 3H); ^13^C NMR (CDCl_3_, 75 MHz) δ 161.2, 153.6 151.1, 151.0, 139.7, 137.8, 134.7, 131.2, 128.7, 127.6, 127.1, 124.0, 123.3, 122.7, 120.2, 120.1, 113.9, 109.4, 104.9, 61.0, 56.3; ESI- MS: (m/z); 388, found [M+H ]^+^ 389; HRMS-ESI: C_24_H_21_O_5_ [M + H]^+^ calcd 389.1389, found 389.1392.

**(E)-4-(3,5-dimethoxystyryl)-2H-benzo[*h*]chromen-2-one (19)** Light yellow solid; yield: 92% mp 180-181 ^0^C; Anal.(%) for C_23_H_18_O_4_ : Calcd., C, 77.08; H, 5.06;, found, C, 77.14; H, 5.03; IR (KBr) 3021, 2401, 1706, 1658, 1548, 1215, 928, ν/cm^-1^, ^1^H NMR (300 MHz, DMSO-*d*_6_) δ 8.42-8.40 (m, 1H), 8.25 (d, *J* = 8.8 Hz, 1H), 8.09-8.06 (m, 1H), 7.91 (d, *J* = 8.8 Hz, 1H), 7.82 (d, *J* = 16 Hz, 1H), 7.75- 7.72 (m, 2H), 7.63 (d, *J* = 16 Hz, 1H), 7.04 (d, *J* = 2.2 Hz, 2H), 6.88 (s, 1H), 6.55 (t, *J* = 2.1 Hz, 1H), 3.82 (s, 6H); ^13^C NMR (CDCl_3_, 75 MHz) δ 161.2, 161.1, 151.2, 151.0, 137.8, 137.5, 134.8, 128.7, 127.6, 127.2, 124.1, 123.4, 122.7, 121.4, 120.1, 113.9, 109.9, 105.6, 101.6, 55.5; ESI- MS: (m/z); 358, found [M+H ]^+^ 359; HRMS-ESI: C_23_H_19_O_4_ [M + H]^+^ calcd 359.1283, found 359.1284.

**(E)-4-(2,5-dimethoxystyryl)-2H-benzo[*h*]chromen-2-one (20):** Light yellow solid; mp 168-70 ^0^C; Anal.(%) for C_23_H_18_O_4_: Calcd., C, 77.08; H, 5.06;, found, C, 77.14; H, 5.10; IR (KBr) 3014, 2404, 1708, 1628, 1528, 1222, 924 ν/cm^-1^, ^1^H NMR (300 MHz, CDCl_3_) δ 8.63-8.60 (m, 1H), 7.91-7.88 (m, 1H), 7.86 (d, *J* = 8.8 Hz, 1H), 7.74-7.72 (m, 1H), 7.68-7.65 (m, 3H), 7.51 (d, *J* = 16.1 Hz, 1H), 7.21(d, *J* = 2.4 Hz, 1H), 6.94-6.92 (m, 2H), 6.73 (s, 1H), 3.91 (s, 3H), 3.87 (s, 3H); ^13^C NMR (CDCl_3_, 75 MHz) δ 161.1, 153.6, 152.3, 151.7, 151.0, 134.7, 132.9, 128.6, 127.6, 127.1, 125.3, 124.0, 123.3, 122.7, 121.4, 120.2, 115.8, 114.0, 112.3, 109.4, 56.1, 55.8; ESI- MS: (m/z); 358, found [M+H ]^+^ 359; HRMS-ESI: C_23_H_19_O_4_ [M + H]^+^calcd 359.1283, found 359.1281.

**(E)-4-(2,4-dimethoxystyryl)-2H-benzo[*h*]chromen-2-one (21):** Light yellow solid; yield 78%, mp 181-183 ^0^C; Anal.(%) for C_23_H_18_O_4_: Calcd., C, 77.08; H, 5.06;, found, C, 77.20; H, 5.04; IR (KBr) 3008, 2411, 1701, 1638, 1542, 1210, 948 ν/cm^-1^, ^1^H NMR (300 MHz,CDCl_3_) δ 8.61 (brs, 1H), 7.89-7.81 (m, 2H), 7.74-7.65 (m, 4H), 7.59 (d, *J* = 8.8 Hz, 1H), 7.44 (d, *J* = 16.1 Hz, 1H), 6.71 (s, 1H), 6.59 (d, *J* = 8.4 Hz, 1H), 6.52 (s, 1H), 3.94 (s, 3H), 3.89 (s, 3H); ^13^C NMR (CDCl_3_, 75 MHz) δ 162.2, 161.1, 159.2, 152.1, 150.9, 134.7, 133.0, 129.2, 128.5, 127.5, 127.0, 123.4, 122.7, 120.3, 118.4, 117.9, 114.2, 108.3, 105.4, 98.4, 55.6, 55.4; ESI- MS: (m/z); 358,found [M+H ]^+^ 359; HRMS-ESI: C_23_H_19_O_4_ [M + H]^+^ calcd 359.1283, found 359.1382.

**(E)-7-methoxy-4-styryl-2H-benzo[*h*]chromen-2-one (22):** Light yellow solid; yield: 92%, mp178-179 ^0^C; Anal.(%) for C_22_H_16_O_3_: Calcd., C, 80.47; H, 4.91, found, C, 80.56; H, 4.88; IR (KBr ) 3022, 2398, 1708, 1591, 1548, 1215, 928ν/cm^-1^, ^1^H NMR (300 MHz, CDCl_3_) δ 8.19-8.14 (m, 2H), 7.77 (d, *J* = 8.9 Hz, 1H), 7.66-7.55 (m, 3H), 7.52-7.45 (m, 4H), 7.37 (d, *J* = 15.9 Hz, 1H),7.02 (d, *J* = 7.6 Hz, 1H), 6.71 (s, 1H), 4.05 (s, 3H); ^13^C NMR (CDCl_3_, 75 MHz) δ 161.3, 155.1, 151.2, 150.7,137.7, 135.7, 129.6, 129.0, 127.6, 127.4, 126.8, 124.4, 120.9, 119.2, 118.3, 114.7, 114.4, 109.9, 106.8, 55.7; ESI- MS: (m/z); 328,found [M+H ]^+^ 329; HRMS-ESI: C_22_H_17_O_3_ [M + H]^+^ calcd 329.1178, found 329.1176.

**(E)-7-methoxy-4-(4-methoxystyryl)-2*H*-benzo[h]chromen-2-one (23):** Light yellow solid; yield: 80% mp 170-171 ^0^C; Anal.(%) for C_23_H_18_O_4_: Calcd., C, 77.08; H, 5.06, found, C, 77.21; H, 5.02; IR (KBr) 3022, 2392 1710, 1592, 1538, 1218, 938 ν/cm^-1^, ^1^H NMR (300 MHz, CDCl_3_) δ 8.21-8.13 (m, 2H), 7.82 (d, *J* = 9.0 Hz, 1H), 7.63-7.60 (m, 3H), 7.44 (d, *J* = 16.1 Hz, 1H), 7.34 (s, 1H), 7.05-6.98 (m, 3H), 6.86 (s, 1H), 4.06 (s, 3H), 3.90 (s, 3H); ^13^C NMR (CDCl_3_, 75 MHz) δ 161.4, 160.8, 155.1, 151.3, 150.7,137.3, 129.0, 129.0, 128.4, 127.3, 126.7, 124.4, 119.2, 118.3, 118.1, 114.7, 114.4, 108.9, 106.7, 55.6, 55.4; ESI- MS: (m/z); 358,found [M+H ]^+^ 359; HRMS-ESI: C_23_H_19_O_4_ [M + H]^+^ calcd 359.1283, found 359.1290.

**(E)-7-methoxy-4-(4-nitrostyryl)-2H-benzo[*h*]chromen-2-one (24):** Light yellow solid; yield 94%, mp 220-222 ^0^C; Anal.(%) for C_22_H_15_NO_5_: Calcd., C, 70.77; H, 4.05; N, 3.75; found, C, 70.64; H, 4.0; N, 3.73; ^1^H NMR (300 MHz, CDCl_3_ + TFA-*d*_1_) δ 8.37-8.30 (m, 3H), 8.08 (d, *J* = 8.4 Hz, 1H), 7.86-7.82 (m, 3H), 7.75-7.63 (m, 2H), 7.54 (d, *J* = 15.9 Hz, 1H), 7.13 (d, *J* = 7.8 Hz, 1H), 6.99 (s, 1H), 4.09 (s, 3H); ^13^C NMR (CDCl_3_ + TFA-*d*_1_,75 MHz) δ 162.5, 155.1, 154.2, 150.4, 147.9, 141.6, 137.1, 135.7, 128.6, 128.5, 127.3, 124.4, 124.0, 120.4, 119.8, 118.4, 114.6, 114.2, 108.5, 108.3, 55.8; ESI- MS: (m/z); 373, found [M+H]^+^ 374; HRMS-ESI: C_22_H_16_NO_5_ [M + H]^+^ calcd 374.1028, found 374.1022.

**(E)-4-(2-(7-methoxy-2-oxo-2H-benzo[*h*]chromen-4-yl)vinyl)benzonitrile (25):** Light yellow solid; yield: 92%, Anal.(%) for C_23_H_15_NO_3_: Calcd., C, 78.17; H, 4.28; N, 3.96; found, C, 78.34; H, 4.25; N, 3.95; mp 188-189 ^0^C, ^1^H NMR (300 MHz, TFA-*d*_1_) δ 8.00 (brs, 1H), 7.69-7.56 (m, 6H), 7.41-7.21 (m, 3H), 6.87 (brs, 1H), 6.70 (brs, 1H), 3.81 (s, 3H); ^13^C NMR (TFA-*d*_1_,75 MHz) δ 166.9, 154.7, 154.4, 149.9, 139.9, 137.4, 132.4, 127.9, 127.6, 126.8, 123.1, 122.4, 119.9, 119.6, 117.4, 115.89, 114.0, 110.8, 106.4, 54.8; ESI- MS: (m/z); 353, found [M+H ]^+^ 354; HRMS-ESI: [M + H]^+^ C_23_H_16_NO_3_ calcd 354.1130, found 354.1128.

**(E)-4-(3,5-dimethoxystyryl)-7-methoxy-2*H*-benzo[*h*]chromen-2-one** (**26)** Light yellow solid; yield: 84%, mp 175-176 ^0^C; Anal.(%) for C_24_H_20_O_5_: Calcd., C, 74.21; H, 5.19; found, C, 74.32; H, 5.14; IR (KBr) 3434, 2394, 1708, 1591, 1385, 955ν/cm^-1^, ^1^H NMR (300 MHz, CDCl_3_) δ 8.19-8.14 (m, 2H), 7.75 (d, *J* = 8.9 Hz, 1H), 7.60-7.54 (m, 1H), 7.45 (d, *J* = 15.8 Hz, 1H), 7.31-7.26 (m, 1H), 7.02 (d, *J* = 7.7 Hz, 1H), 6.78 (d, *J* = 1.9 Hz, 2H), 6.68 (s, 1H), 6.53(s, 1H), 4.05 (s, 3H), 3.89 (s, 6H); ^13^C NMR (CDCl_3_, 75 MHz) δ 161.1, 155.1, 150.7, 137.7, 137.5 127.4, 126.7, 124.4, 121.5, 119.2, 118.3, 114.7, 114.6, 114.3, 110.0, 106.8, 105.6, 55.7, 55.5 ESI- MS: (m/z); 388, found [M+H ]^+^ 389; HRMS-ESI: C_24_H_21_O_5_ [M + H]^+^ calcd 389.1389, found 389.1378.

**(E)-4-(3,4-dimethoxystyryl)-7-methoxy-2*H*-benzo[*h*]chromen-2-one (27):** Light yellow solid; yield 82%, mp182-183 ^0^C; Anal.(%) for C_24_H_20_O_5_: Calcd., C, 74.21; H, 5.19; found, C, 74.18; H, 5.23; IR (KBr) 3426, 2384, 1706, 1592, 1384, 958 ν/cm^-1^, ^1^H NMR (300 MHz, CDCl_3_) δ 8.20-8.14 (m, 2H), 7.79 (d, *J* = 8.9 Hz, 1H), 7.57 (t, *J* = 8.0 Hz, 1H), 7.33 (s, 2H), 7.22-7.16 (m, 2H), 7.01 (d, *J* = 7.7 Hz, 1H), 6.95 (d, *J* = 8.2 Hz, 1H), 6.68 (s, 1H), 4.05 (s, 3H), 4.01 (s, 3H), 3.96 (s, 3H); ^13^C NMR (CDCl_3_, 75 MHz) δ 160.4, 154.0, 150.2, 149.6, 149.5, 148.3, 136.6, 127.7, 126.3, 125.7, 123.4, 120.6, 117.5, 117.1, 113.6, 113.4, 110.2, 108.5, 108.0, 105.7, 55.03, 55.00, 54.69; ESI- MS: (m/z); 388, found [M+H ]^+^ 389; HRMS-ESI: C_24_H_21_O_5_ [M + H]^+^, calcd 389.1389, found 389.1381.

**(E)-4-(2,6-dimethoxystyryl)-7-methoxy-2*H*-benzo[*h*]chromen-2-one (28):** Light yellow solid; yield 78%, mp, 178-179 ^0^C; Anal.(%) for C_24_H_20_O_5_: Calcd., C, 74.21; H, 5.19; found, C, 74.28; H, 5.17; IR (KBr) ν/cm^-1^, 3418, 2398, 1701, 1592, 1384, 954, ^1^H NMR (300 MHz, CDCl_3_) δ 8.20-8.12 (m, 2H), 8.00 (d, *J* = 16.1 Hz, 1H), 7.82-7.77 (m, 2H), 7.56 (t, *J* = 8.1 Hz, 1H), 7.38-7.30 (m, 1H) ,7.00 (d, *J* = 7.7 Hz, 1H), 6.75 (s, 1H), 6.64 (d, *J* = 8.4, 2H), 4.05 (s, 3H), 3.96 (s, 6H); ^13^C NMR (CDCl_3_, 75 MHz) δ 160.8, 158.4, 154.1, 152.4, 149.5, 129.4, 127.8, 126.1, 125.6, 13.4, 122.9, 118.7, 116.9, 113.8, 113.7, 112.3, 107.6, 105.5, 102.8, 54.6, 54.6; ESI- MS: (m/z); 388, found [M+H]^+^ 389; RMS-ESI: for C_24_H_21_O_5_ [M + H]^+^ calcd 389.1389, found 389.1378.

**(E)-4-(2,5-dimethoxystyryl)-7-methoxy-2*H*-benzo[*h*]chromen-2-one (29):** Light yellow solid; yield 75%, mp176-177 ^0^C; Anal.(%) for C_24_H_20_O_5_: Calcd., C, 74.21; H, 5.19; found, C, 74.12; H, 5.21; IR (KBr) 3402, 2382, 1705, 1596, 1375, 945 ν/cm^-1^, ^1^H NMR (300 MHz, CDCl_3_) δ 8.19-8.13 (m, 2H), 7.78 (d, *J* = 9.1 Hz, 1H), 7.71 (d, *J* = 16.1 Hz, 1H), 7.58 (d, *J* = 7.9 Hz, 1H), 7.50 (d, *J* = 16.4 Hz, 1H), 7.21 (d, *J* = 1.4 Hz, 1H), 7.01 (d, *J* = 7.6, Hz, 1H), 6.96-6.89 (m, 2H), 6.72 (s, 1H), 4.05 (s, 3H), 3.90 (s, 3H), 3.87 (s, 3H); ^13^C NMR (CDCl_3_, 75 MHz) δ 160.4, 154.0, 152.6, 151.2, 150.6, 149.6, 131.7, 126.3, 125.7, 124.3, 123.3, 120.4, 118.3, 117.1, 114.1, 113.6, 113.4, 112.0, 111.3, 108.5, 105.6, 55.1, 54.8, 54.6; ESI- MS: (m/z); 388, found [M+H ]^+^ 389; HRMS-ESI: C_24_H_21_O_5_[M + H]^+^ calcd 389.1389, found 389.1390.

**(E)-4-(2,4-dimethoxystyryl)-7-methoxy-2H-benzo[h]chromen-2-one (30):** Light yellow solid; yield 72% mp 184 ^0^C; Anal.(%) for C_24_H_20_O_5_: Calcd., C, 74.21; H, 5.19; found, C, 74.34; H, 5.19; IR (KBr) 3418, 2392, 1708, 1591, 1385, 955 ν/cm^-1^, ^1^H NMR (300 MHz, CDCl_3_) δ 8.20-8.12 (m, 2H), 7.79 (d, *J* = 9.1 Hz, 1H), 7.68 (d, *J* = 15.9 Hz, 1H), 7.61-7.53(m, 2H), 7.44 (d, *J* = 15.9 Hz, 1H), 7.06 (d, *J* = 7.6 Hz, 1H), 6.71 (s, 1H), 6,60-6.57 (m, 1H), 6.53 (d, *J* = 2.0 Hz, 1H), 4.05 (s, 3H), 3.94 (s, 3H), 3.89 (s, 3H); ^13^C NMR (CDCl_3_, 75 MHz) δ 162.2, 161.1, 159.1, 155.1, 152.0, 150.6, 132.9, 129.1, 127.2, 126.6, 124.4, 119.4, 118.5, 118.0, 117.8, 114.7, 114.6, 108.9, 106.6, 105.4, 98.4, 55.7, 55.6, 55.5; ESI- MS: (m/z); 388, found [M+H ]^+^ 389; HRMS-ESI: C_24_H_21_O_5_ [M + H]^+^calcd. 389.1389, found 389.1378

**(E)-7-methoxy-4-(3,4,5-trimethoxystyryl)-2*H*-benzo[*h*]chromen-2-one (31):** Light yellow solid; mp 205-206 ^0^C; Anal.(%) for C_25_H_22_O_6_: Calcd., C, 71.76; H, 5.30; found, C, 71.66; H, 5.33; IR (KBr) 3412, 2382, 1702, 1571, 1386, 952 ν/cm^-1^, ^1^H NMR (300 MHz, CDCl_3_) δ 8.18-8.13 (m, 2H), 7.76 (d, *J* = 9.0 Hz, 1H), 7.56 (t, *J* = 7.9 Hz, 1H), 7.36 (d, *J* = 15.8 Hz, 1H), 7.30-7.25 (m, 1H), 7.01 (d, *J* = 7.7 Hz, 1H), 6.85 (s, 2H), 6.66 (s, 1H), 4.05 (s, 3H), 3.98 (s, 6H), 3.93 (s, 3H); ^13^C NMR (CDCl_3 +_DMSO-d_6_ 75 MHz) δ 160.7, 155.4, 151.6, 151.0, 150.6, 149.7, 139.0, 129.4, 128.3, 126.6, 124.2, 123.2, 121.1, 118.4, 118.0, 114.9, 114.2, 112.1, 110.9, 108.4, 107.8, 55.7, 56.4, 56.2; ESI- MS: (m/z); 418, found [M+H ]^+^  419; HRMS-ESI: C_25_H_23_O_6_ [M + H]^+^ calcd 419.1495, found 419.1498.

**(E)-7-methyl-4-styryl-2*H*-benzo[*h*]chromen-2-one (32):** Light yellow solid; mp 178-179 ^0^C; Anal.(%) for C_22_H_16_O_2_: Calcd., C, 84.59; H, 5.16; found, C, 84.38; H, 5.19; IR (KBr) 3228, 2282, 1710, 1578, 1396, 958 ν/cm^-1^, ^1^H NMR (300 MHz, CDCl_3_) δ 8.50 (d, *J* = 7.9 Hz, 1H), 7.91-7.79 (m, 2 H), 7.68-7.63 (m, 2H), 7.58-7.45 (m, 5H), 7.38 (d, *J* = 15.9 Hz, 1H), 7.21 (brs, 1H), 6.70 (s, 1H), 2.74(s, 3H); ^13^C NMR (CDCl_3_, 75 MHz) δ 160.5, 150.4, 150.3, 136.9, 134.8, 133.4, 133.1, 128.8, 128.6, 128.2, 126.7, 126.0, 122.6, 120.1, 120.0, 119.5, 112.7, 108.8, 18.6; ESI- MS: (m/z); 312, found [M+H ]^+^ 313; HRMS-ESI: C_22_H_17_O_2_ [M + H]^+^ calcd 313.1229, found 313.1224.

**(E)-7-methyl-4-(4-nitrostyryl)-2*H*-benzo[*h*]chromen-2-one (33):** Light yellow solid; mp 238-239 ^0^C; Anal.(%) for C_22_H_15_NO_4_: Calcd., C, 73.94; H, 4.23; N, 3.92, found, C, 73.82; H, 4.26, N, 3.9; ^1^H NMR (300 MHz, CDCl_3_ + TFA-*d*_1_) δ 8.38-8.35 (m, 3H), 8.06 (d, *J* = 8.9 Hz, 1H), 7.91-7.84 (m, 3H), 7.73 (d, *J* = 16.0 Hz, 1H), 7.66 (m, 3H), 6.98 (s, 1H), 2.78 (s, 3H); ^13^C NMR (CDCl_3_, 75 MHz) δ 162.4, 161. 8, 161.2, 160.8, 154.9, 151.0, 147.9, 141.6, 137.1, 134.9, 134.5, 130.7, 127.9, 124.4, 123.1, 120.4, 119.8, 119.0, 116.0, 113.8, 112.3, 108.5, 108.3, 19.2; ESI- MS: (m/z); 357, found [M+H ]^+^ 358; HRMS-ESI: C_22_H_16_NO_4_ [M + H]^+^ calcd 358.1079, found 358.1086

**(E)-4-(3,5-dimethoxystyryl)-7-methyl-2*H*-benzo[*h*]chromen-2-one (34):** Light yellow solid; mp190-191 ^0^C; Anal.(%) for C_24_H_20_O_4_: Calcd., C, 77.40; H, 5.41; found, C, 77.35; H, 5.48; IR (KBr) 3428, 2378, 1706, 1578, 1386, 948 ν/cm^-1^, ^1^H NMR (300 MHz, CDCl_3_) δ 8.50 (d, *J* = 7.7 Hz, 1H), 7.89 (d, *J* = 7.9 Hz, 1H), 7.80 (d = 8.8 Hz,1H), 7.58-7.49 (m, 2H), 7.45 (d, *J* = 15.7 Hz, 1H), 7.31 (s, 1H), 6.78 (s, 2H), 6.68 (s, 1H), 6.53 (s, 1H), 4.05 (s, 3H), 3.89 (s, 6H), 2.74 (s, 3H); ^13^C NMR (CDCl_3_, 75 MHz) δ 161.3, 161.2, 151.3, 151.0, 137.7, 137.6, 134.3, 134.0, 129.5, 126.9, 123.5, 121.5, 120.9, 120.4, 119.8, 113.6, 109.8, 105.6, 101.6, 55.5, 19.5; ESI- MS: (m/z); 372, found [M+H ]^+^ 373; HRMS-ESI: [M + H]^+^ for C_24_H_21_O_4_ calcd 373.1440, found 373.1442.

**(E)-4-(2,4-dimethoxystyryl)-7-methyl-2H-benzo[h]chromen-2-one (35):** Light yellow solid; mp 85-186 ^0^C; Anal.(%) for C_24_H_20_O_4_: Calcd., C, 77.40; H, 5.41; found, C, 77.38; H, 5.45; IR (KBr) 3412, 2348, 1704, 1578, 1378, 958 ν/cm^-1^, ^1^H NMR (300 MHz, CDCl_3_) δ 8.51 (d, *J* = 8.3 Hz, 1H), 7.89-7.82 (m, 2H), 7.69 (d, *J* = 16.0 Hz, 1H), 7.61-7.57 (m, 1H), 7.54-7.47 (m, 2H), 7.45 (d, *J* = 16.2 Hz, 1H), 6.70 (s, 1H), 6.61-6.57 (m, 1H), 6.53 (d, *J* = 1.8 Hz, 1H), 3.94, (s, 3H), 3.89 (s, 3H), 2.74 (s, 3H); ^13^C NMR (CDCl_3_, 75 MHz) δ 161.2, 160.6, 158.2, 150.9, 150.2, 132.2, 132.8, 131.9, 128.2, 128.24, 125.6, 122.5, 119.9, 119.1, 118.9, 117.5, 116.9, 112.8, 107.2, 104.4, 97.4, 54.5, 54.4, 18.4; ESI- MS: (m/z); 372, found [M+H ]^+^ 373; HRMS-ESI: [M + H]^+^ for C_24_H_21_O_4_ calcd 373.1440, found 373.1447.

**(E)-4-(2,5-dimethoxystyryl)-7-methyl-2H-benzo[h]chromen-2-one (36)** Light yellow solid; mp 168-169 ^0^C; Anal.(%) for C_24_H_20_O_4_: Calcd., C, 77.40; H, 5.41; found, C, 77.28; H, 5.43; IR (KBr) ν/cm^-1^, ^1^H NMR (300 MHz, CDCl_3_) δ 8.58 (d, *J* = 8.0 Hz, 1H), 7.97-7.88 (m, 2H), 7.79 (d, *J* = 15.8 Hz, 1H), 7.61-7.56 (m, 3H), 7.35 (s, 1H), 7.0 (s, 2H), 6.80 (s, 1H), 3.99, (s, 3H), 3.95 (s, 3H), 2.82 (s, 3H); ^13^C NMR (CDCl_3_, 75 MHz) δ 161.5, 153.7, 152.3, 151.6, 151.2, 134.3, 133.9, 132.9, 129.4, 126.8, 125.4, 123.5, 121.5, 120.9, 119.9, 115.8, 113.1, 112.4, 109.4, 56.2, 55.9, 19.5; ESI- MS: (m/z); 372, found [M+H ]^+^ 373; HRMS-ESI: [M + H]^+^ for C_24_H_21_O_4_ calcd 373.1440, found 373.1433.

**(E)-4-(2,6-dimethoxystyryl)-7-methyl-2H-benzo[h]chromen-2-one (37):** Light yellow solid; mp 160-161 ^0^C, Anal.(%) for C_24_H_20_O_4_: Calcd., C, 77.40; H, 5.41; found, C, 77.31; H, 5.46; IR (KBr) 3410, 2358, 1700, 1588, 1388, 968 ν/cm^-1^, ^1^H NMR (300 MHz, CDCl_3_) δ 8.52 (d, *J* = 8.0 Hz, 1H), 8.02 (d, *J* = 16.1 Hz, 1H), 7.96-7.94 (m, 3H), 7.57-7.48 (m, 2H), 7.33 (d, *J* = 8.3 Hz, 1H), 6.75 (s, 1H), 6.65 (d, *J* = 8.4 Hz, 2H), 3.97, (s, 6H), 2.74 (s, 3H); ^13^C NMR (CDCl_3_, +DMSO-*d*_6_ 75 MHz) δ 166.3, 164.1, 158.1, 155.7, 139.0, 138.5, 135.4, 134.0, 133.6, 131.4, 128.3, 128.1, 125.3, 125.0, 118.8, 117.9, 112.8, 108.7, 60.7, 24.2; ESI- MS: (m/z); 372, found [M+H ]^+^ 373; HRMS-ESI: [M + H]^+^ for C_24_H_21_O_4_ calcd 373.1440, found 373.1430.

**(E)-7-methyl-4-(3,4,5-trimethoxystyryl)-2H-benzo[h]chromen-2-one (38):** Light yellow solid; mp 2015-206 ^0^C; Anal.(%) for C_25_H_22_O_5_: Calcd., C, 74.61; H, 5.51; found, C, 74.78; H, 5.48; IR (KBr) 3426, 2384, 1704, 1588, 1396, 968 ν/cm^-1^, ^1^H NMR (300 MHz, CDCl_3_) δ 8.52 (d, *J* = 8.0 Hz, 1H), 7.92-7.82 (m, 2H), 7.59-7.53 (m, 2H), 7.41-7.37 (m, 2H), 6.86 (s, 2H), 6.68 (s, 1H), 3.98, (s, 6H), 3.93 (s, 3H), 2.75 (s, 3H); ^13^C NMR (CDCl_3_, 75 MHz) δ 161.5, 153.7, 152.3, 151.6, 151.2,134.3, 133.9, 132.9, 129.4, 126.8, 125.4, 123.5, 121.5,120.9, 119.9,115.8, 113.1, 112.4, 109.4, 56.2, 55.9, 19.5; ESI- MS: (m/z); 402, found [M+H ]^+^ 403; HRMS-ESI: C_25_H_23_O_5_ [M + H]^+^ calcd 403.1545, found 403.1537.

**(E)-4-(3,4-dimethoxystyryl)-7-methyl-2H-benzo[h]chromen-2-one (39):** Light yellow solid; mp 210 ^0^C; Anal.(%) for C_24_H_20_O_4_: Calcd., C, 77.40; H, 5.41; found, C, 77.53; H, 5.39; IR, 3436, 2282, 1710, 1578, 1396, 958 (KBr) ν/cm^-1^, ^1^H NMR (300 MHz, CDCl_3_) δ 8.44 (d, *J* = 7.3 Hz, 1H), 7.84-7.76 (m, 2H), 7.48-7.44 (m, 2H), 7.20-7.10 (m, 4H), 6.88 (d, *J* = 8.3 Hz, 1H), 6.60 (s, 1H), 3.94, (s, 3H), 3.90 (s, 3H), 2.67 (s, 3H); ^13^C NMR (CDCl_3_, 75 MHz) δ 161.8, 151.4, 151.1, 150.6, 149.3, 137.8, 133.9, 129.5, 128.7, 126.9, 126.7, 123.4, 121.7, 120.9, 120.2, 119.6, 118.3, 113.6, 108.8, 108.2, 56.3, 55.7, 19.4; ESI- MS: (m/z); 372, found [M+H ]^+^ 373; HRMS-ESI: C_24_H_21_O_4_: [M + H]^+^ calcd 373.1440,found 373.1432.


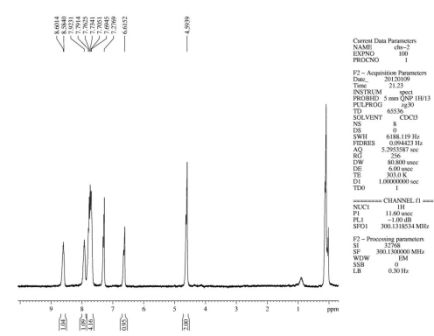


Supplementary figure 1: ^1^H NMR of compound 4


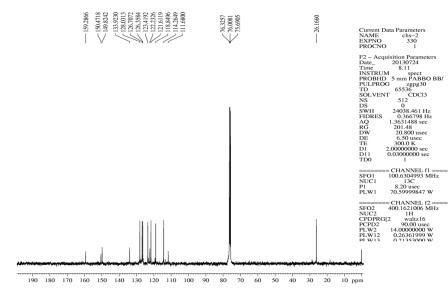


Supplementary figure 2 ^13^C NMR of compound 4


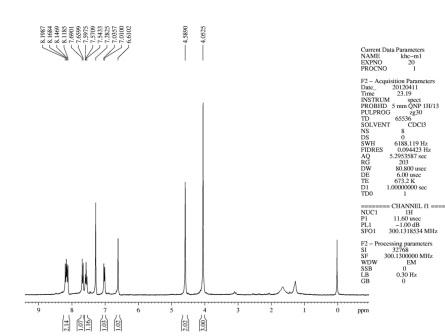


Supplementary figure 3 ^1^H NMR of compound 5


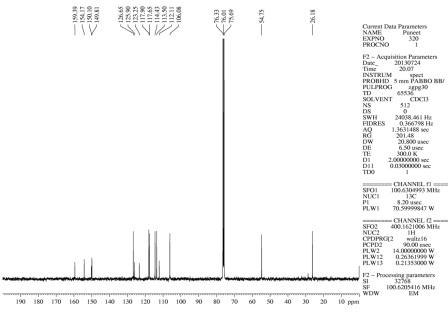


Supplementary figure 4 ^13^C NMR of compound 5


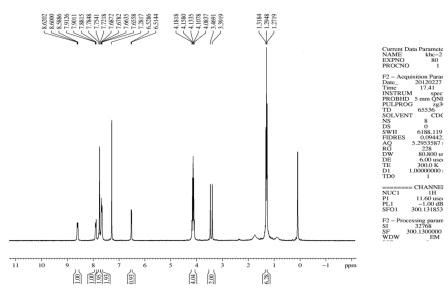


Supplementary figure 5 ^1^H NMR of compound 7


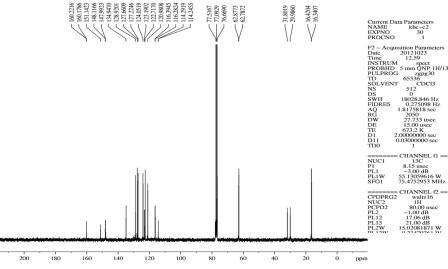


Supplementary figure 6 ^13^C NMR of compound 7


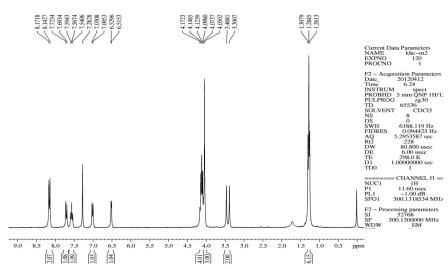


Supplementary figure 7 ^1^H NMR of compound 8


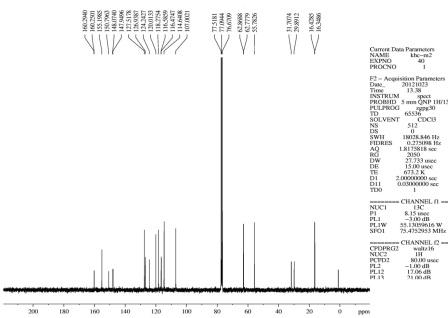


Supplementary figure 8 ^13^C NMR of compound 8


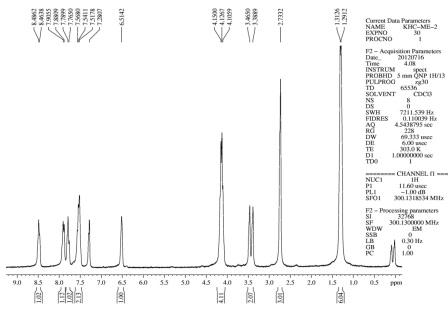


Supplementary figure 9 ^1^H NMR of compound 9


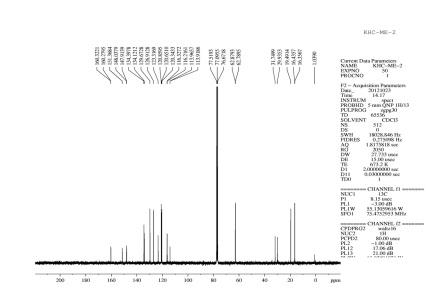


Supplementary figure 10 ^13^C NMR of compound 9


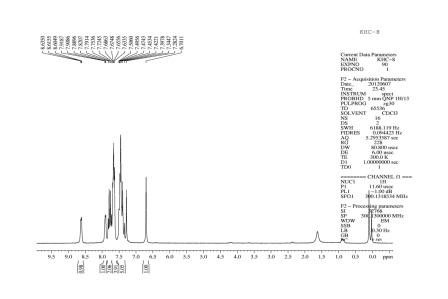


Supplementary figure 11 ^1^H NMR of compound 10


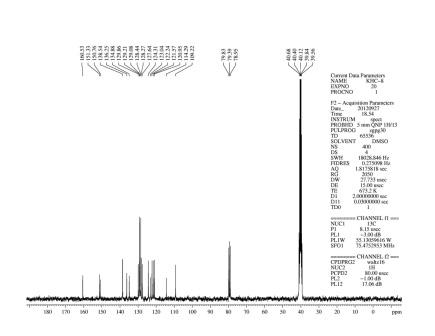


Supplementary figure 12 ^13^C NMR (CDCl_3_, + DMSO-*d*_6_) of compound 10


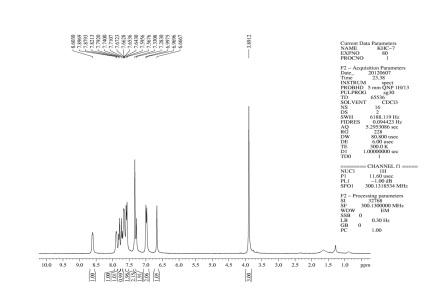


Supplementary figure 13 ^1^H NMR of compound 11


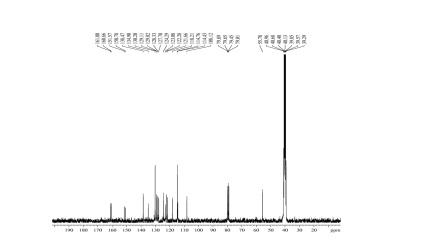


Supplementary figure 14 ^13^C NMR of compound 11


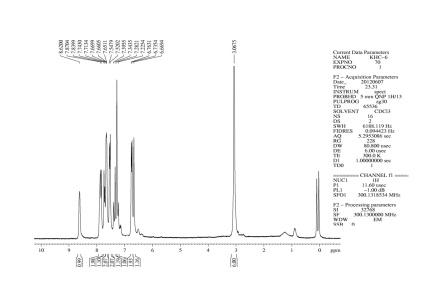


Supplementary figure 15 ^1^H NMR of compound 12


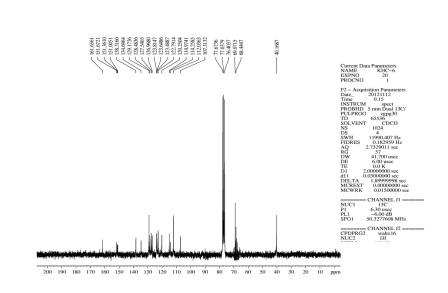


Supplementary figure 16 ^13^C NMR of compound 12


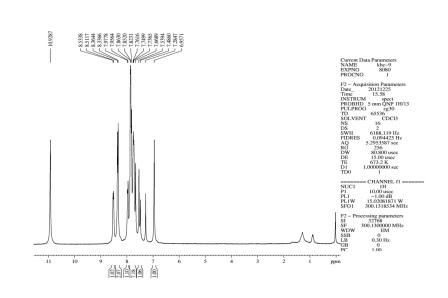


Supplementary figure 17 ^1^H NMR, ( CDCl_3_ +TFA-*d*) of compound 13


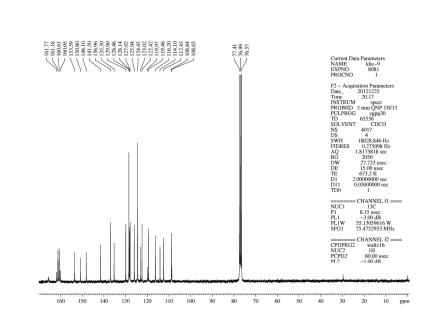


Supplementary figure 18 ^13^C NMR of compound 13


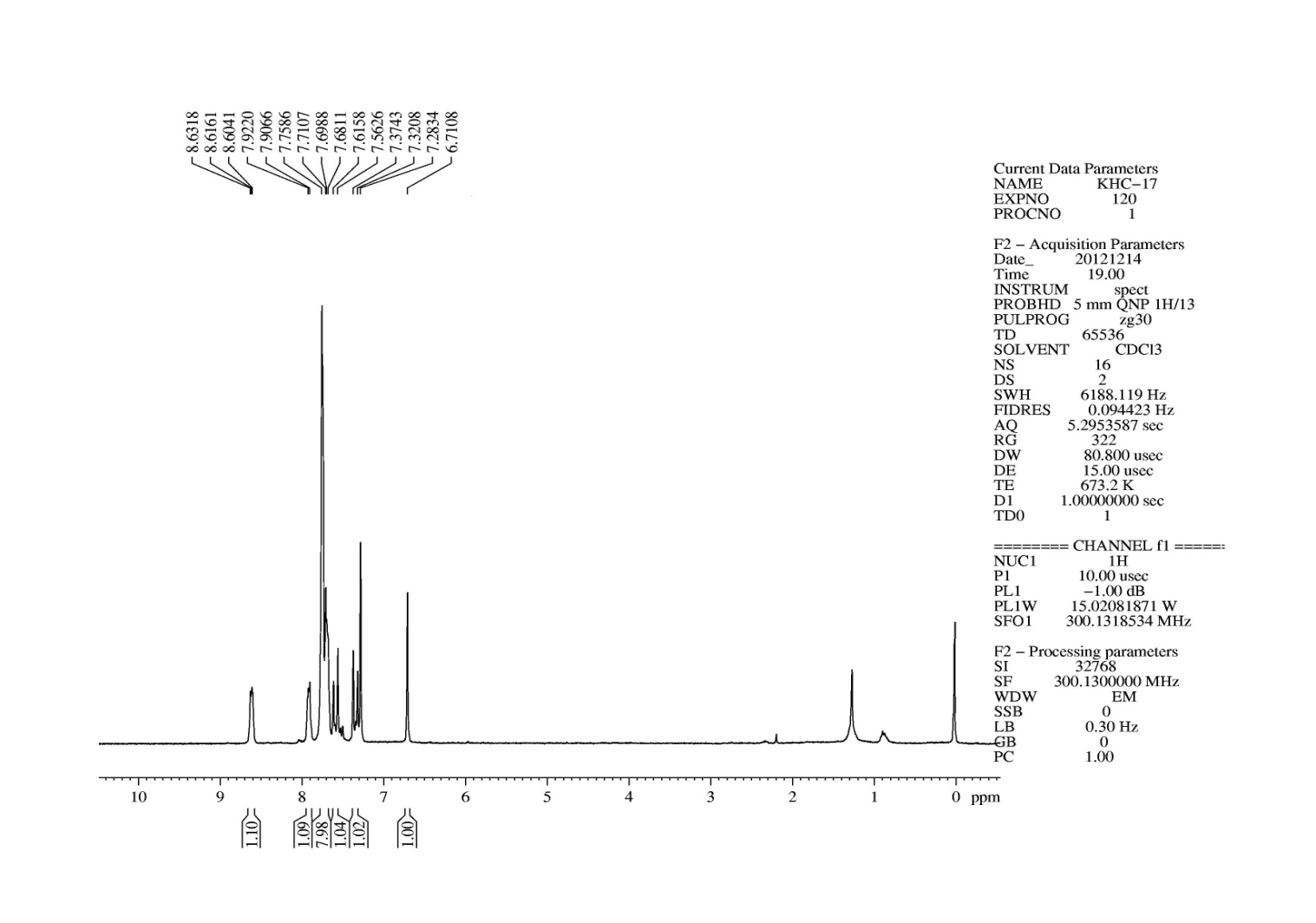


Supplementary figure 19 ^1^H NMR of compound 14


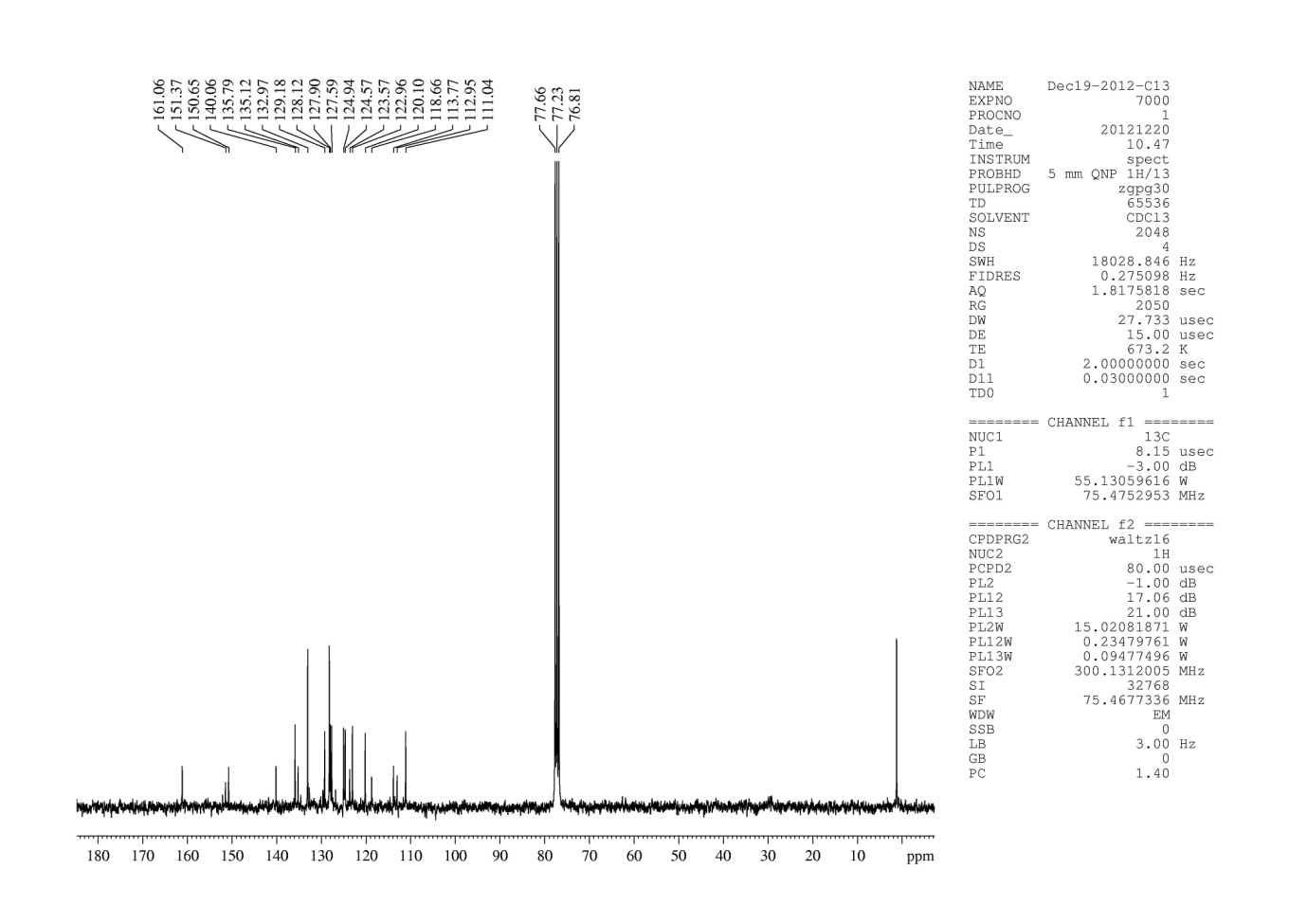


Supplementary fgure 20 ^13^C NMR of compound 14


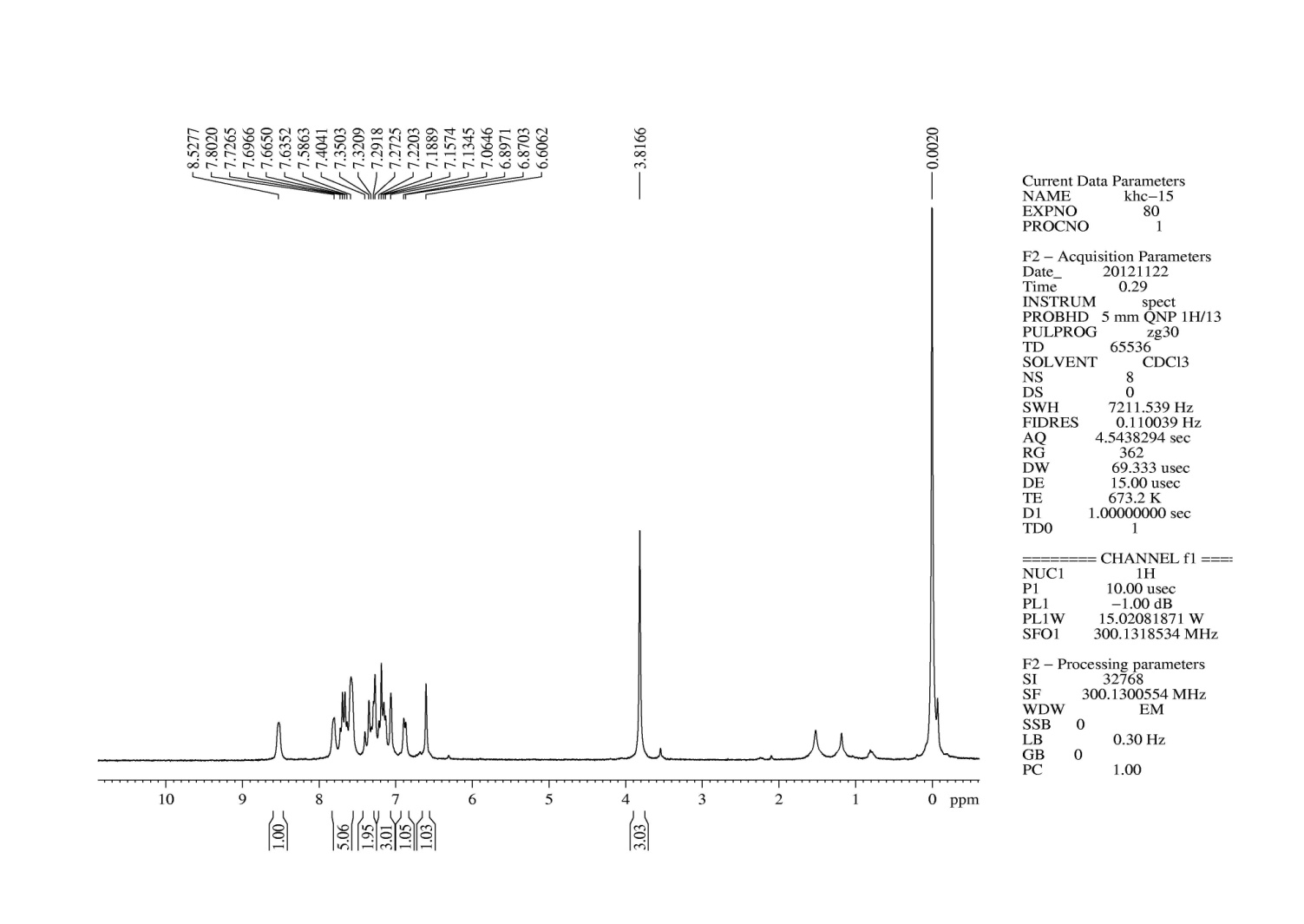


Supplementary figure 21 ^1^H NMR of compound 15


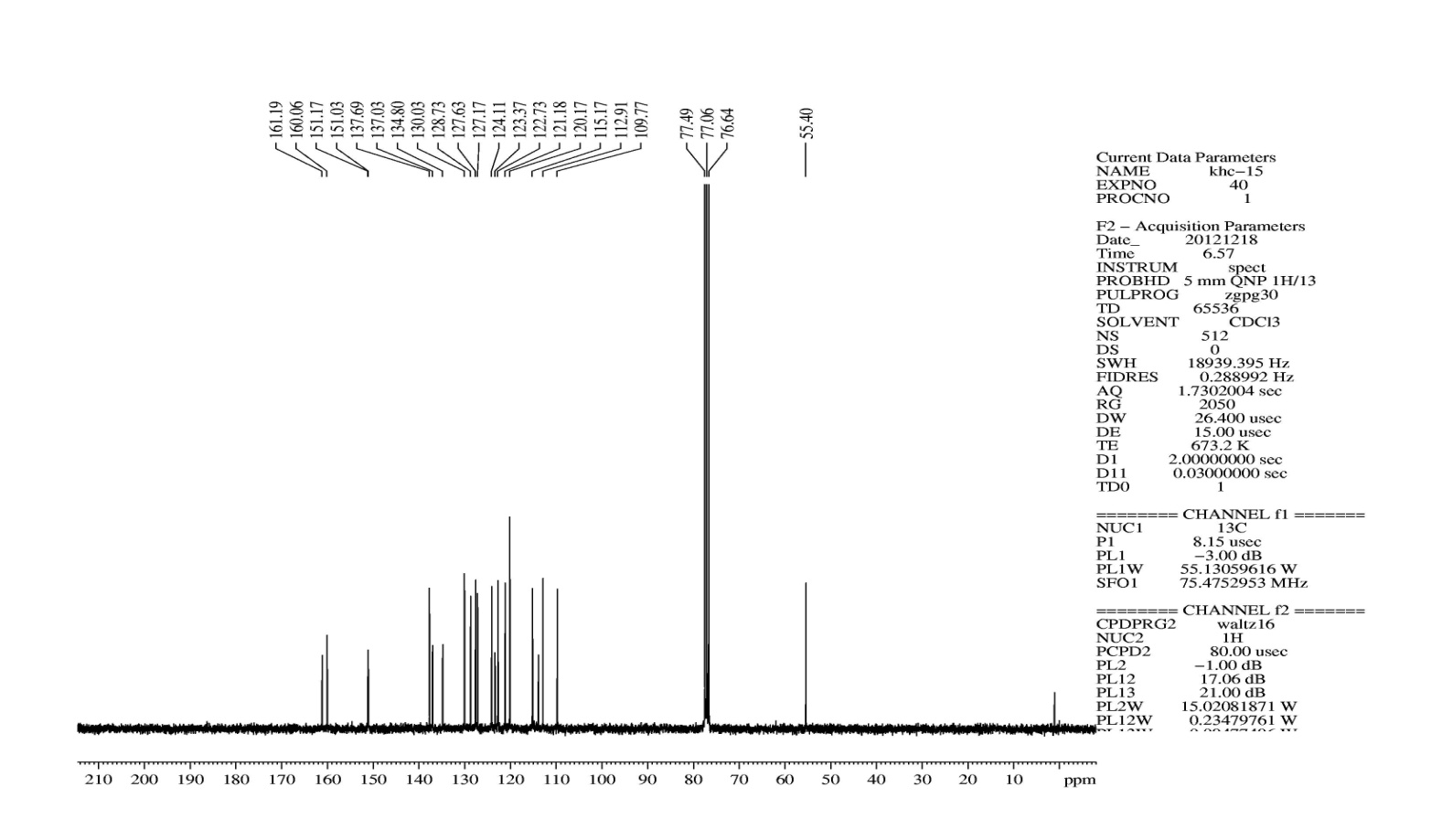


Supplementary figure 22 ^13^C NMR of compound 15


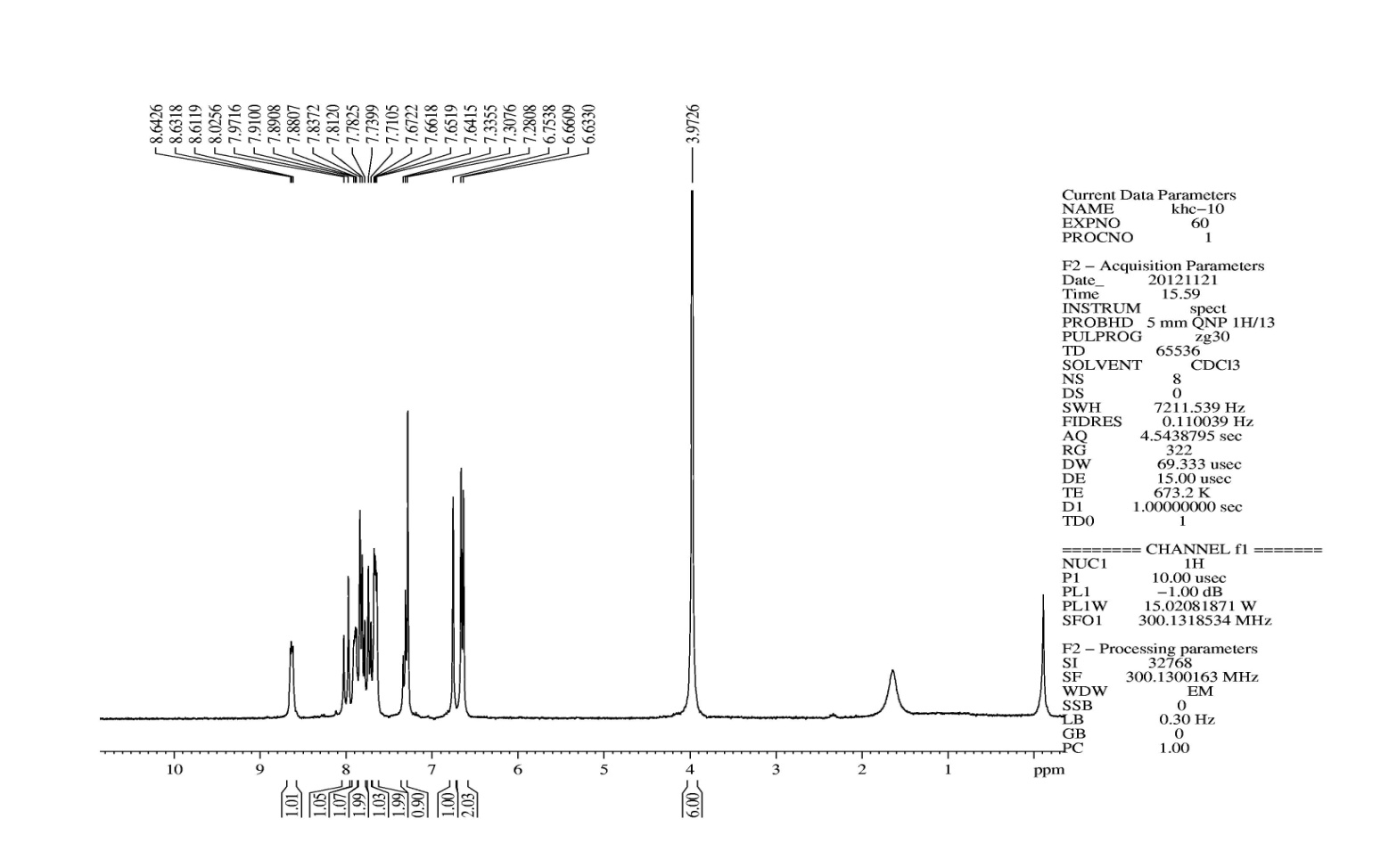


Supplementary figure 23 ^1^H NMR (DMSO-*d_6_* ) of compound 16


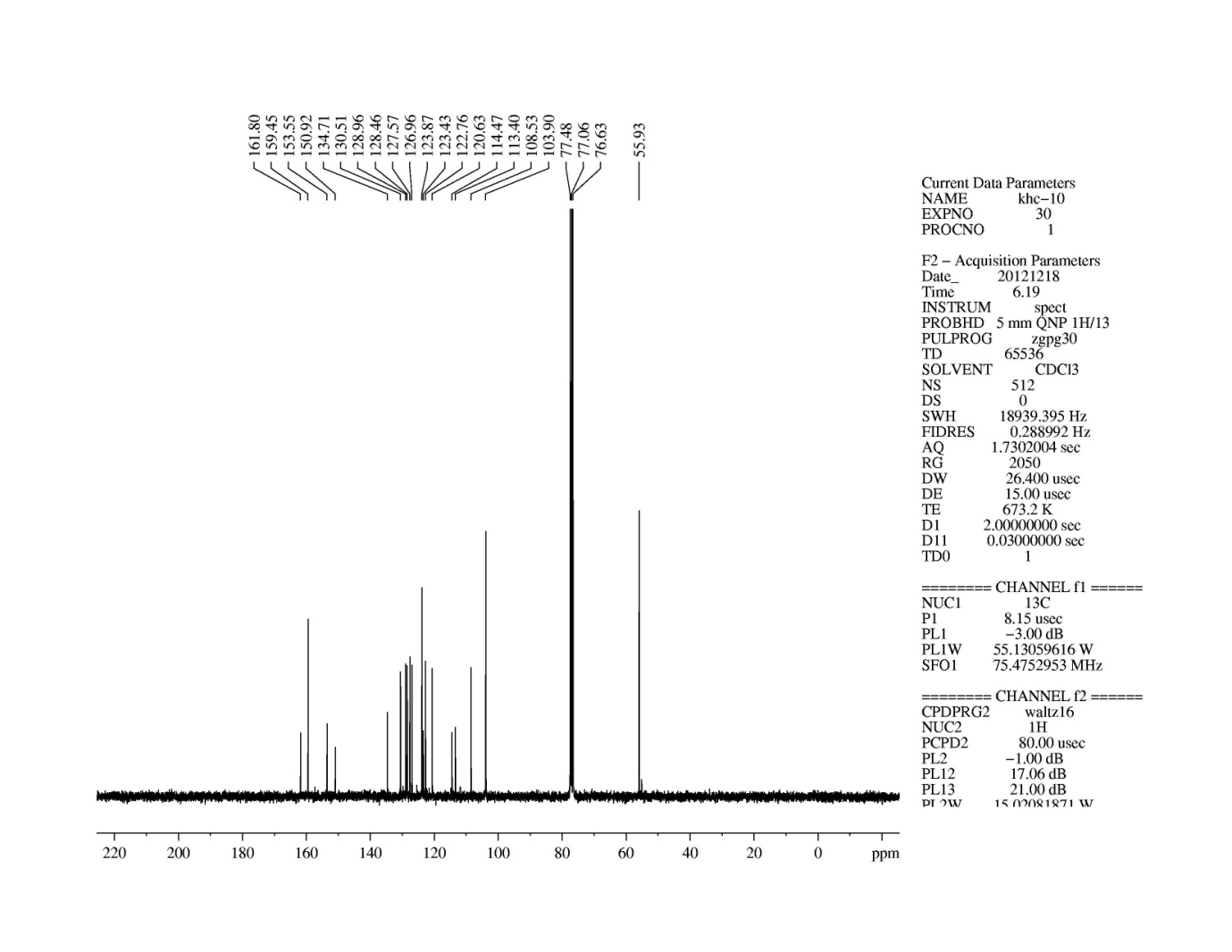


Supplementary figure 24 ^13^C NMR of compound 16


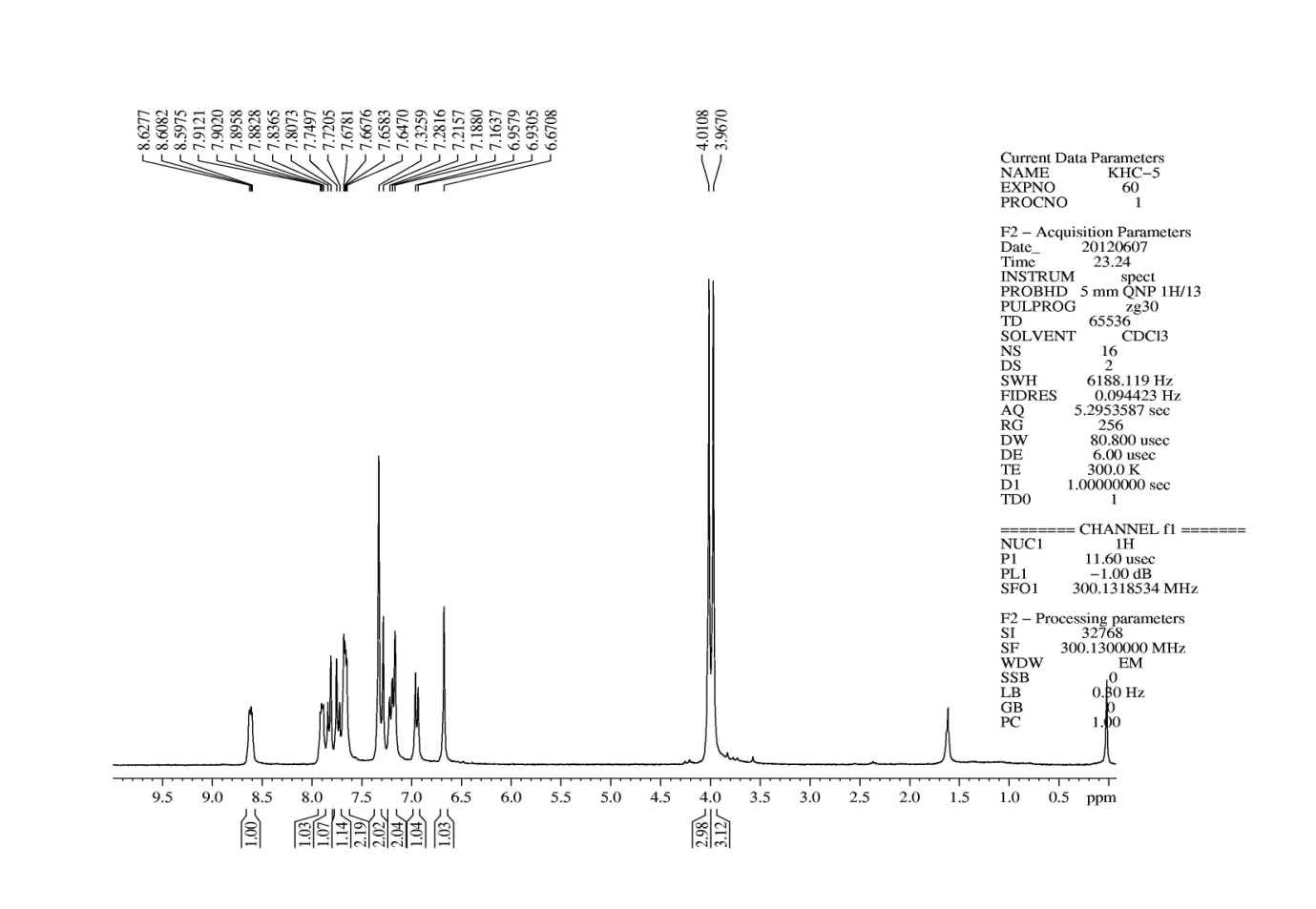


Supplementary figure 25 ^1^H NMR of compound 17


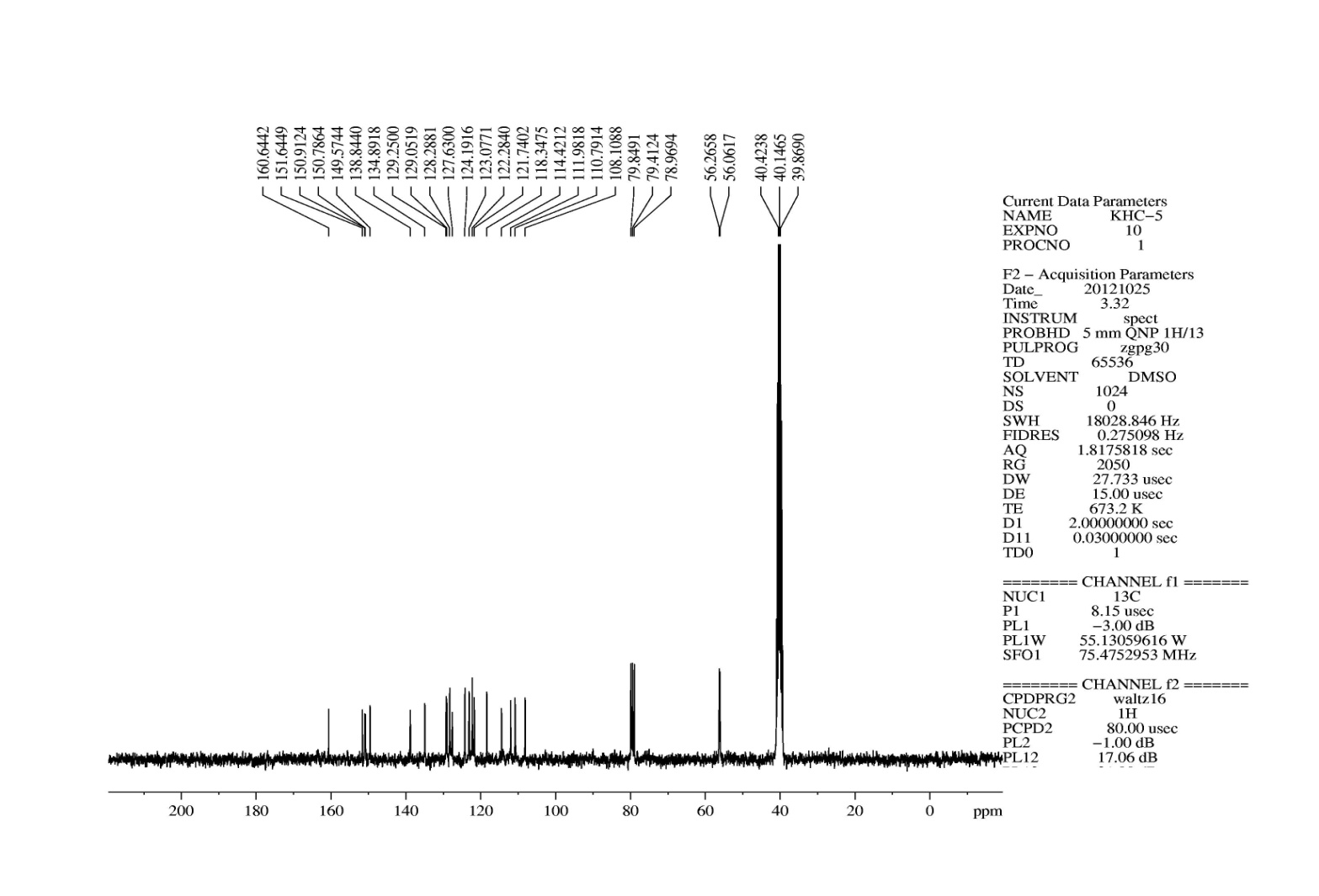


Supplementary figure 26 ^13^C NMR of compound 17


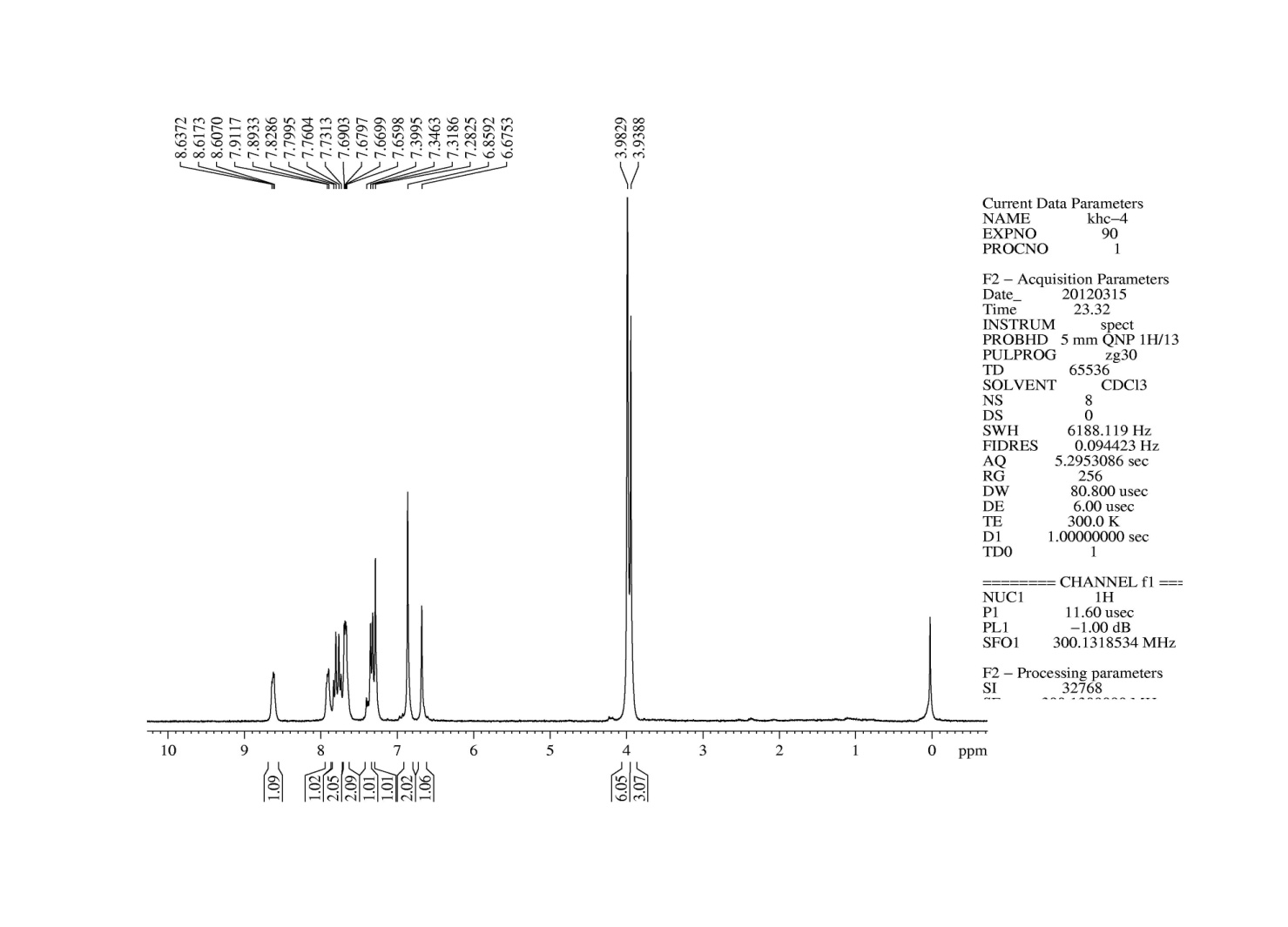


Supplementary figure 27 ^1^H NMR of compound 18


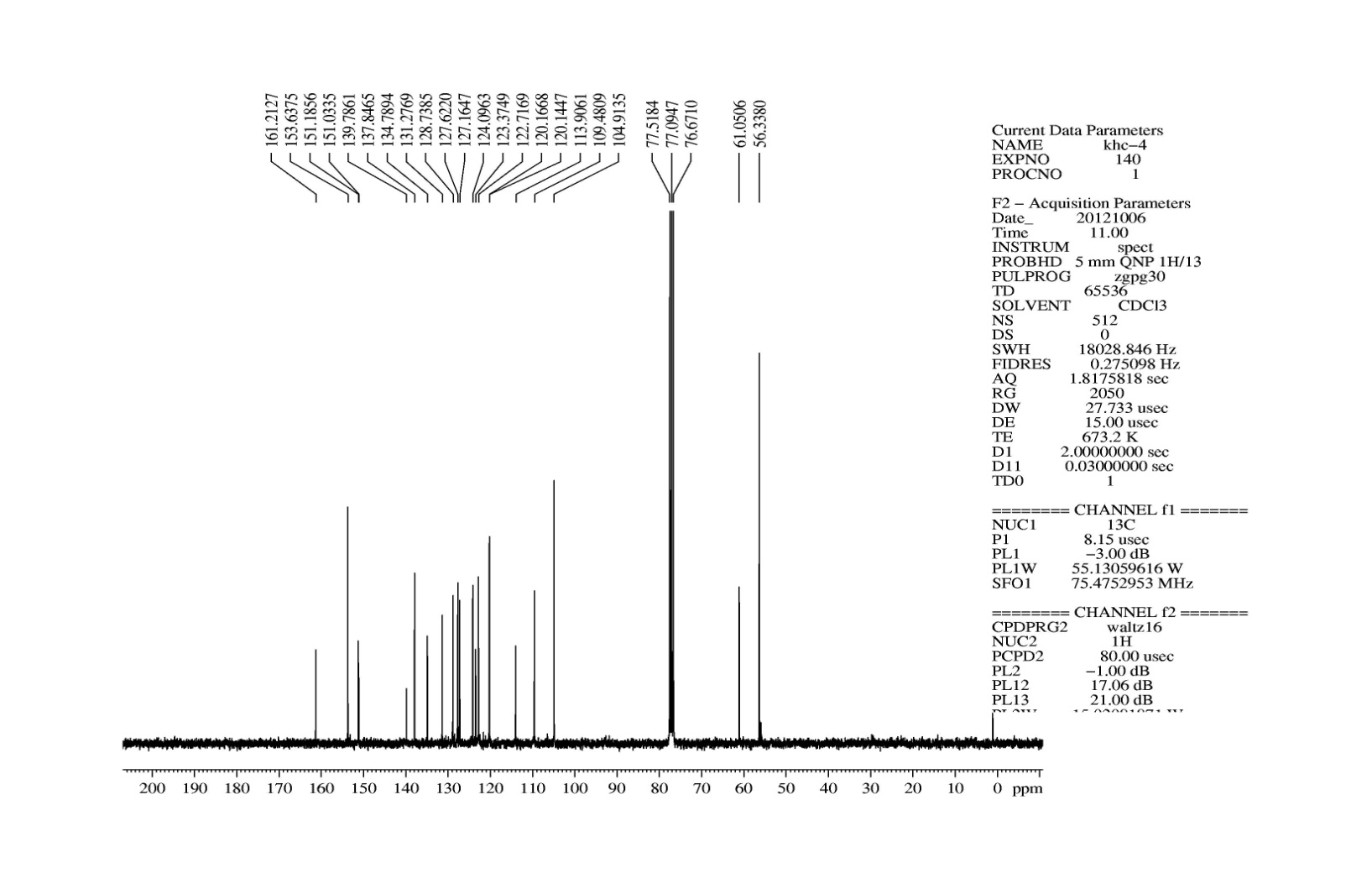


Supplementary figure 28 ^13^C NMR of compound 18


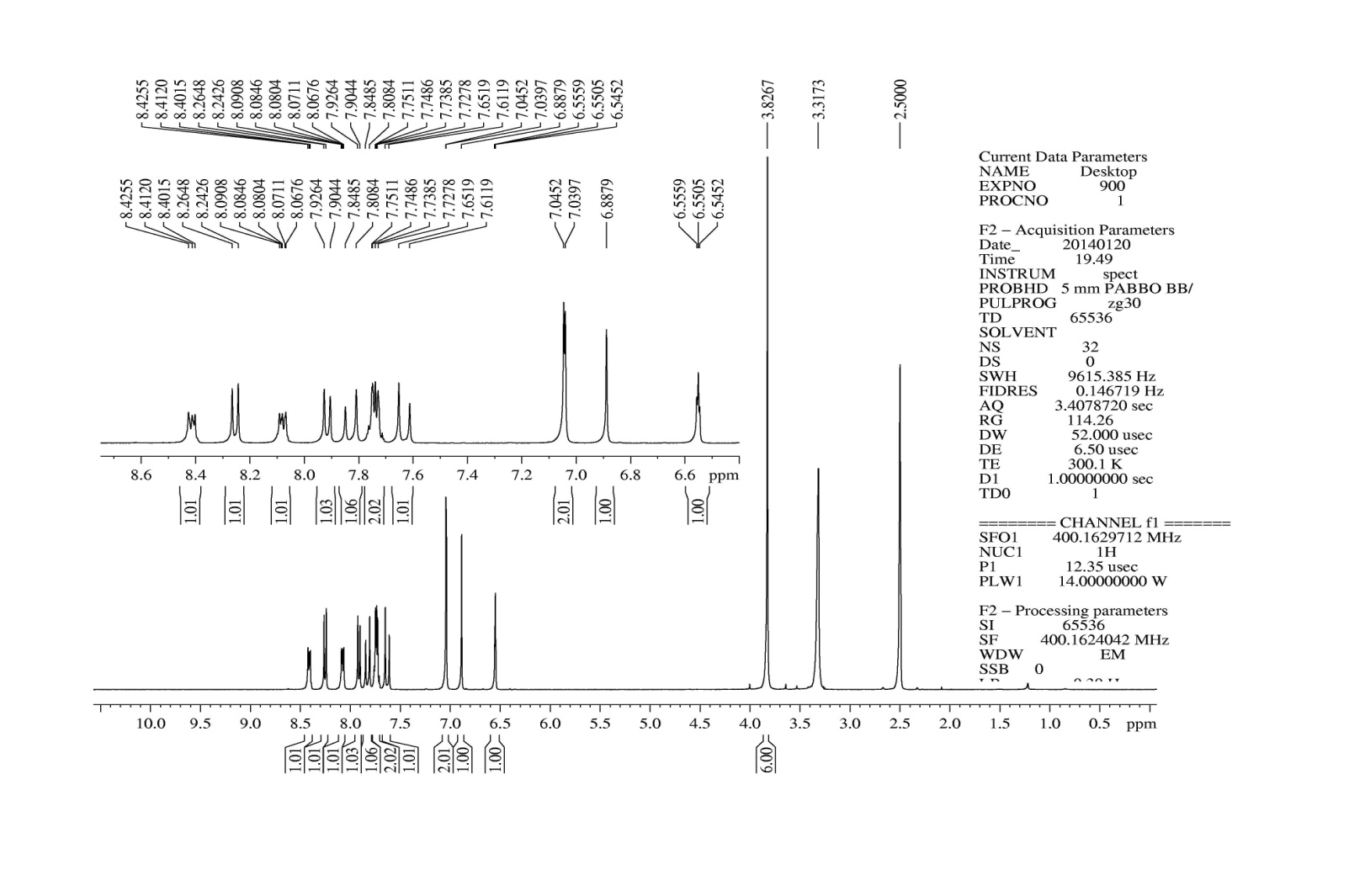


**Supplementary figure 29 ^1^H NMR (**DMSO-*d_6_***) of compound 19**


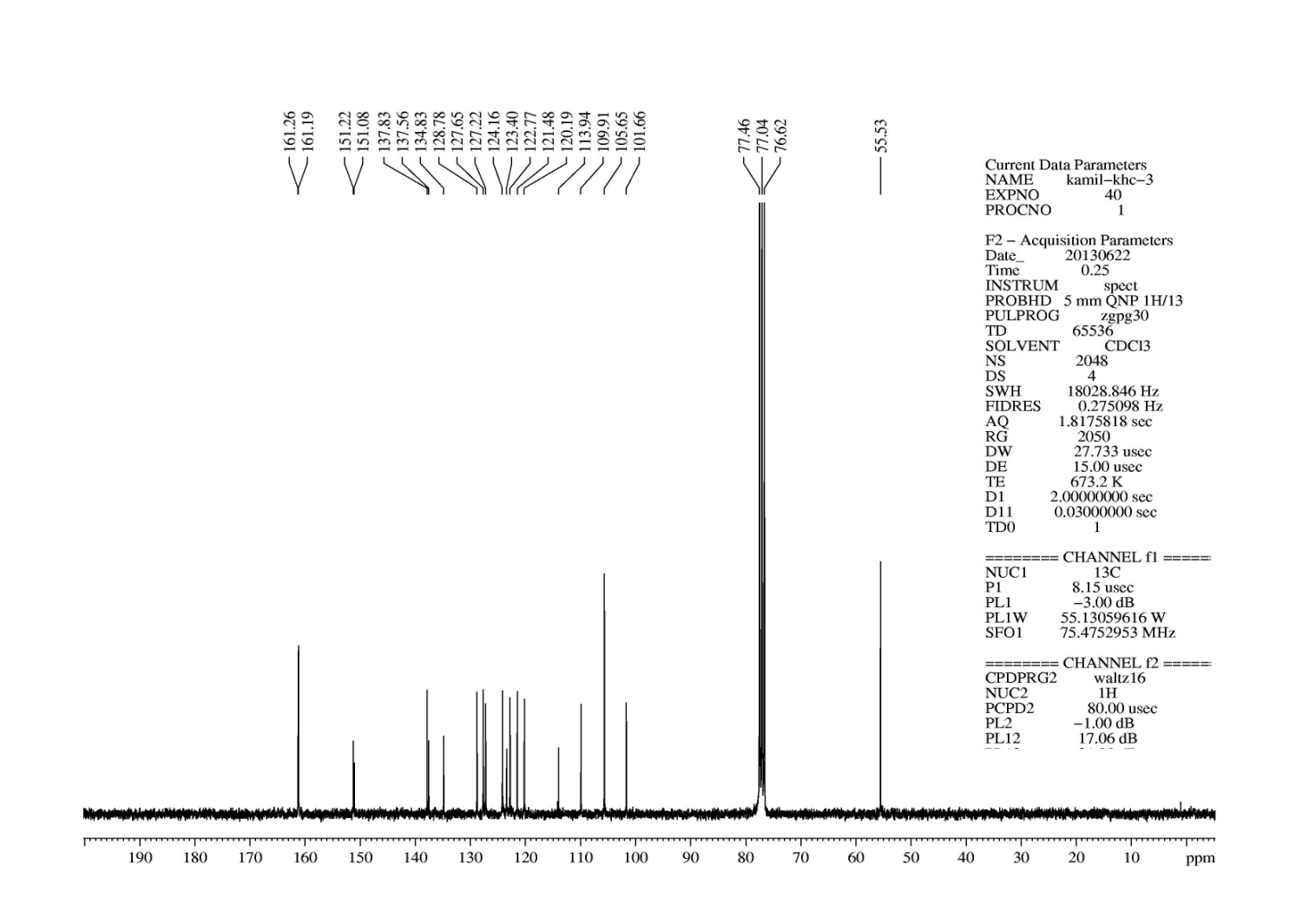
 Supplementary figure 30 ^13^C NMR of compound 19


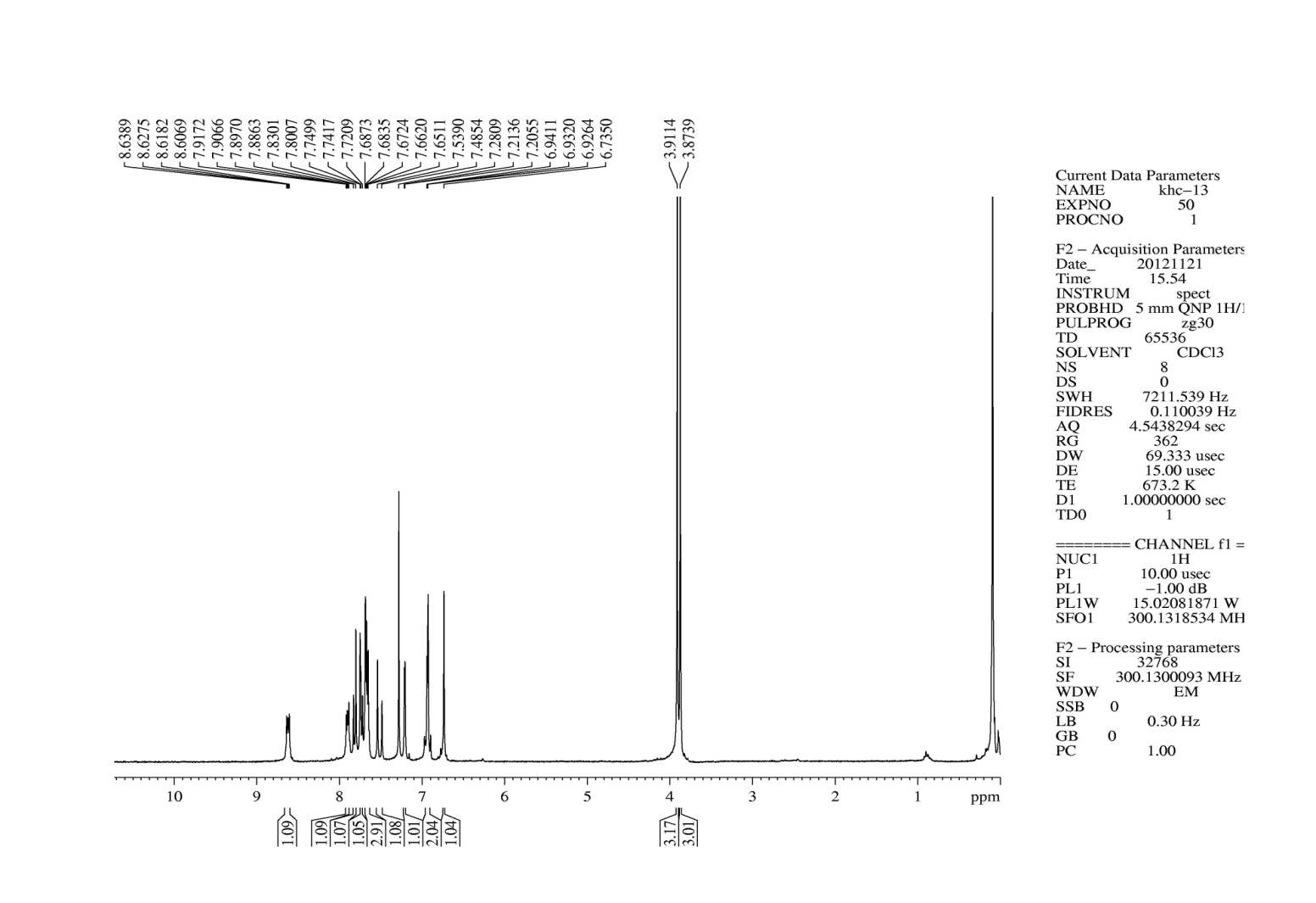


Supplementary figure 31 ^1^H NMR of compound 20


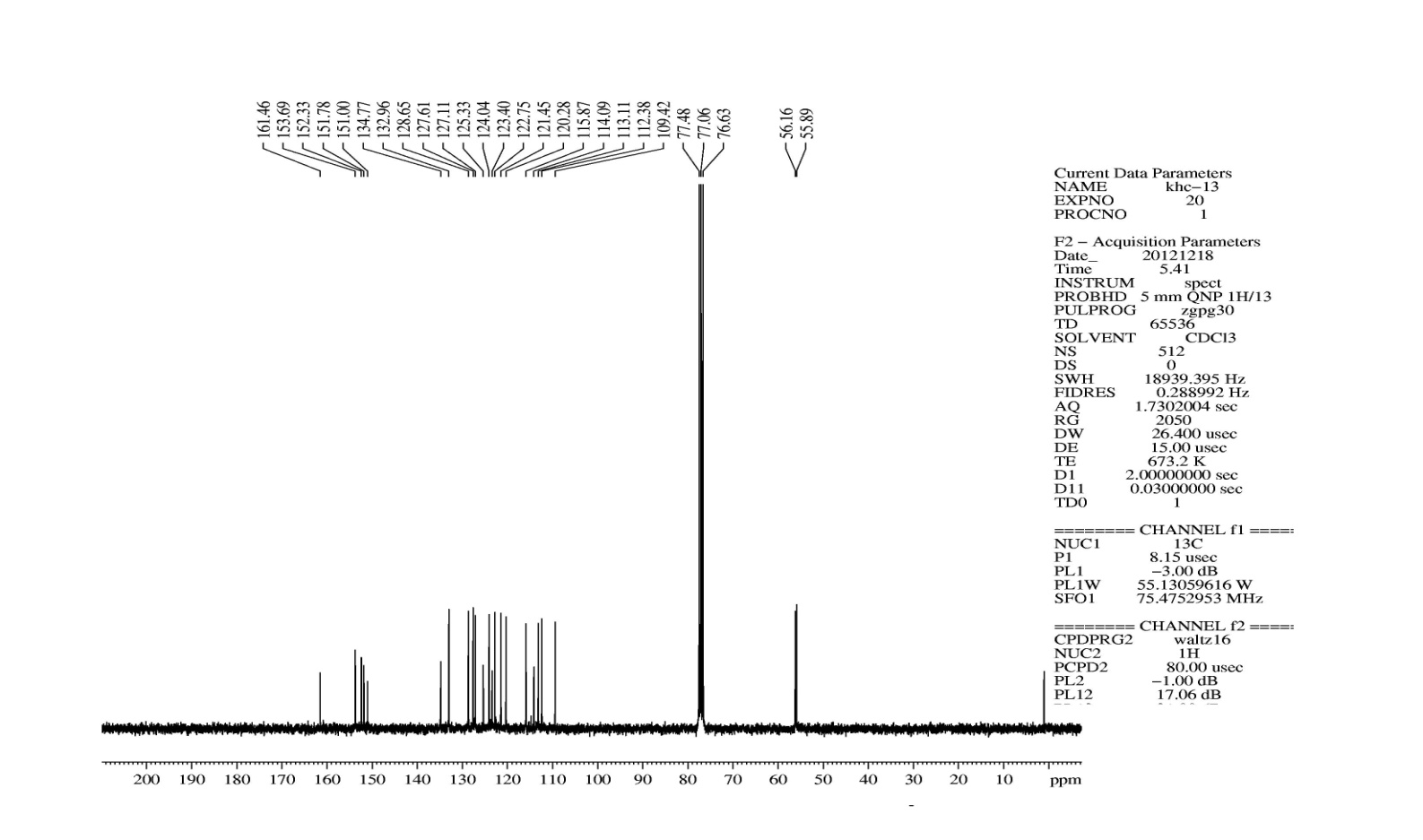


Supplementary figure 32 ^13^C NMR of compound 20


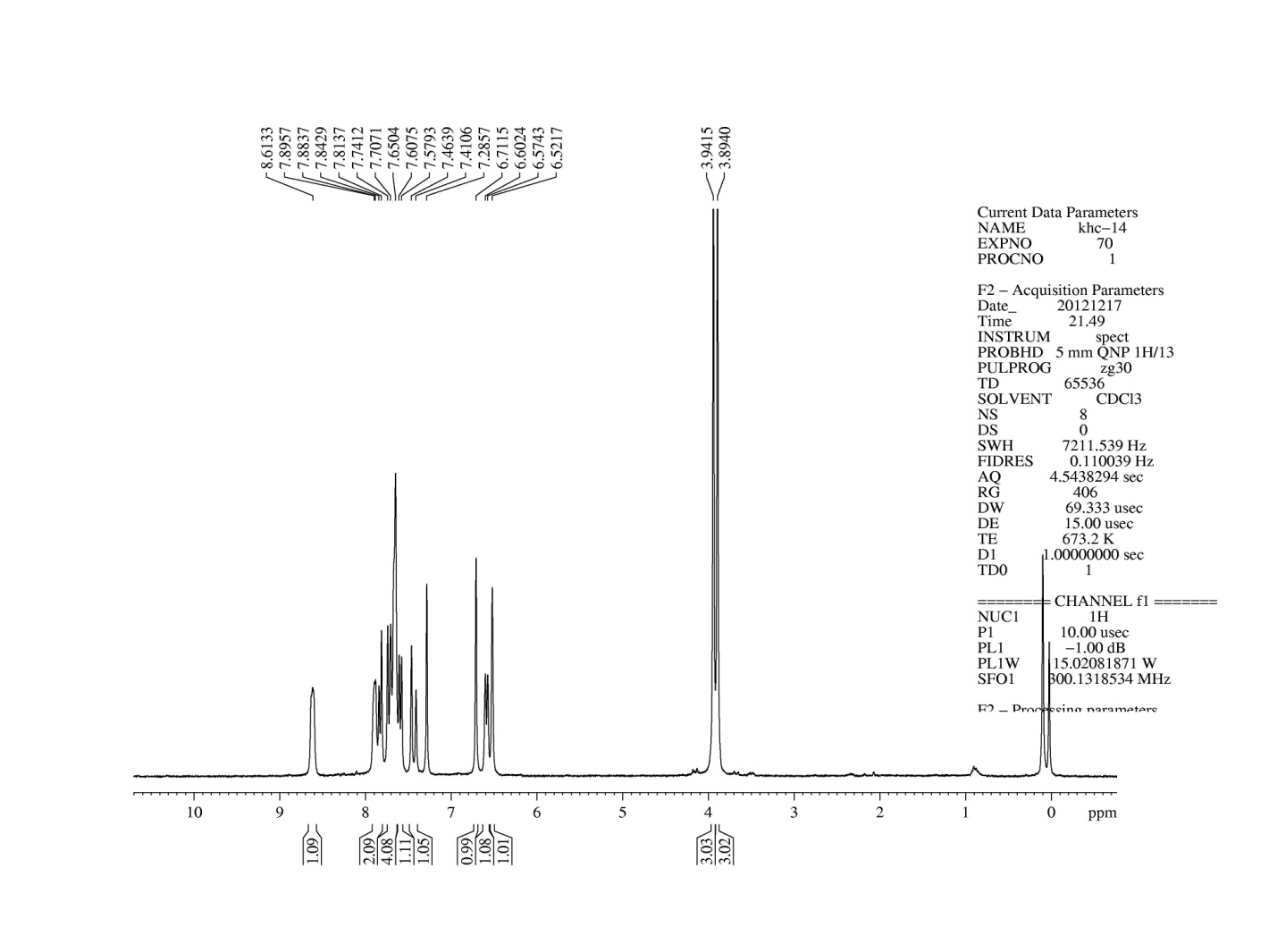


Supplementary figure 33 ^1^H NMR of compound 21


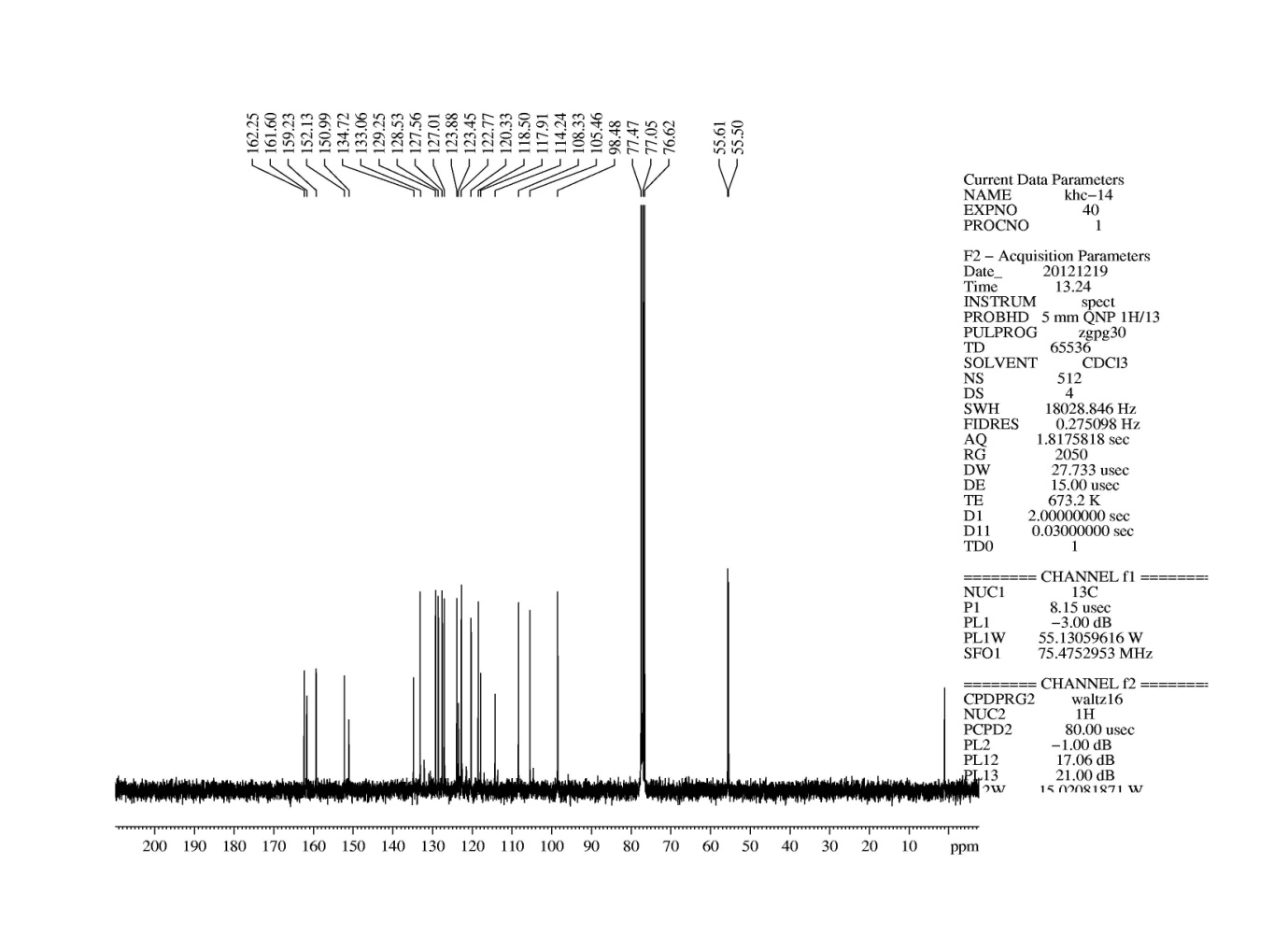


Supplementary figure 34 ^13^C NMR of compound 21


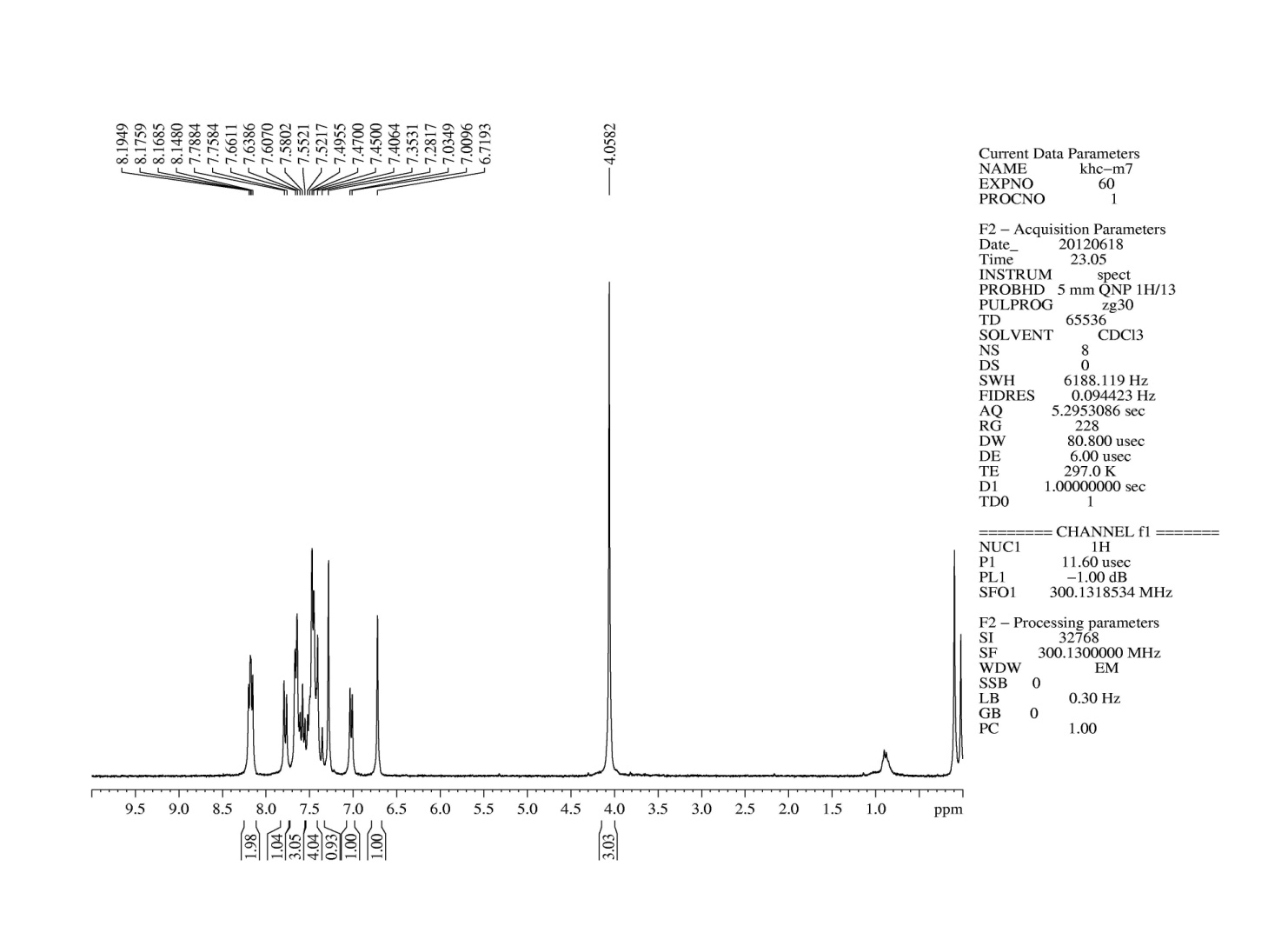


Supplementary figure 35 ^1^H NMR of compound 22


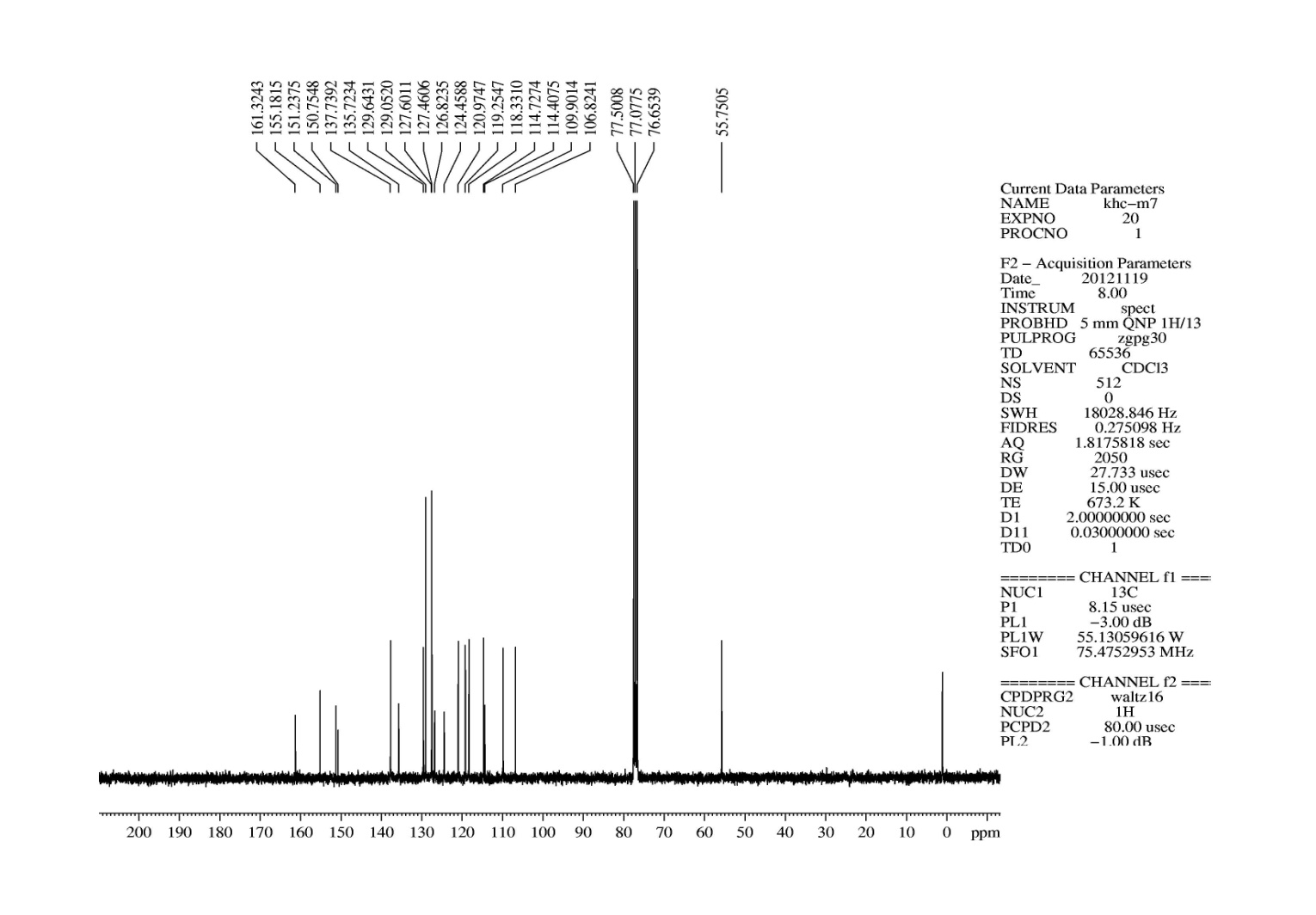


Supplementary figure 36 ^13^C NMR of compound 22


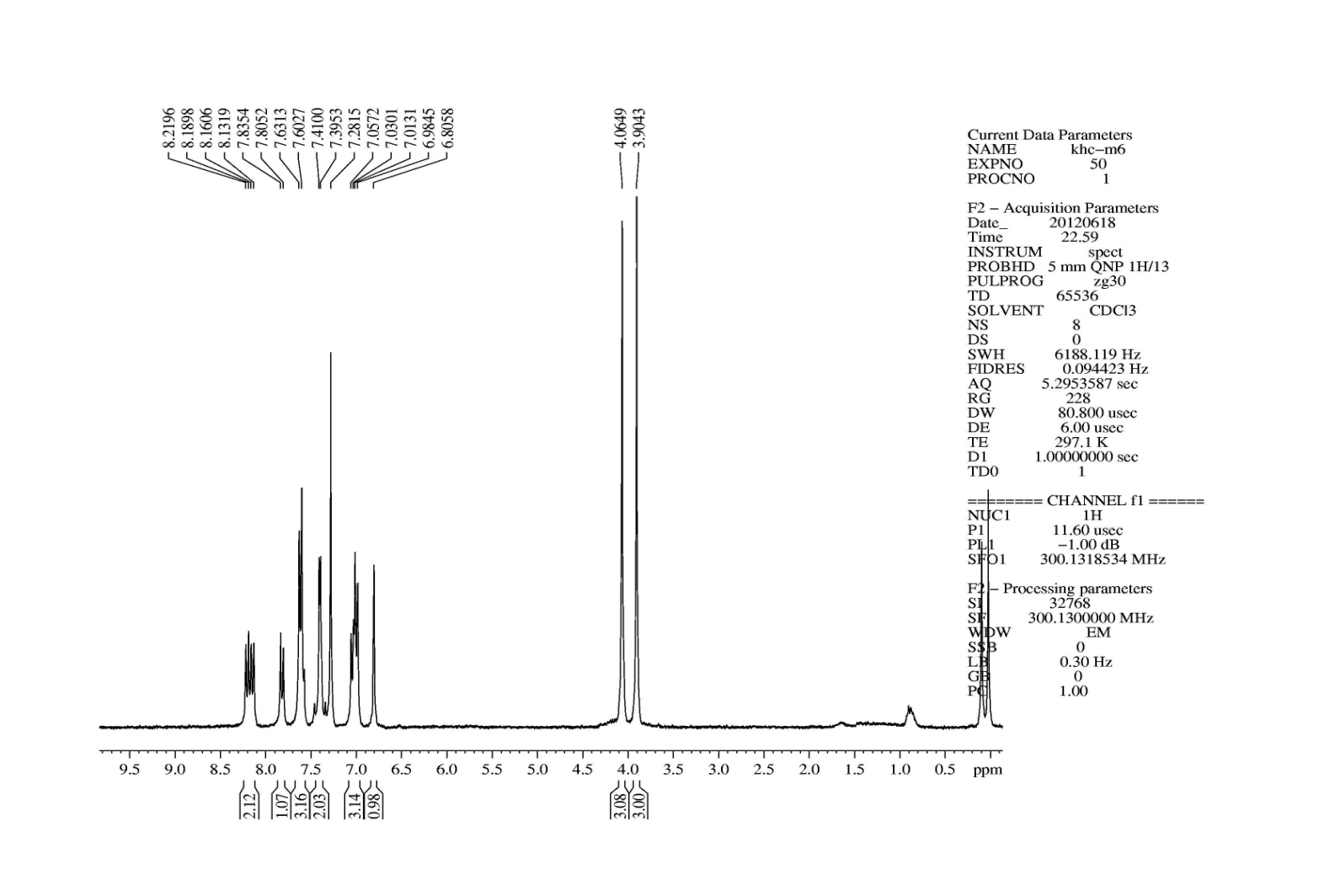


Supplementary figure 37 ^1^H NMR of compound 23


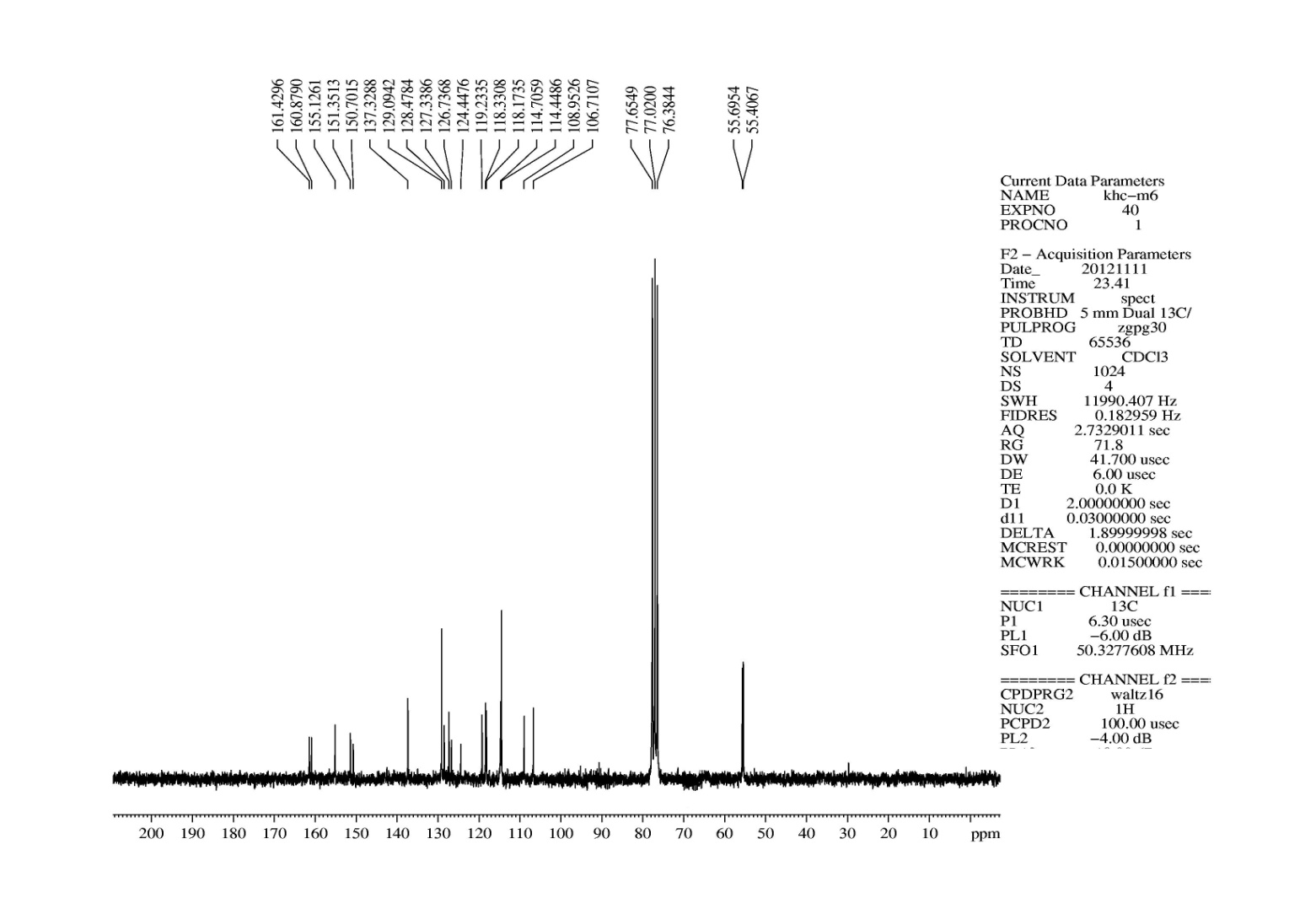


Supplementary figure 38 ^13^C NMR of compound 23


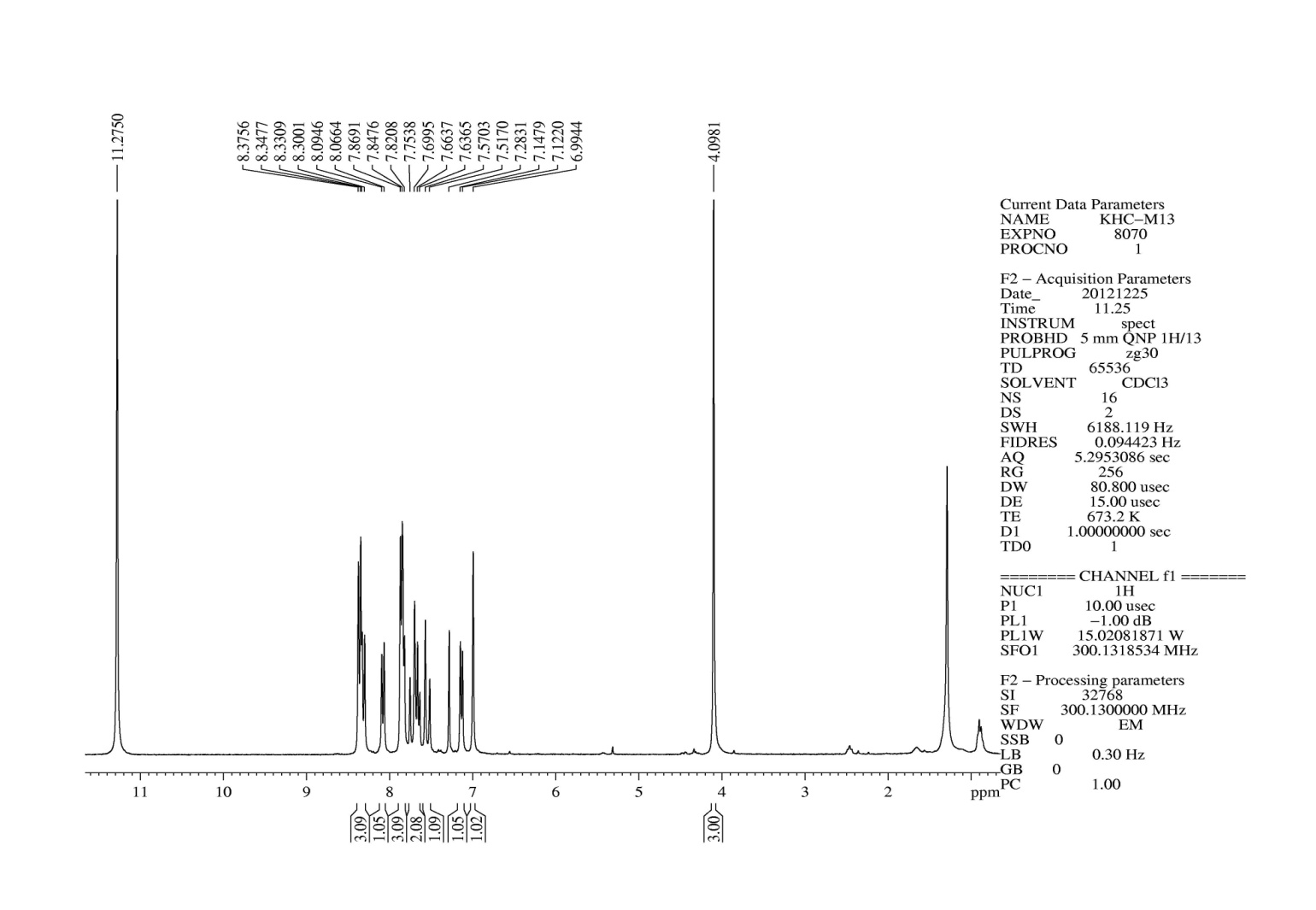


Supplementary figure 39 ^1^H NMR of compound 24 (CDCl_3_ + TFA-*d*_1_)


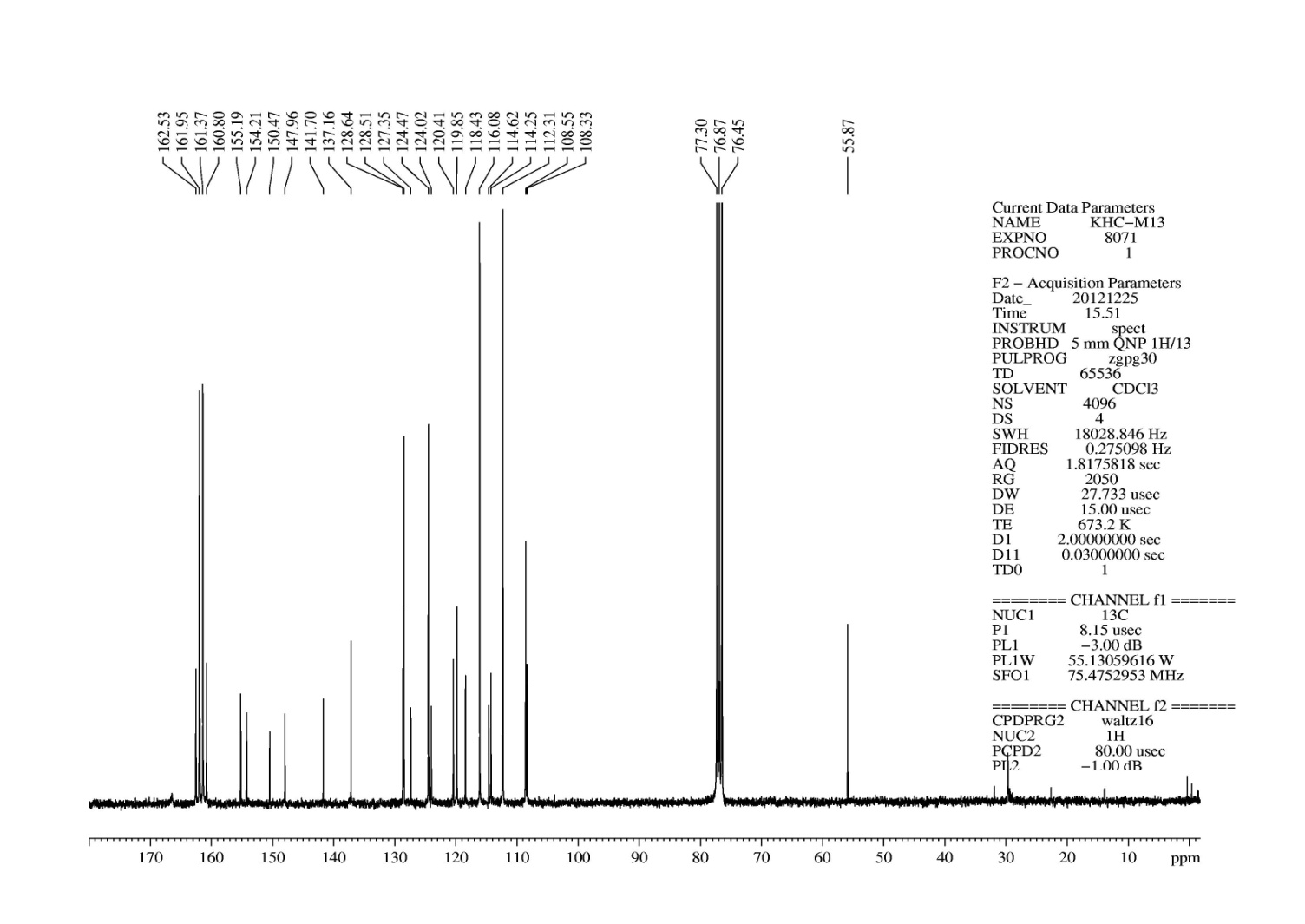


Supplementary figure 40 ^13^C NMR of compound 24 (CDCl_3_ + TFA-*d1*)


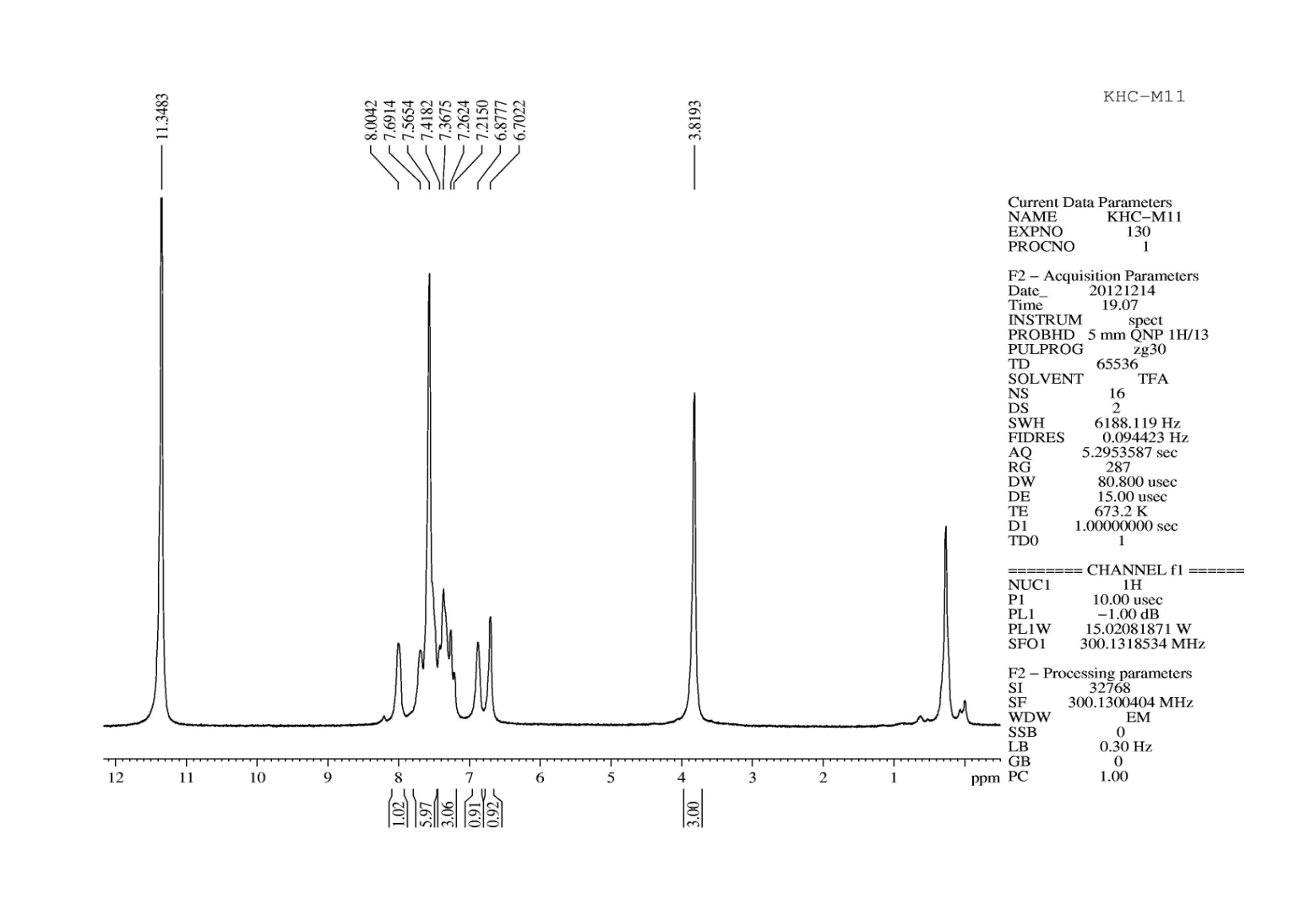


Supplementary figure 41 ^1^H NMR of compound 25 (TFA-d1)


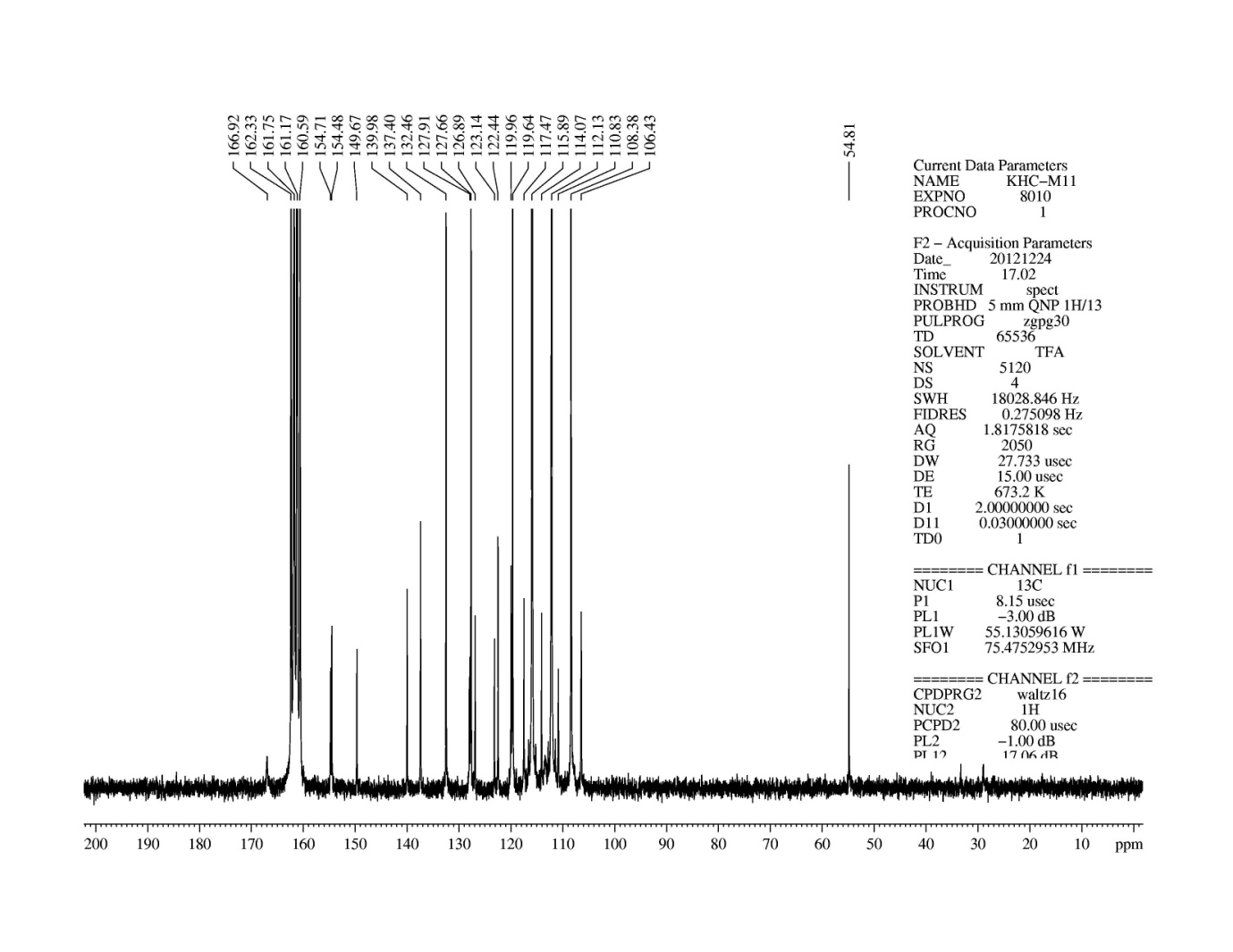


Supplementary figure 42 ^13^C NMR of compound 25 (TFA-d1)


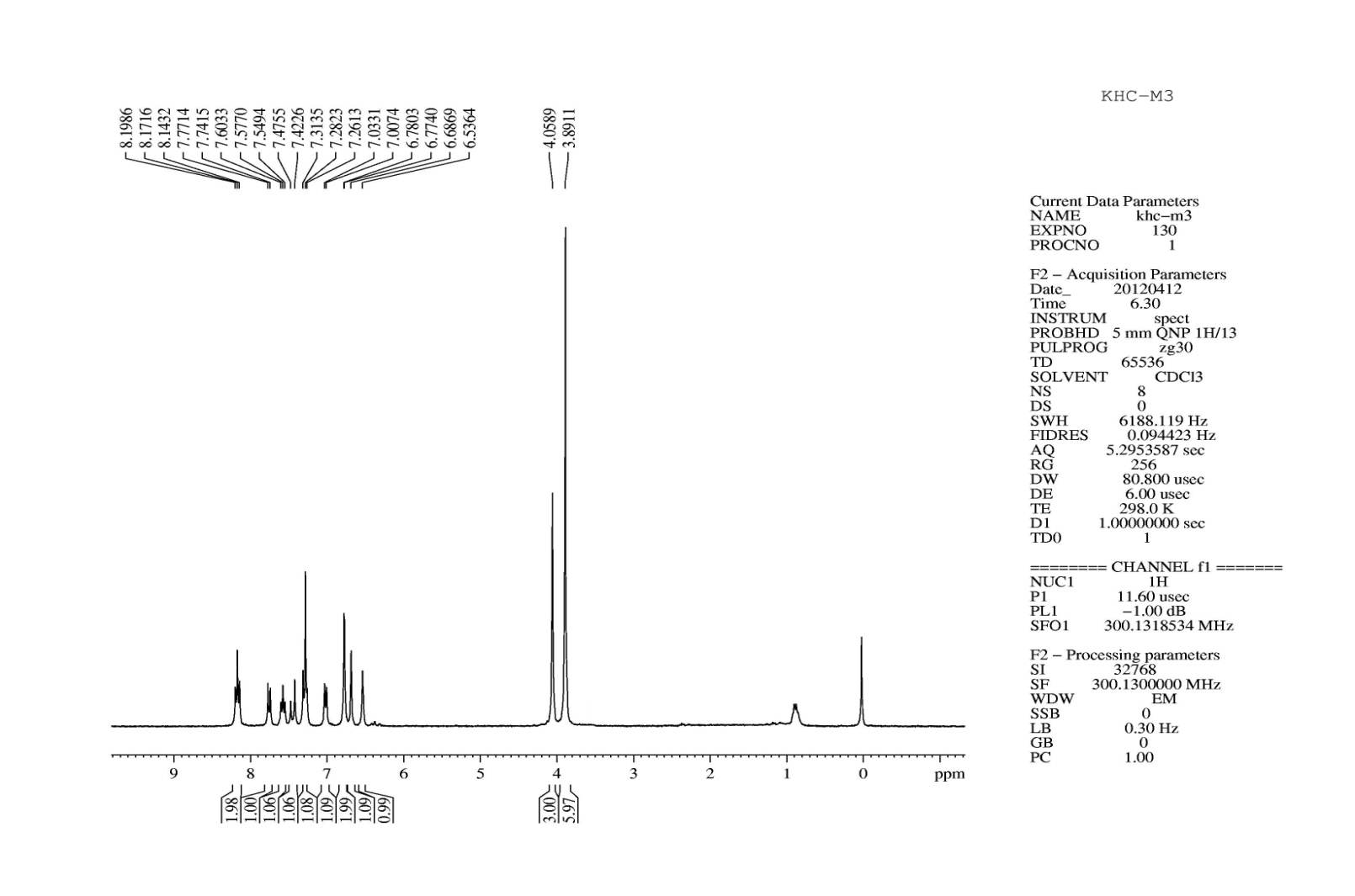


Supplementary figure 43 ^1^H NMR of compound 26


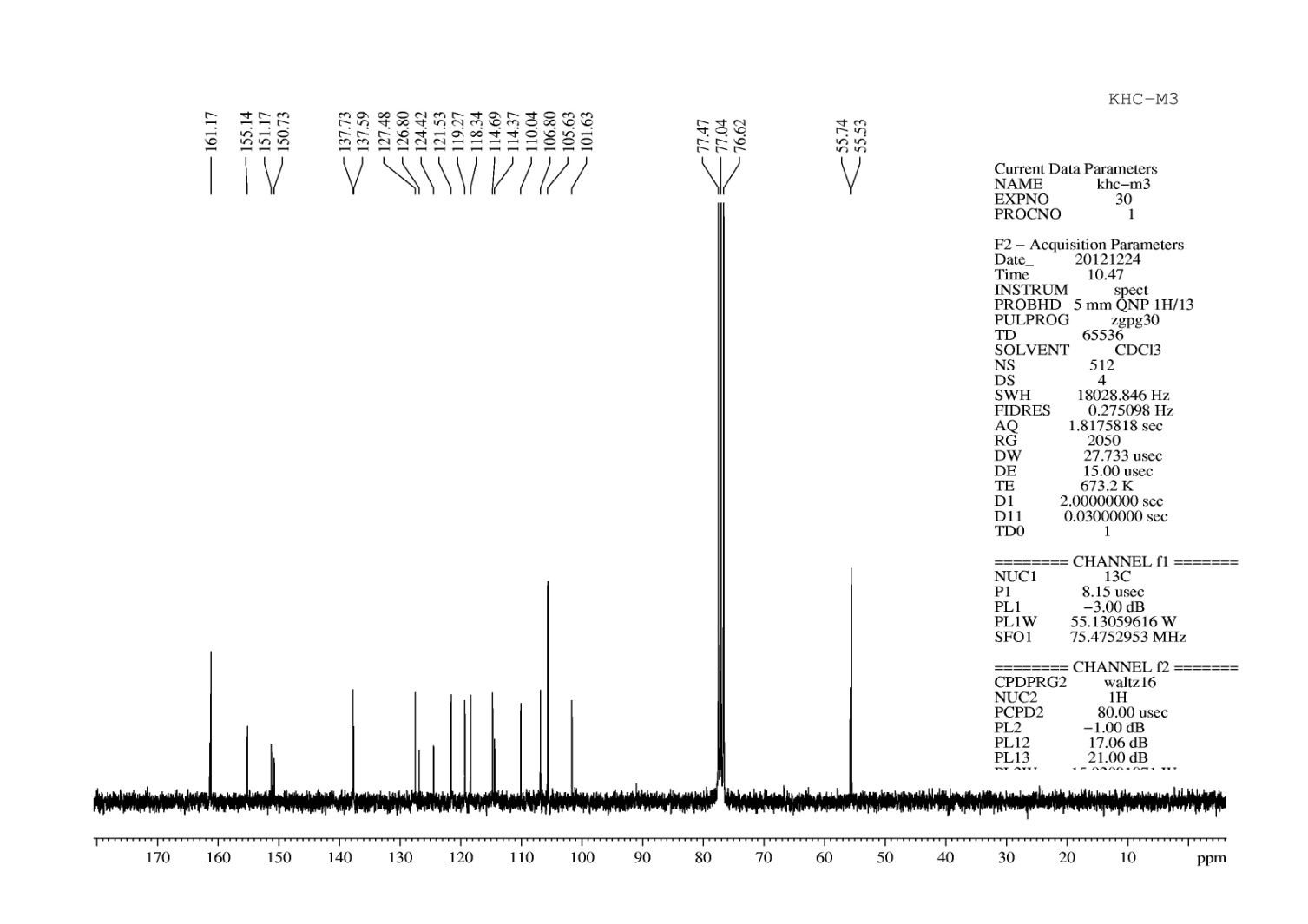


Supplementary figure 44 ^13^C NMR of compound 26


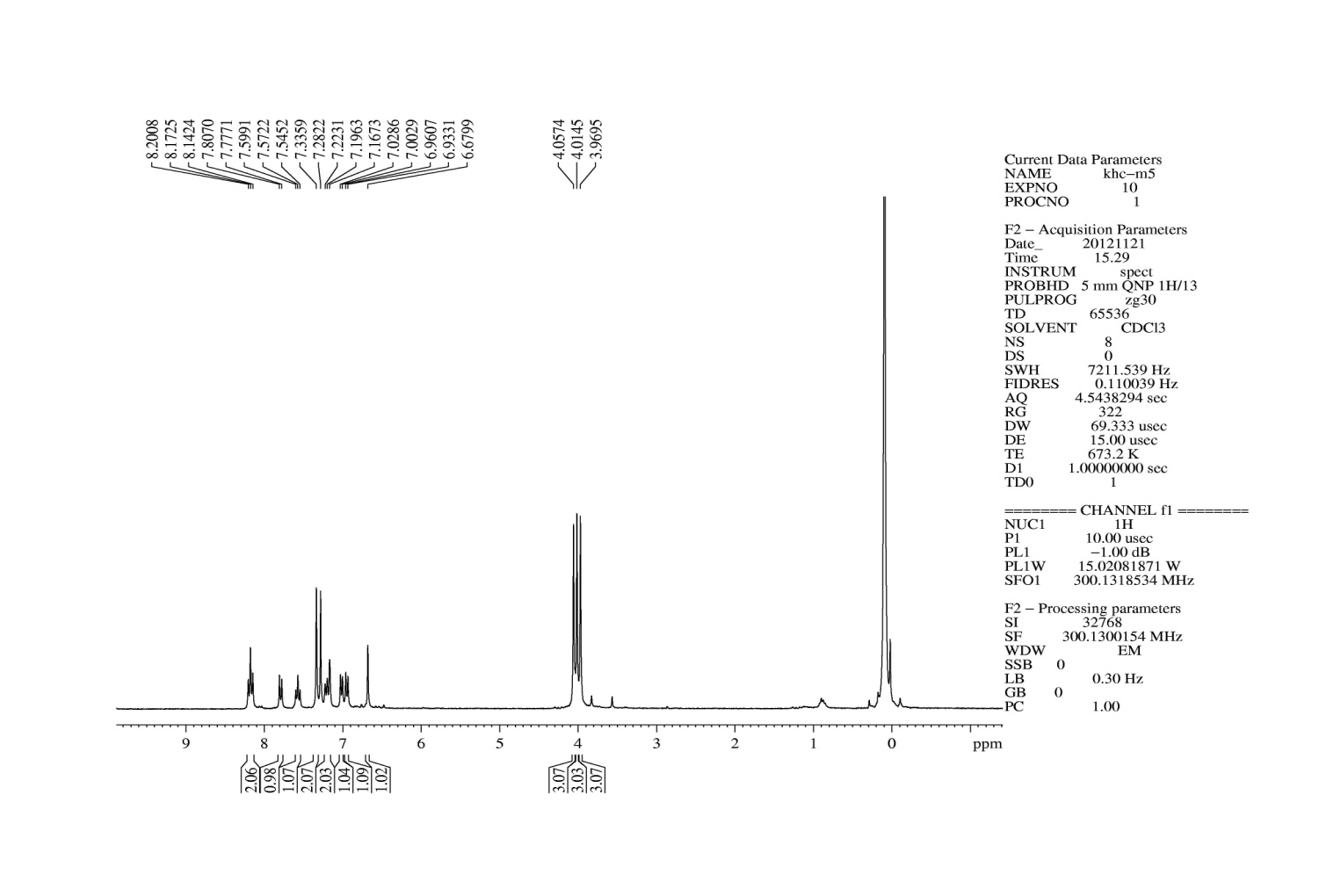


Supplementary figure 45 ^1^H NMR of compound 27


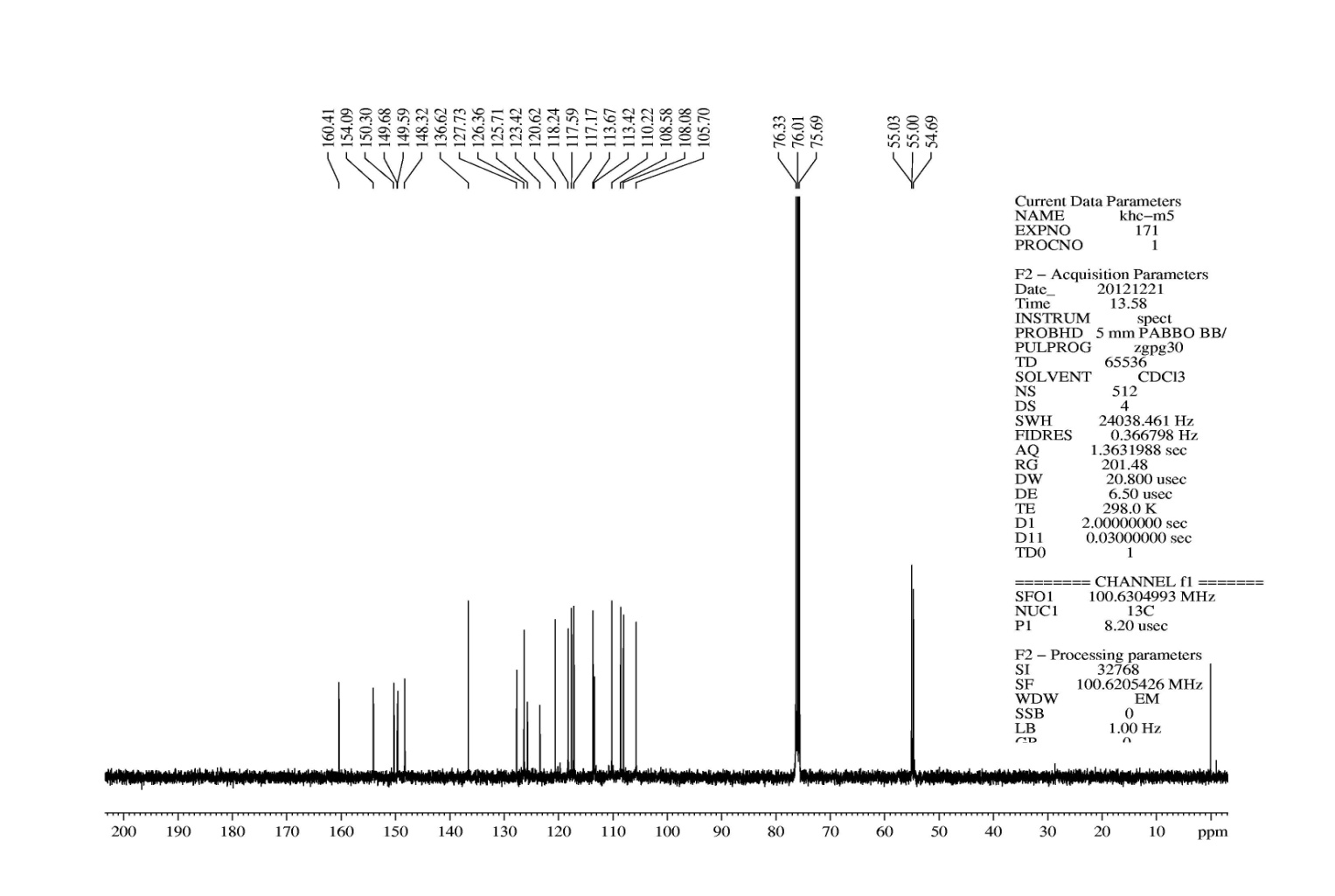


Supplementary figure 46 ^13^C NMR of compound 27


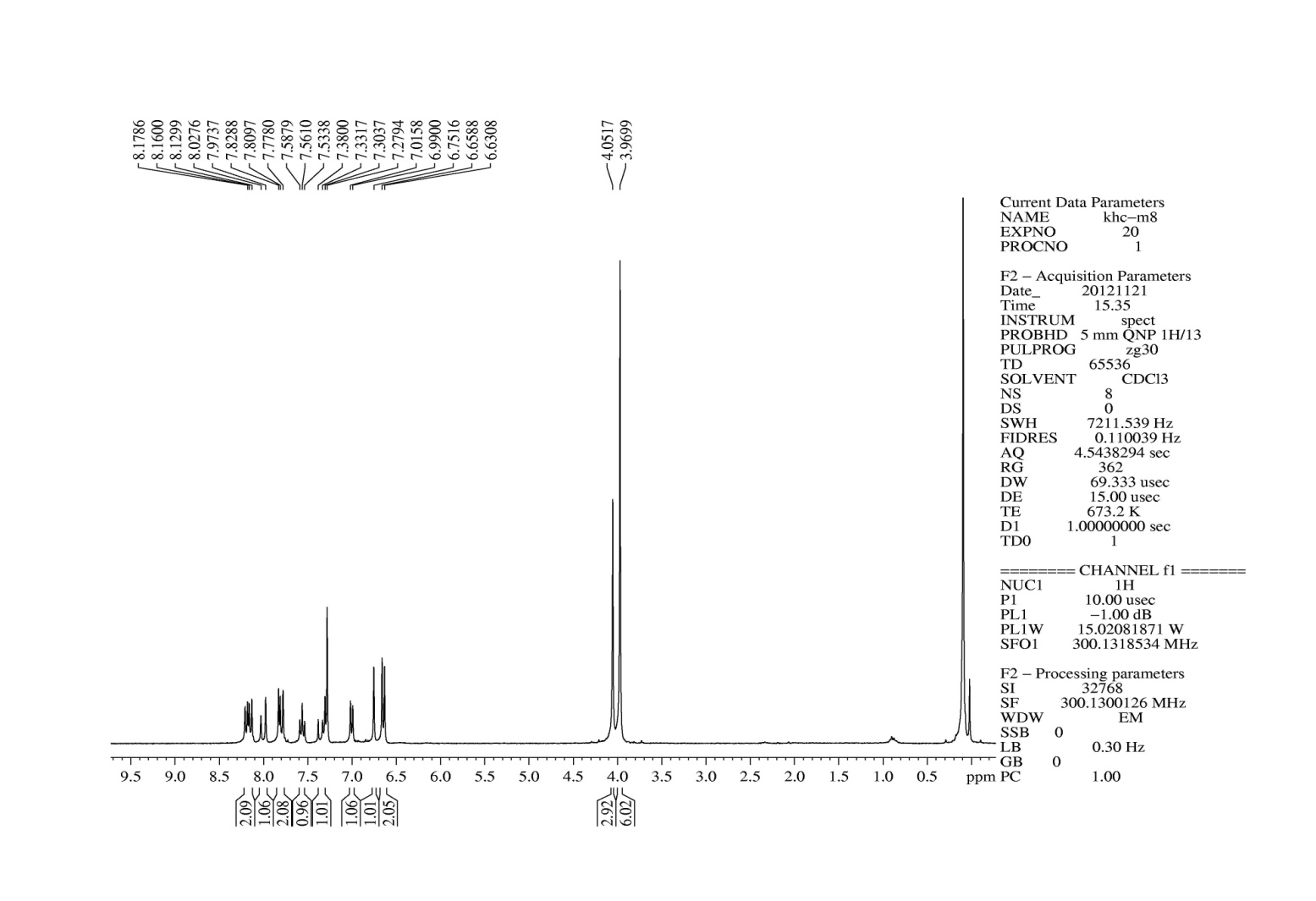


Supplementary figure 47 ^1^H NMR of compound 28


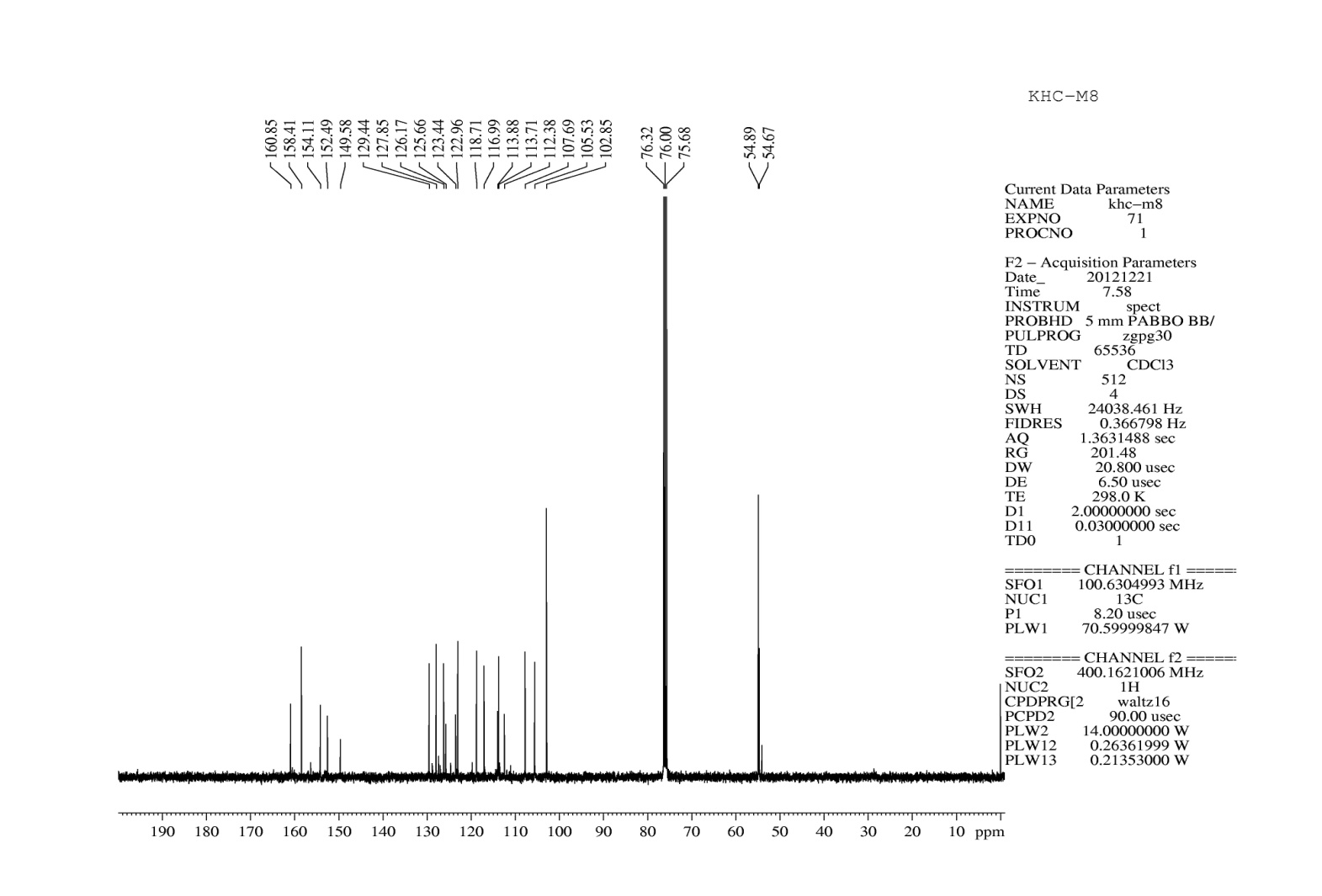


Supplementary figure 48 ^13^C NMR of compound 28


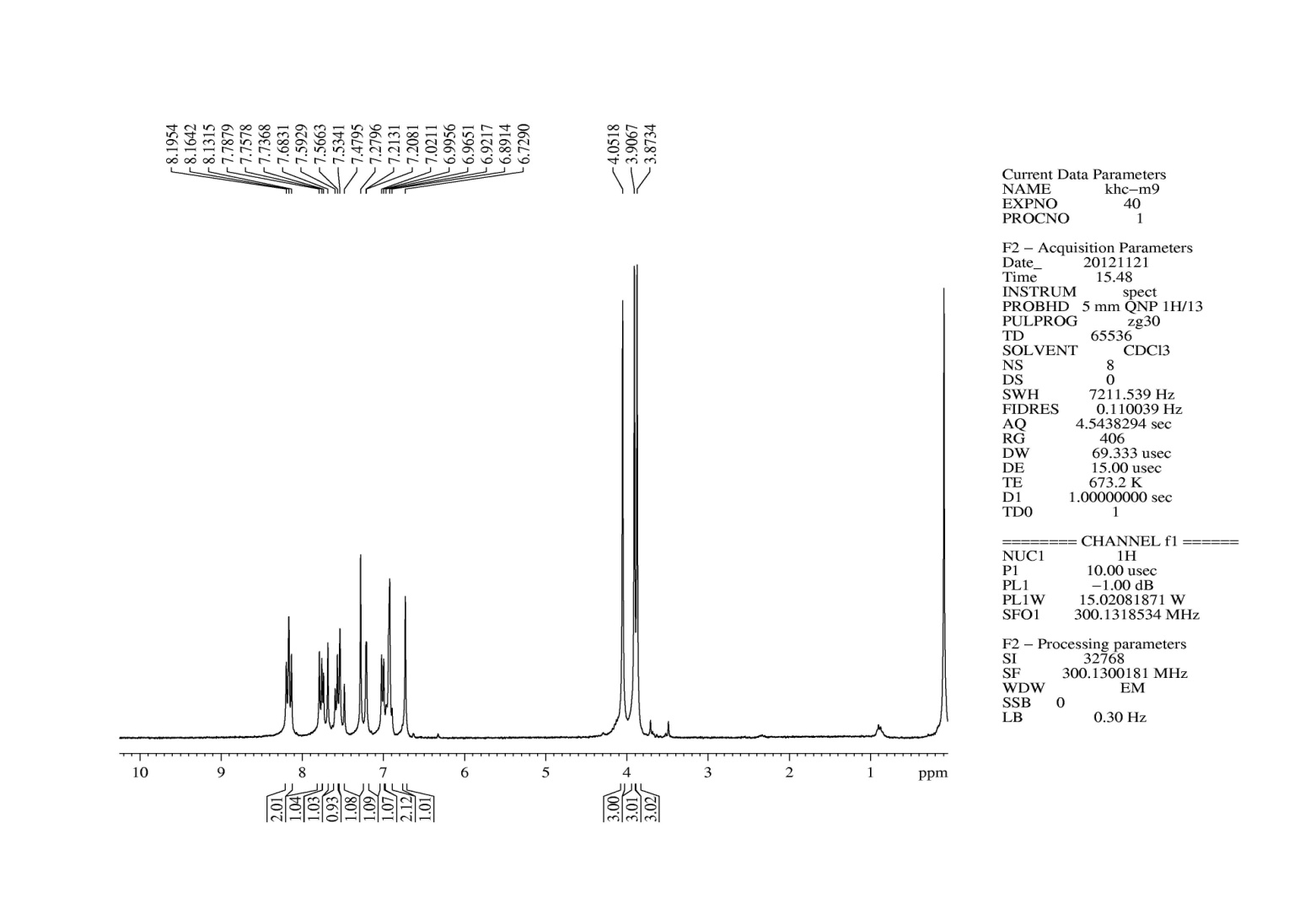


Supplementary figure 49 1H NMR of compound 29


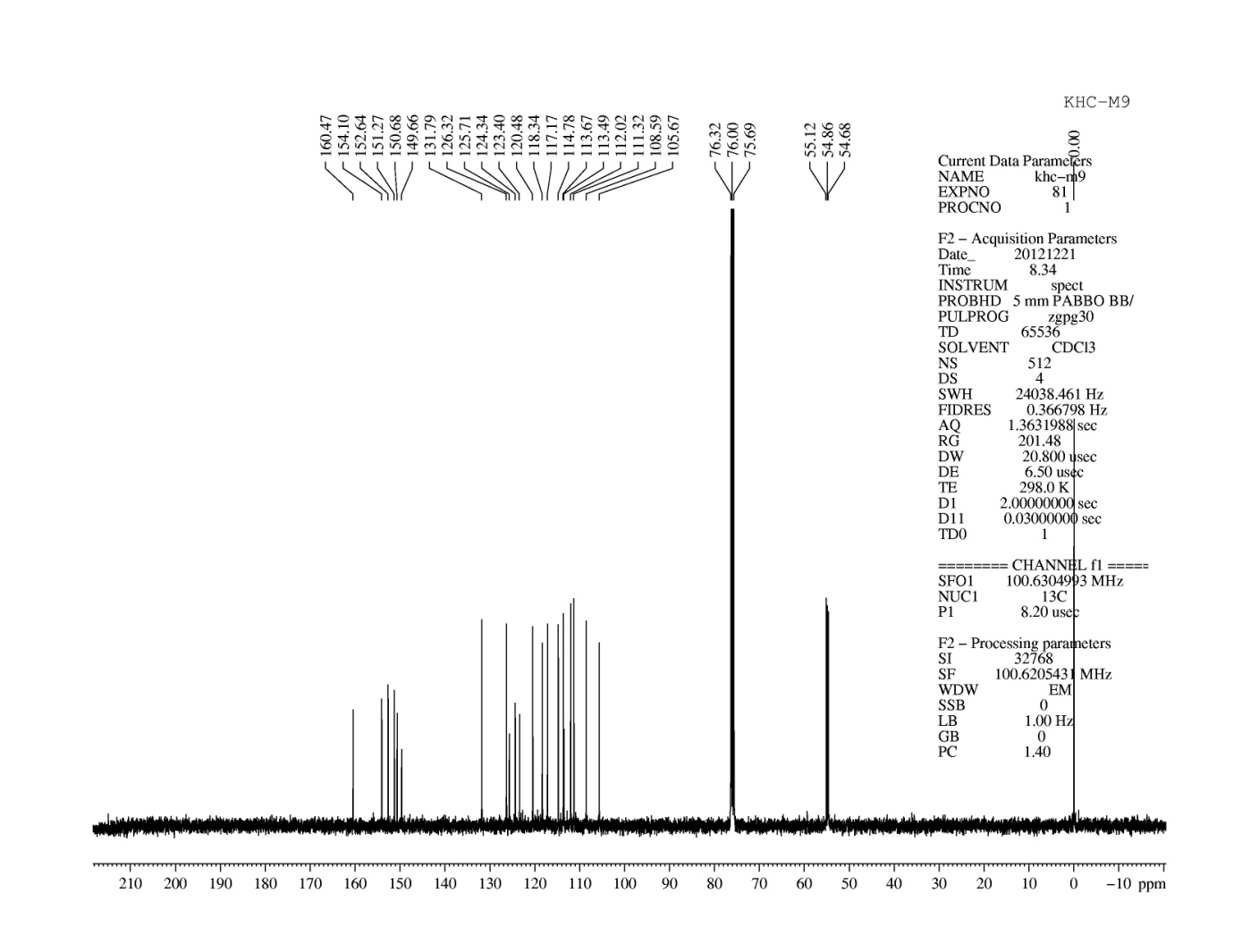


Supplementary figure 50 ^13^C NMR of compound 29


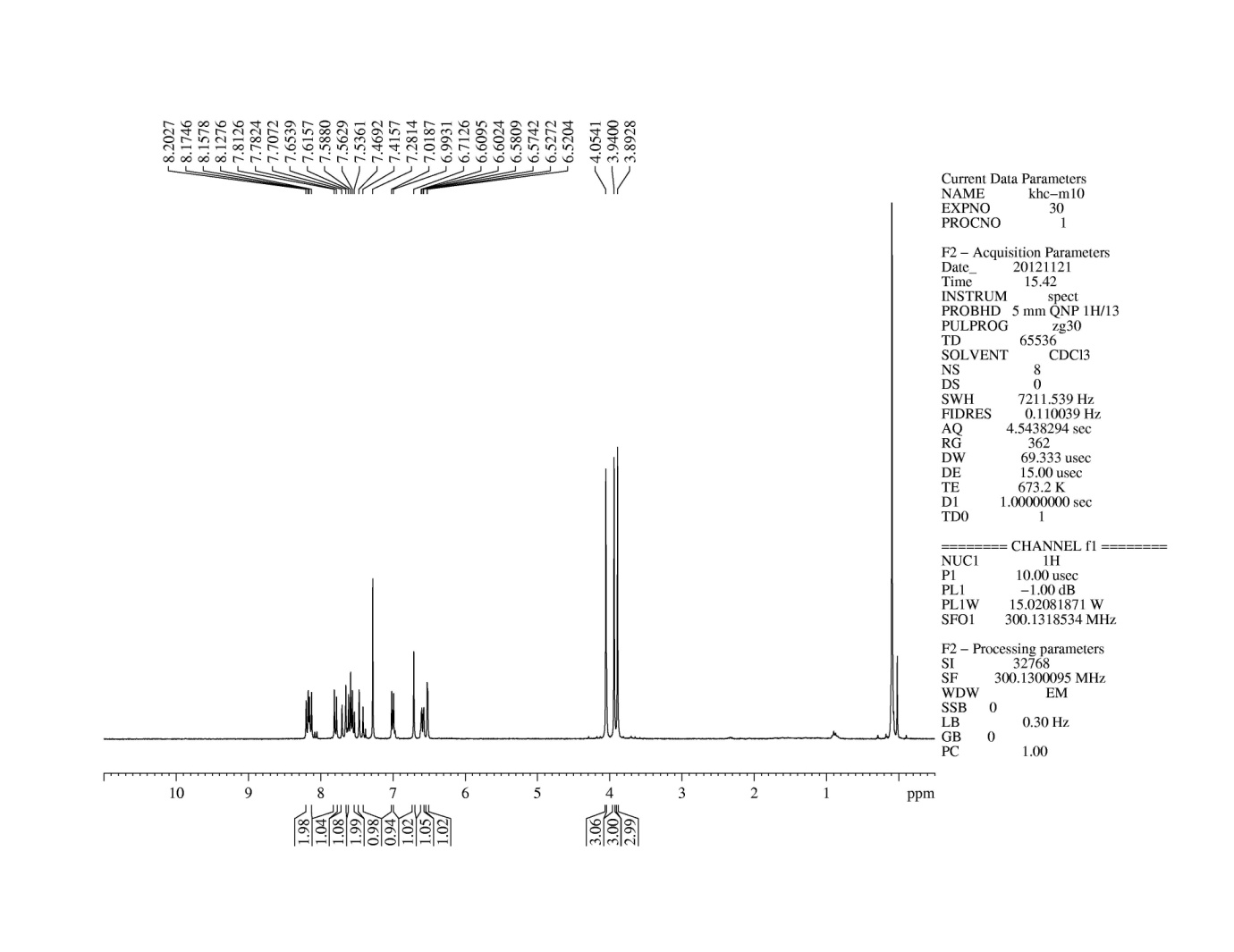


Supplementary figure 51 ^1^H NMR of compound 30


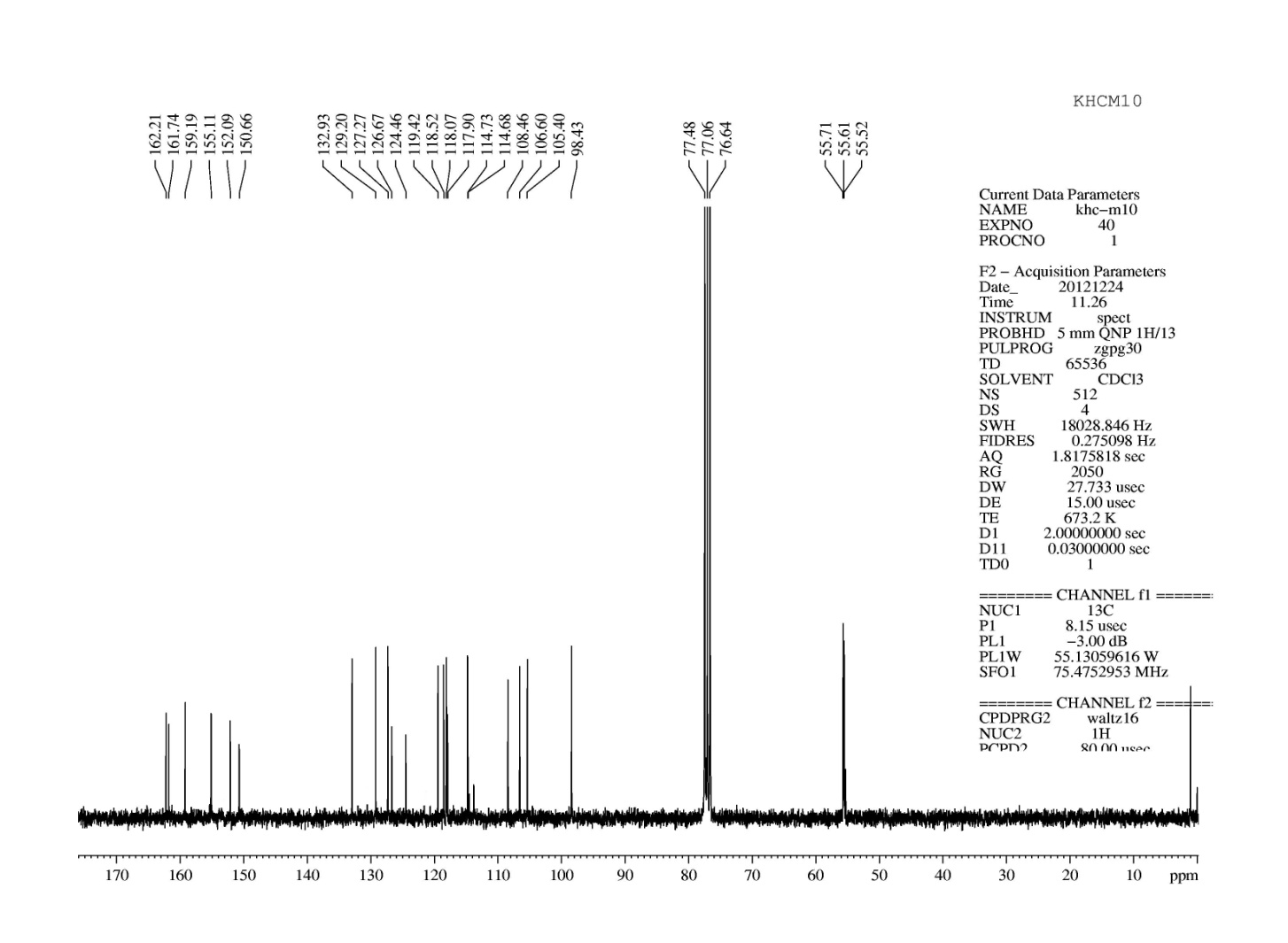


Supplementary figure 52 ^13^C NMR of compound 30


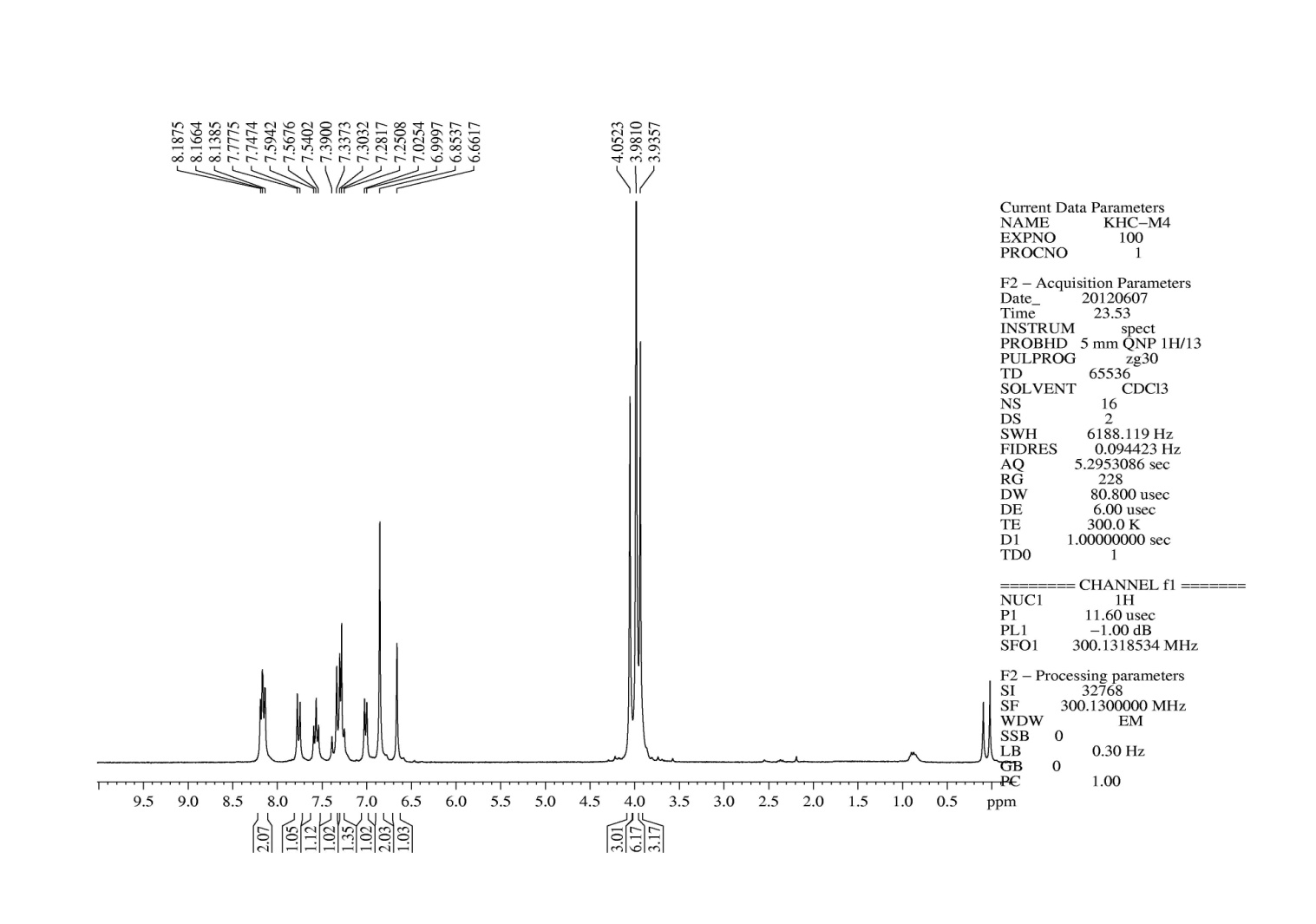


Supplementary figure 53 ^1^H NMR of compound 31


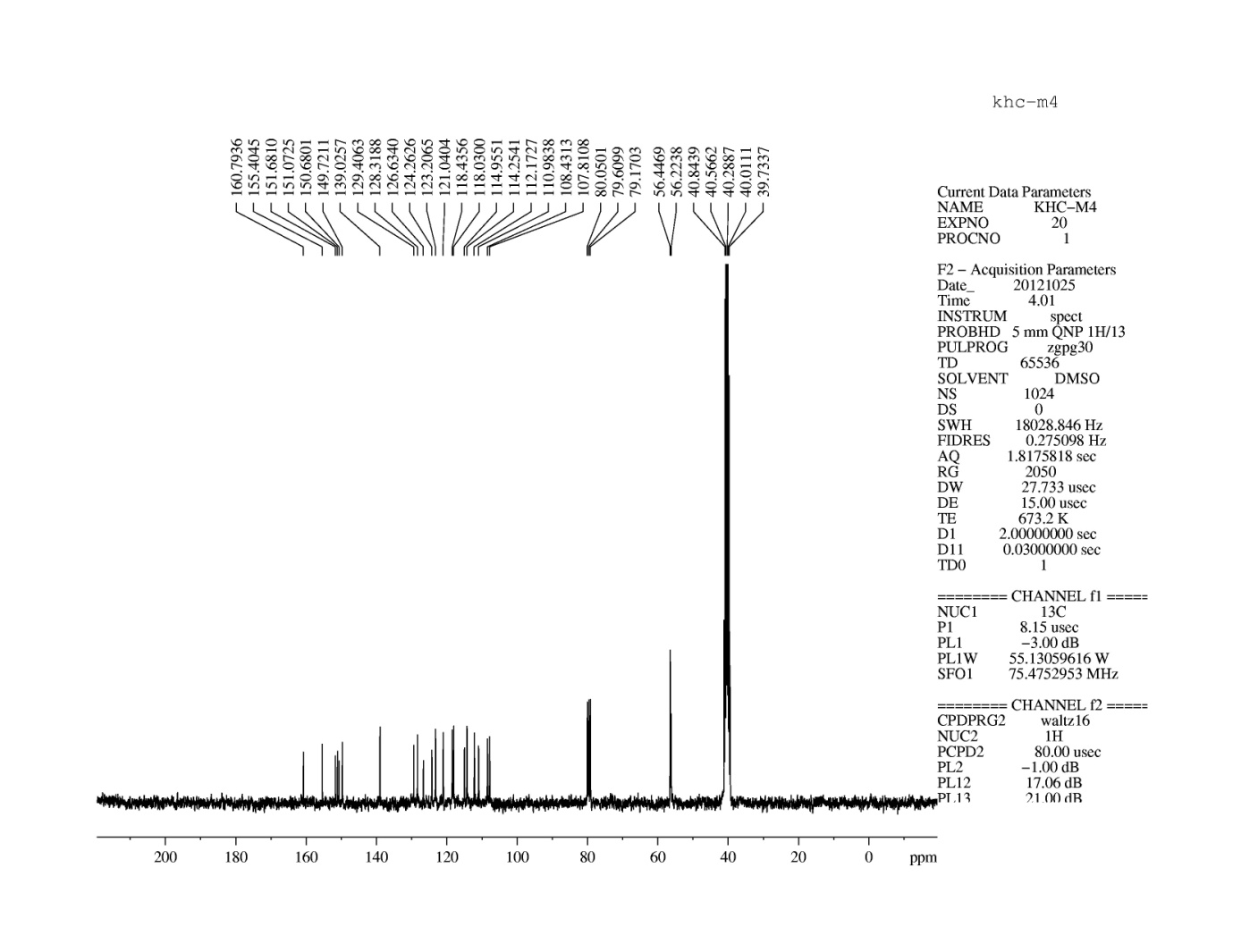


Supplementary figure 54 ^13^C NMR (CDCl_3 +_DMSO-d_6_) of compound 31


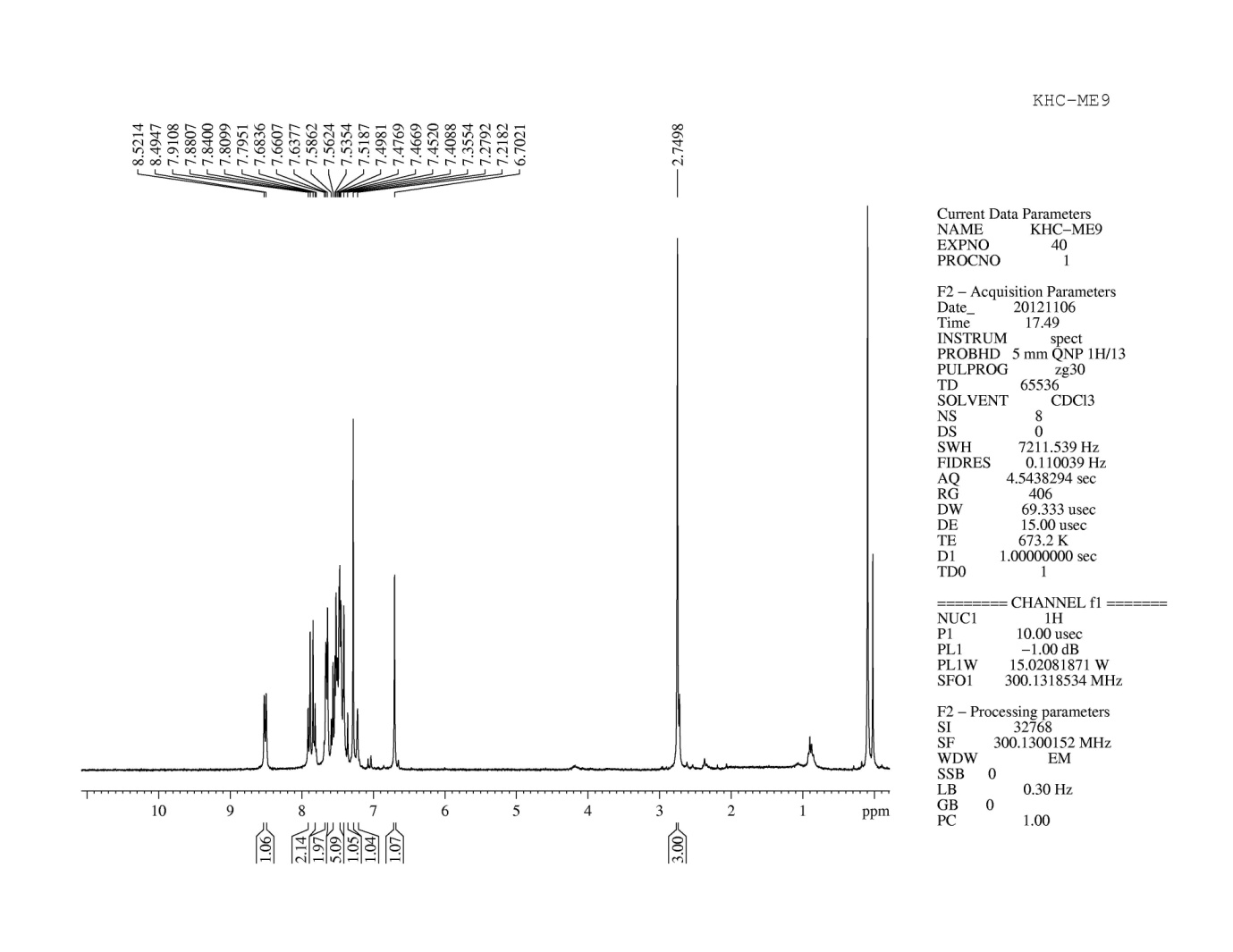


Supplementary figure 55 ^1^H NMR of compound 32


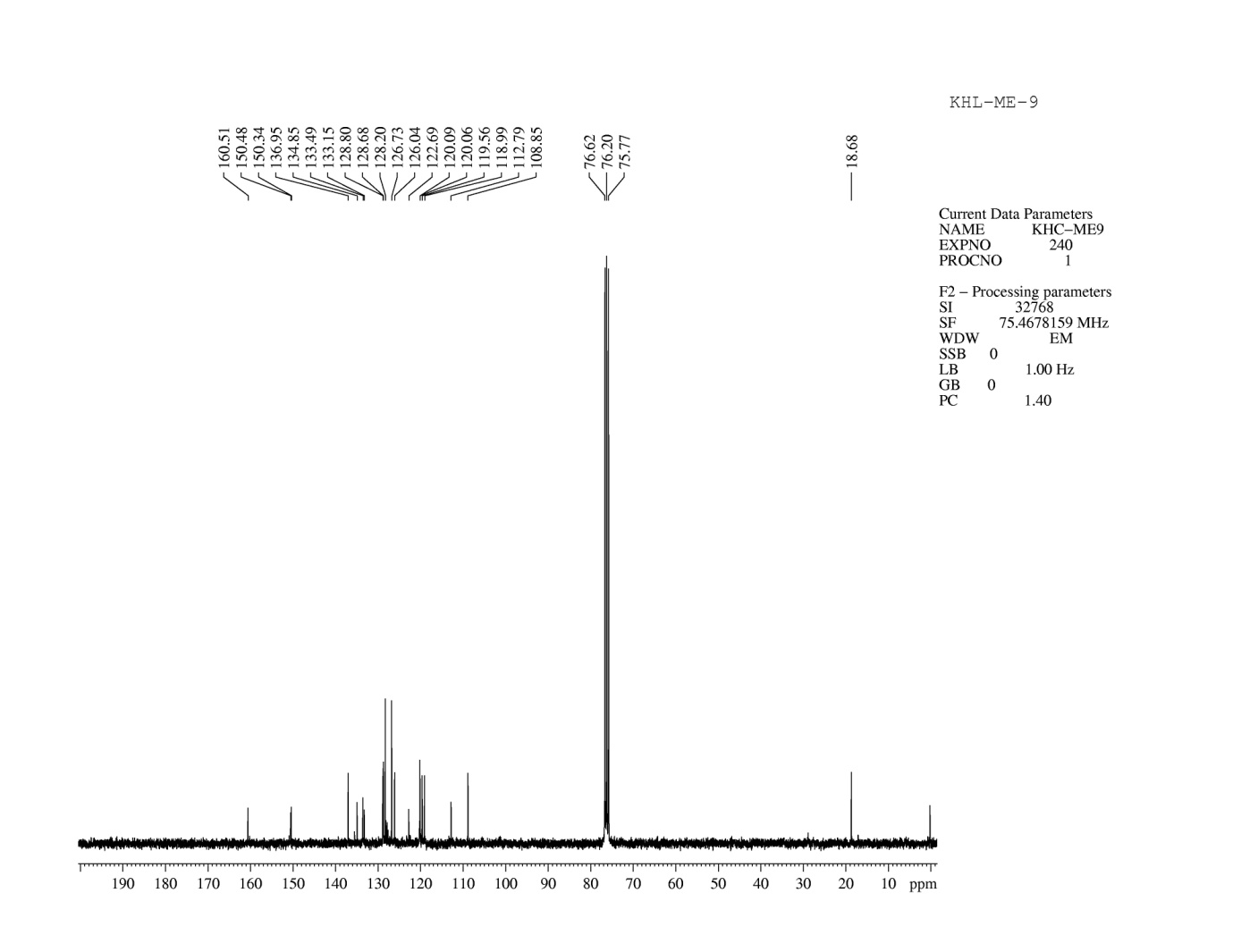


Supplementary figure 56 ^13^C NMR of compound 32


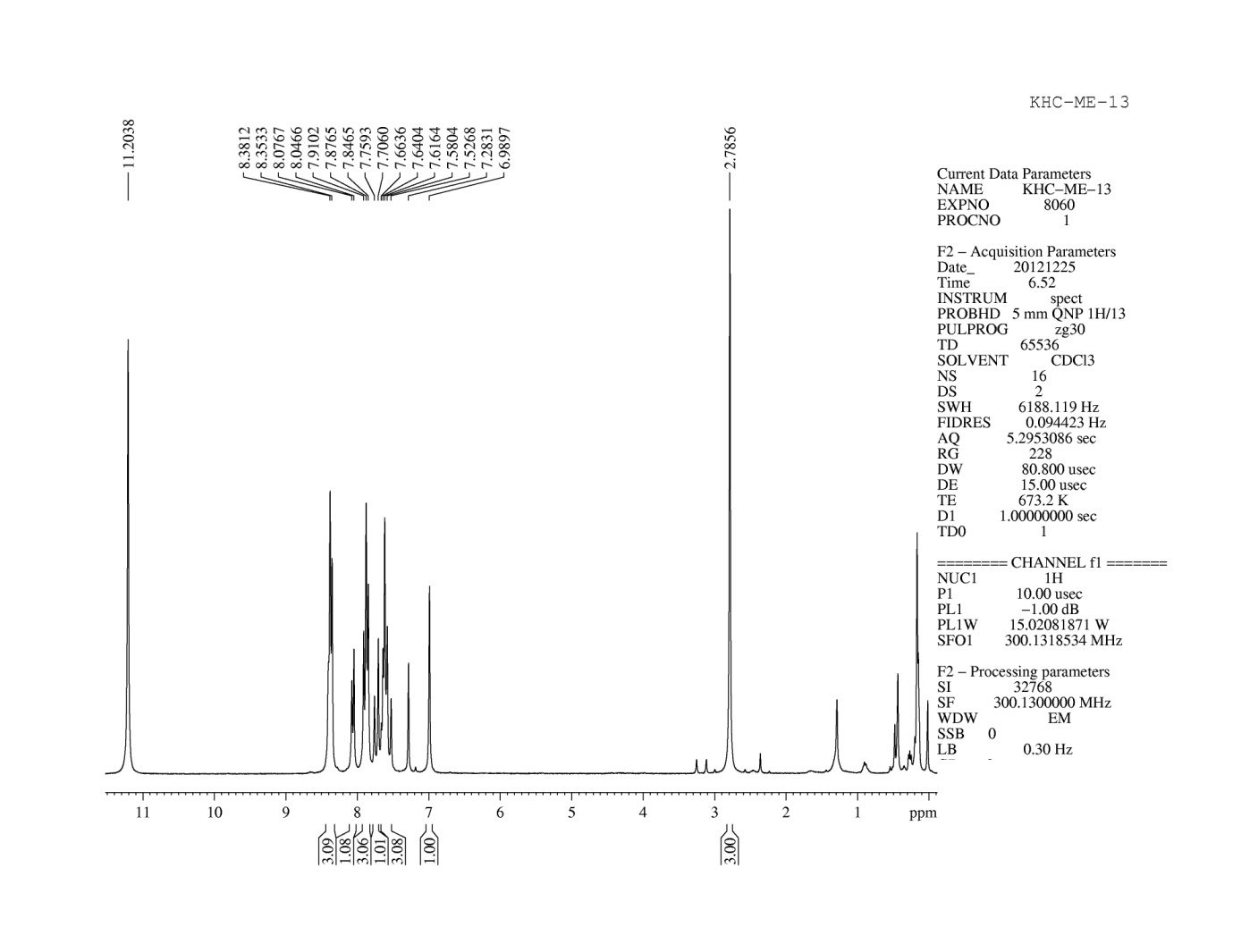


Supplementary figure 57 ^1^H NMR (CDCl_3_ + TFA-*d*_1_)of compound 33


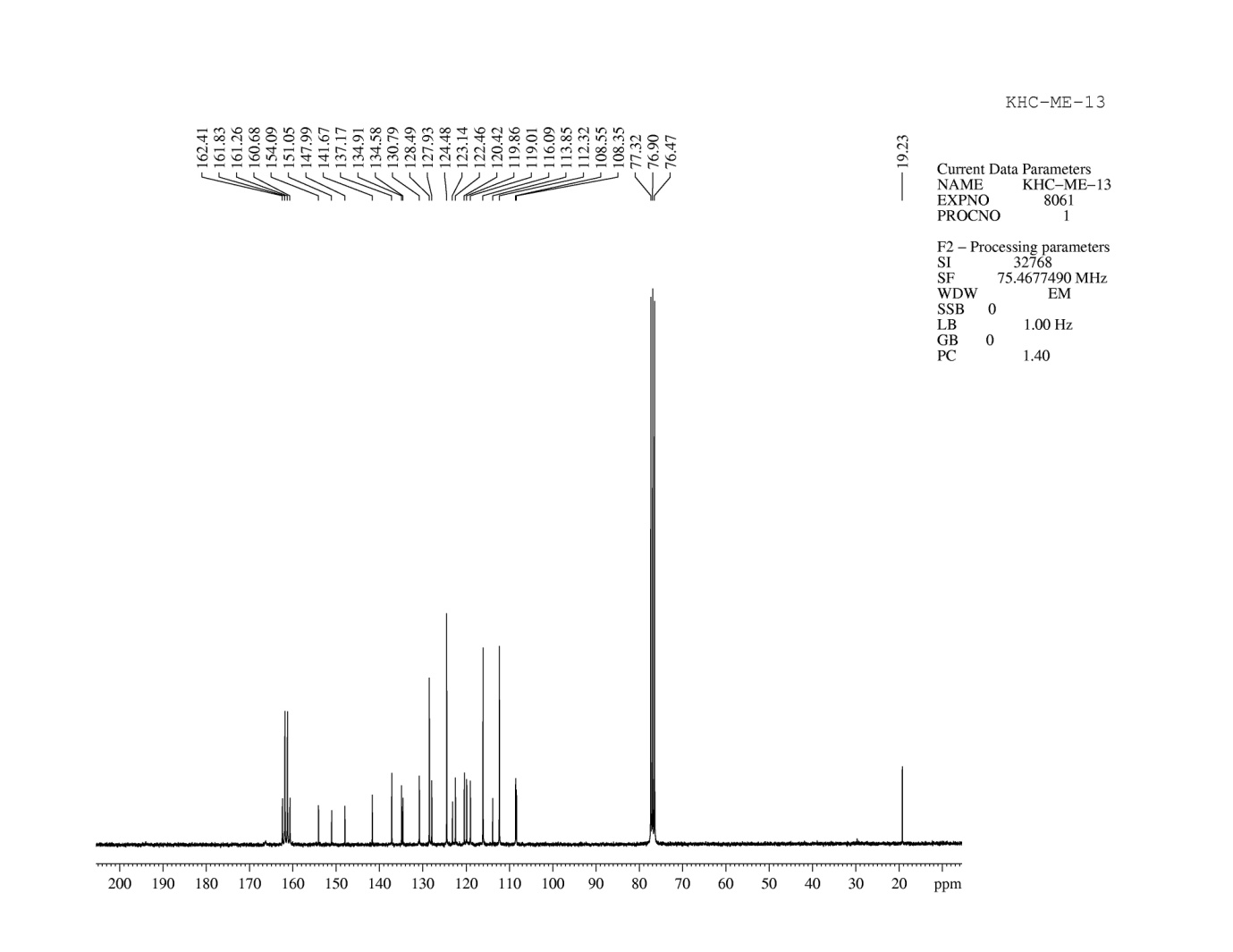


Supplementary figure 58 ^13^C NMR (CDCl_3_) of compound 33


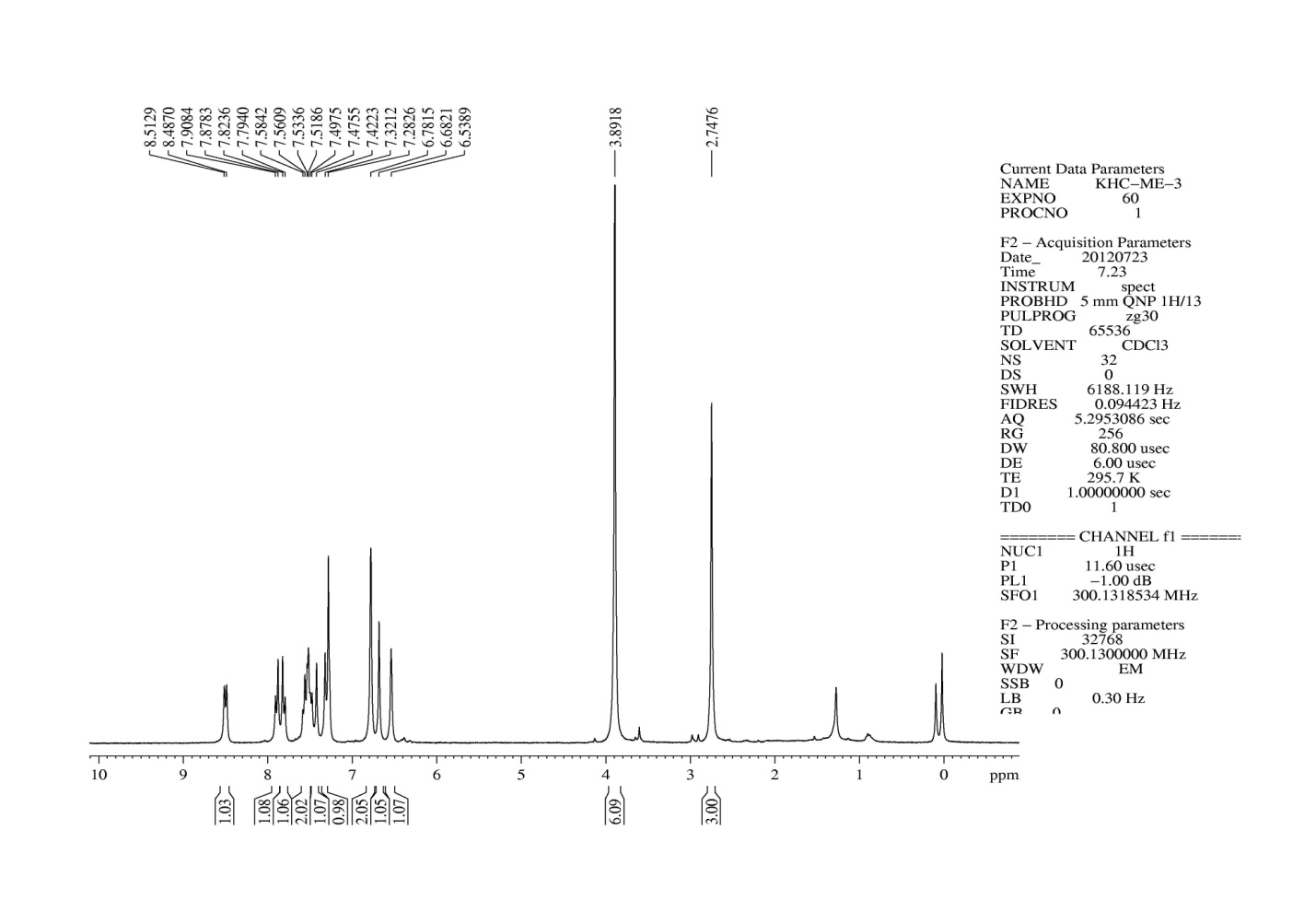


Supplementary figure 59 ^1^H NMR of compound 34


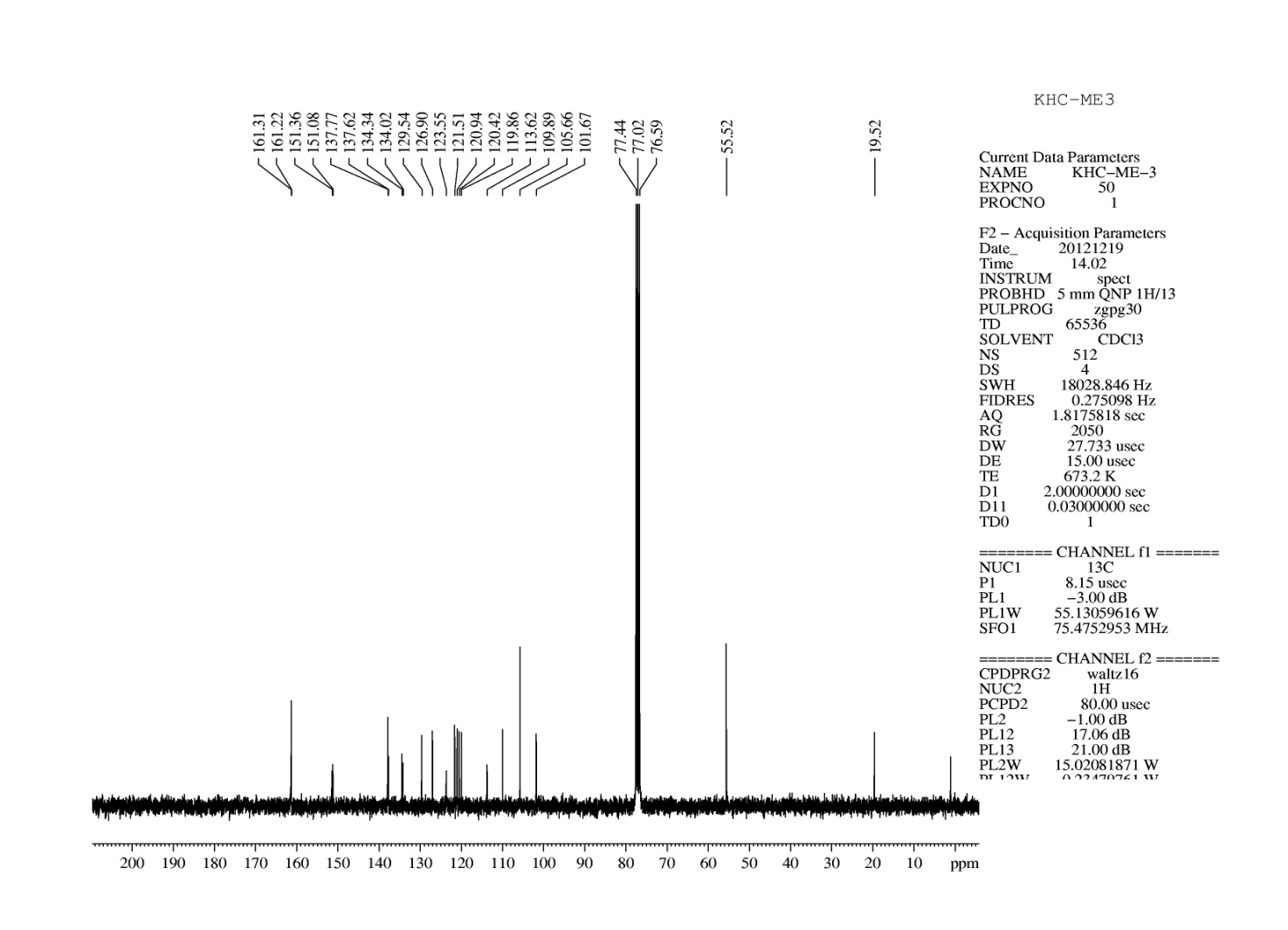


Supplementary figure 60 ^13^C NMR of compound 34


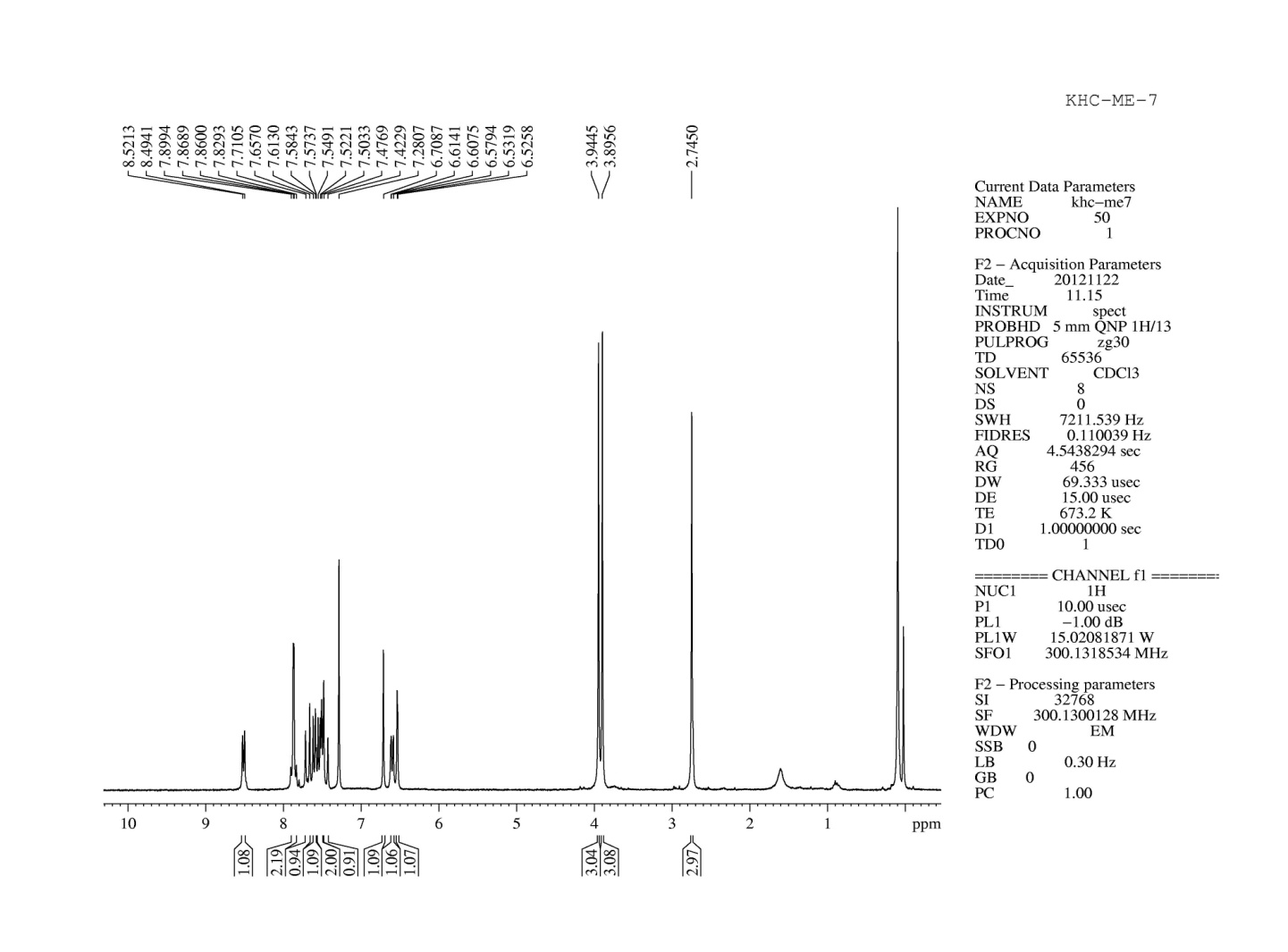


Supplementary figure 61 ^1^H NMR of compound 35


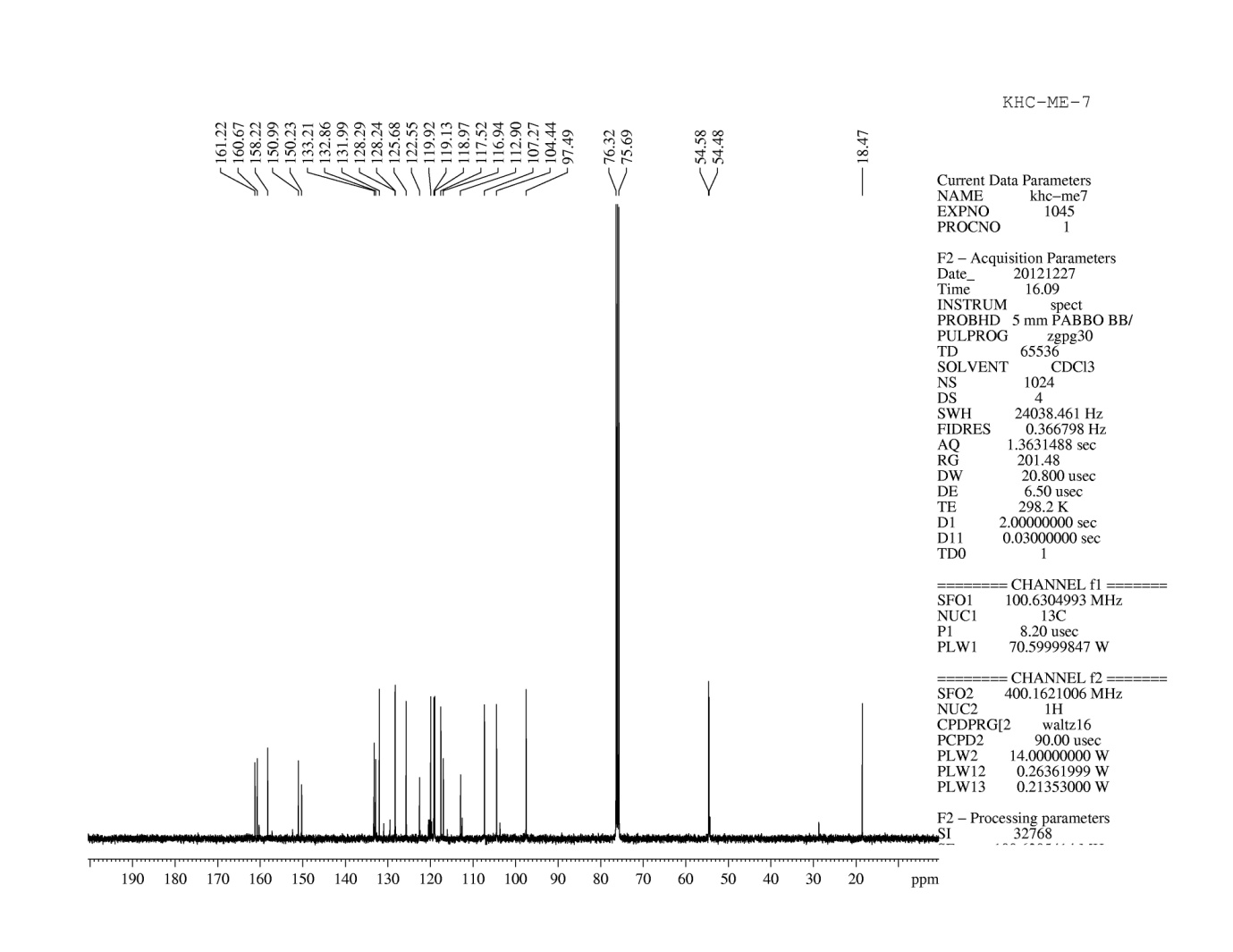


Supplementary figure 62 ^13^C NMR of compound 35


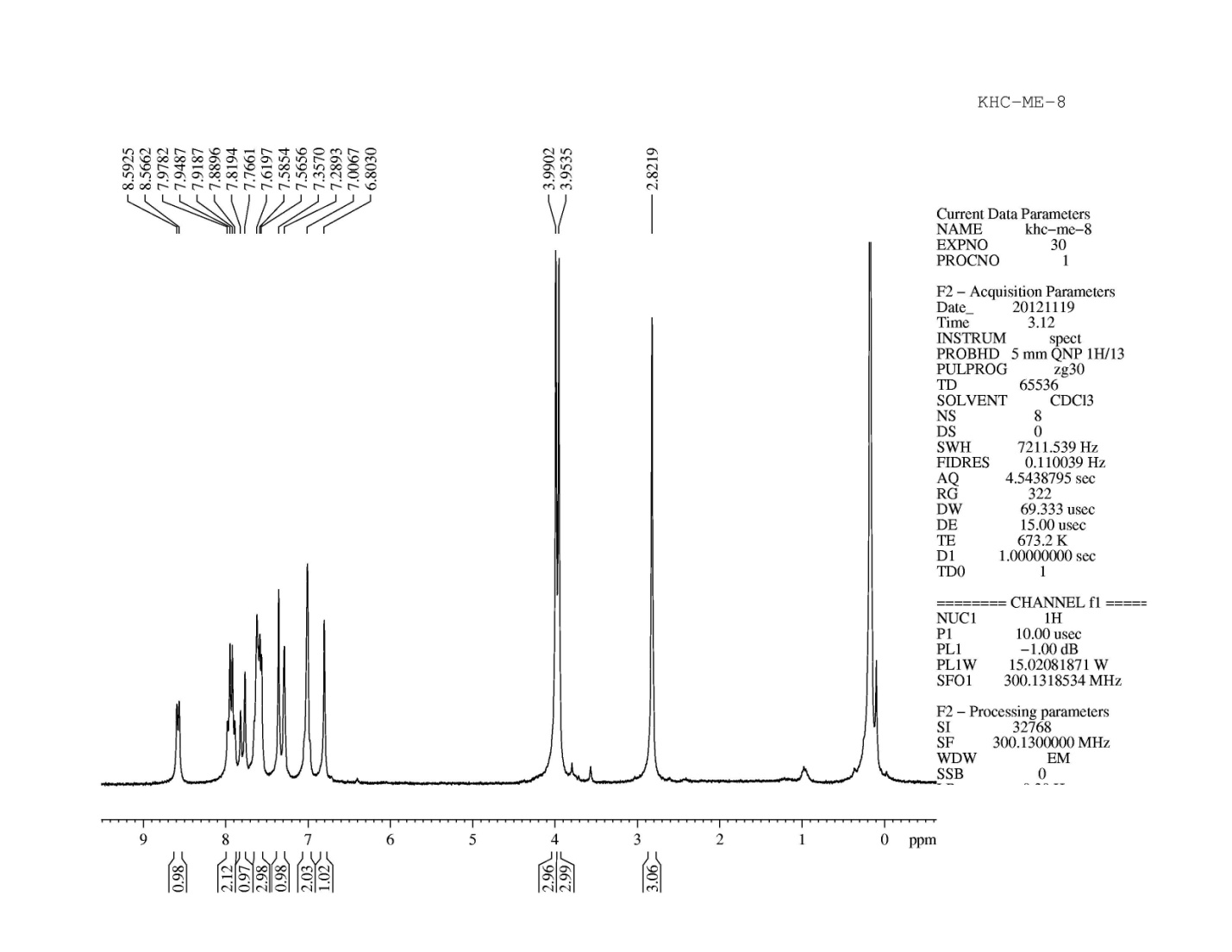


Supplementary figure 63 ^1^H NMR of compound 36


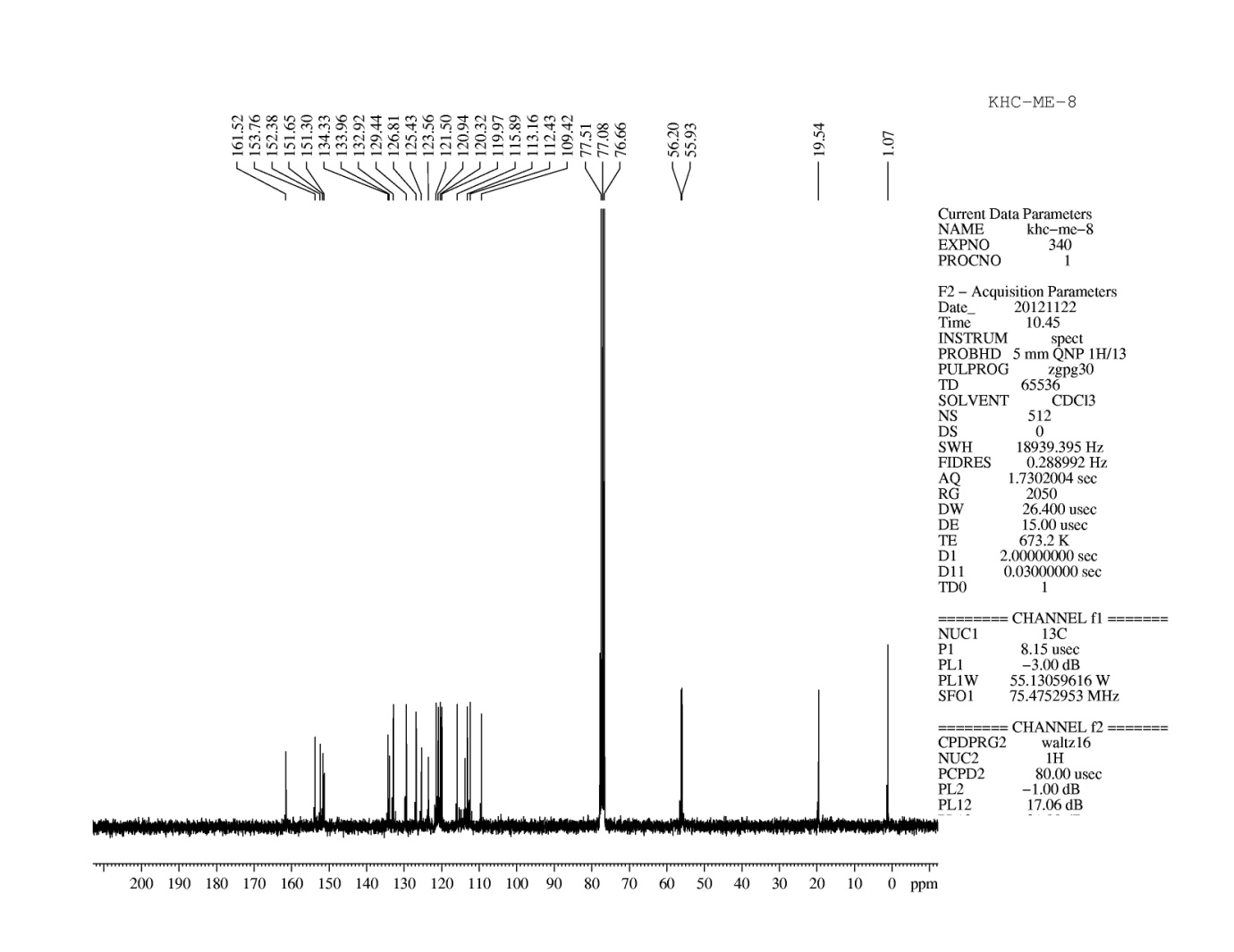


Supplementary figure 64 ^13^C NMR of compound 36


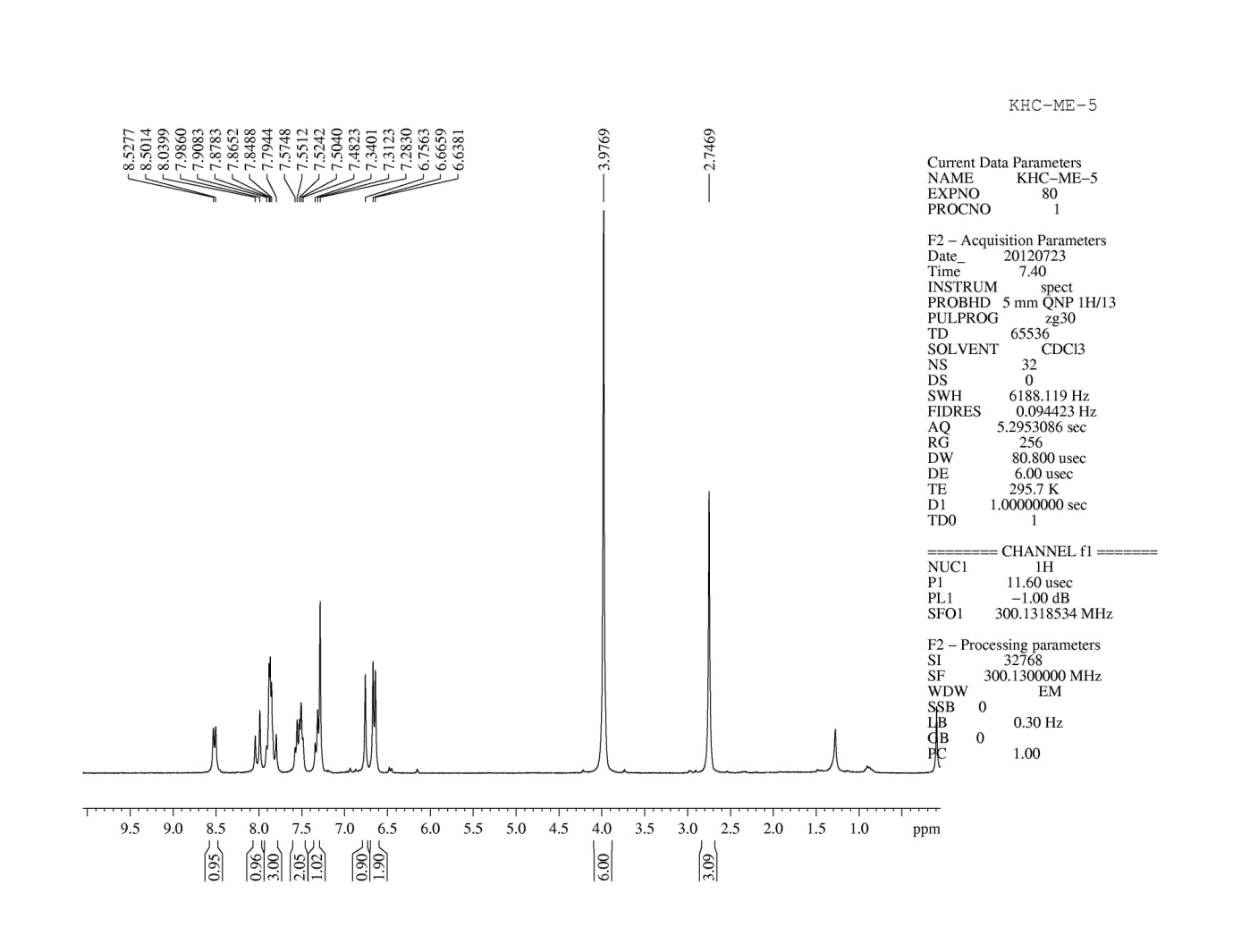


Supplementary figure 65 ^1^H NMR of compound 37


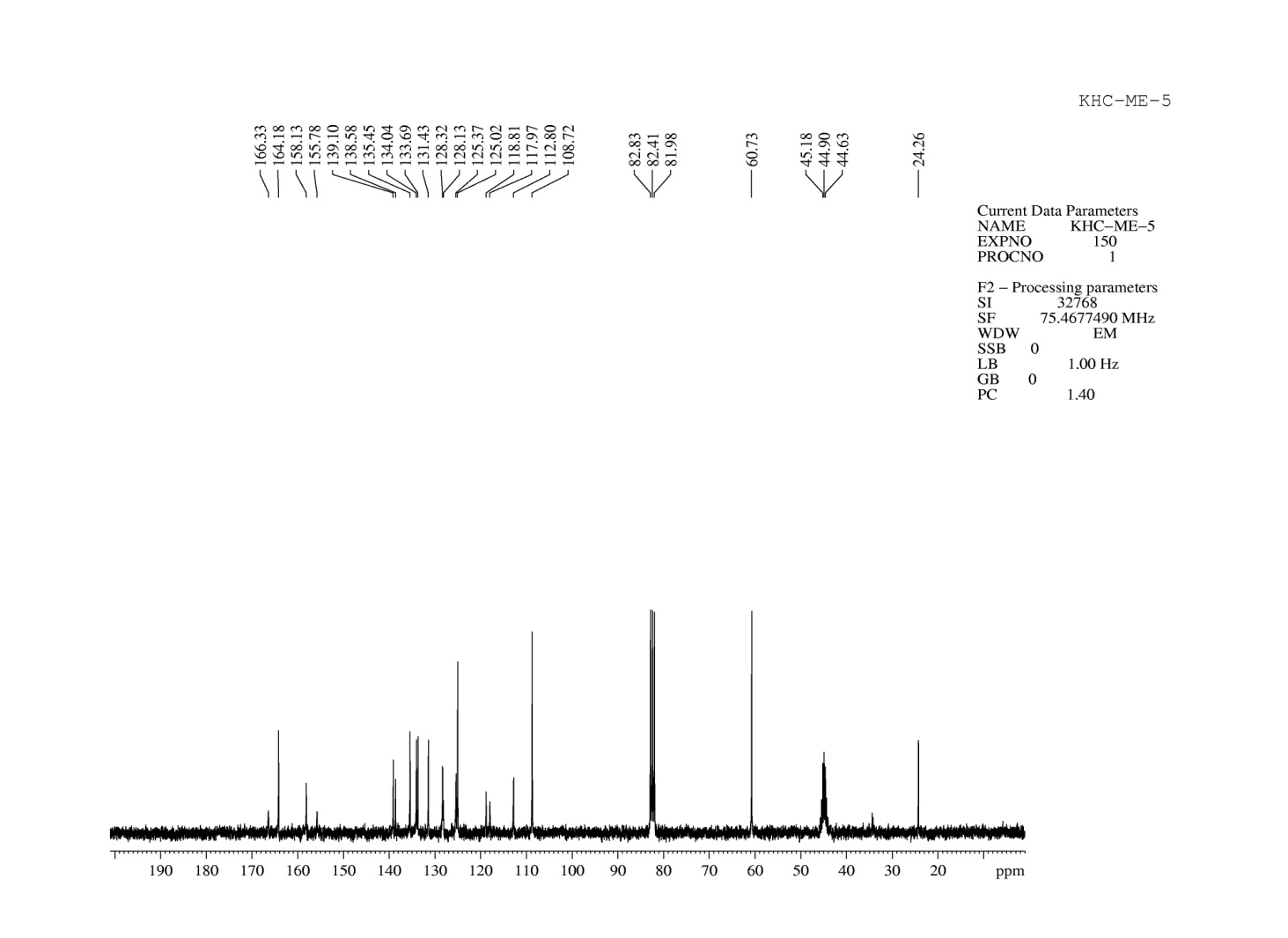


Supplementary figure 66 ^13^C NMR (CDCl_3_, +DMSO-*d*_6_ )of compound 37


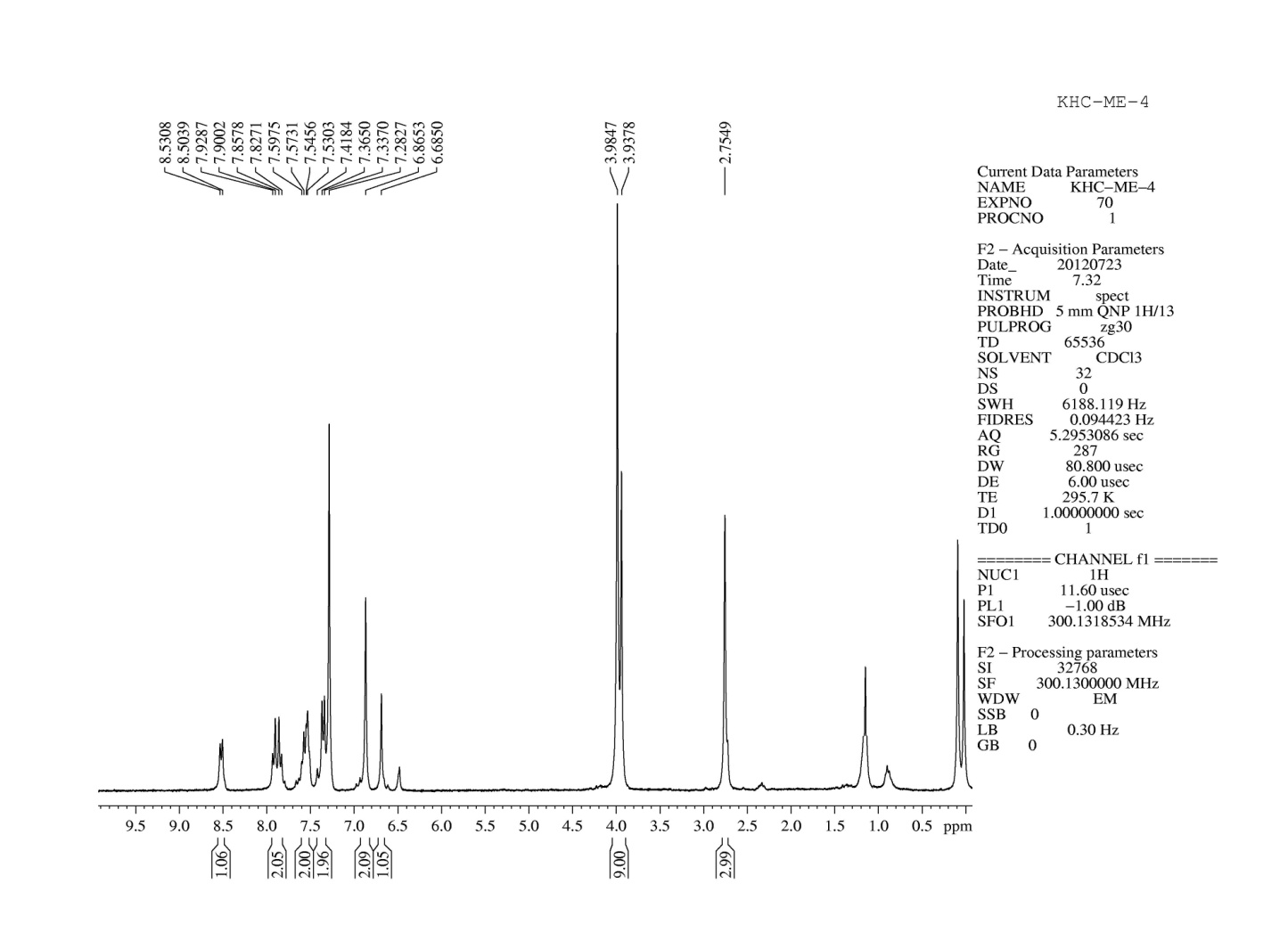


Supplementary figure 67 ^1^H NMR of compound 38


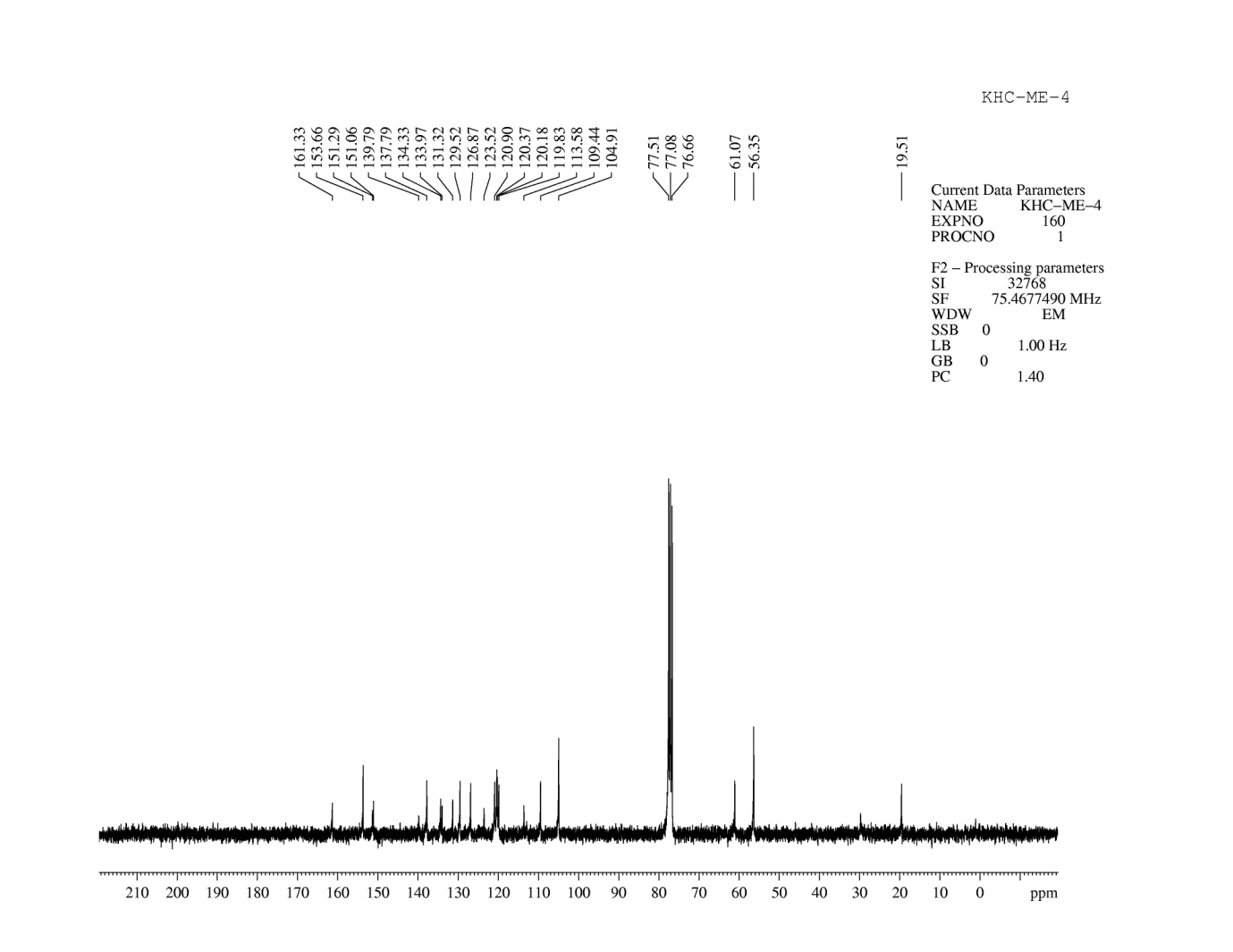


Supplementary figure 68 ^13^C NMR of compound 38


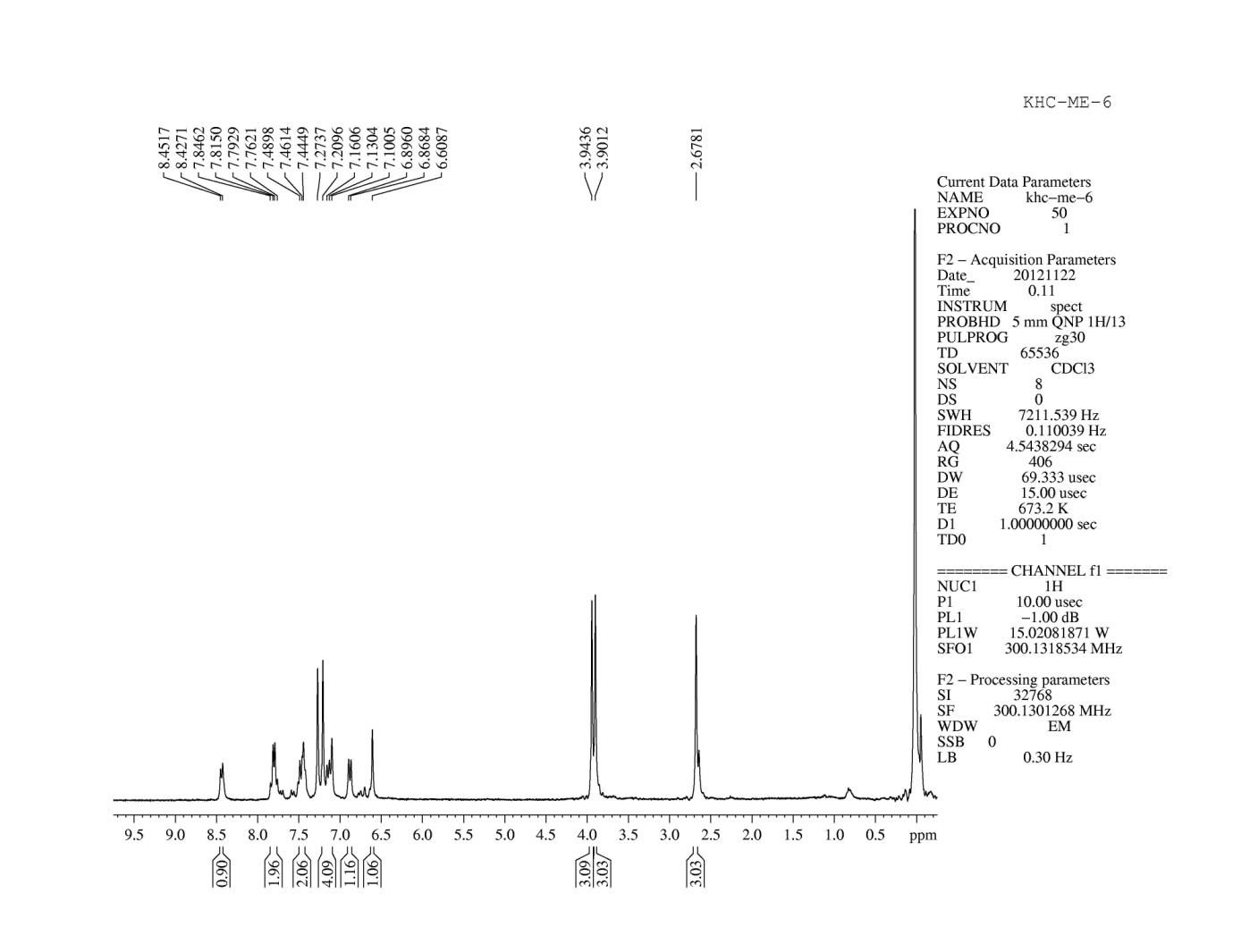


Supplementary figure 69 ^1^H NMR of compound 39


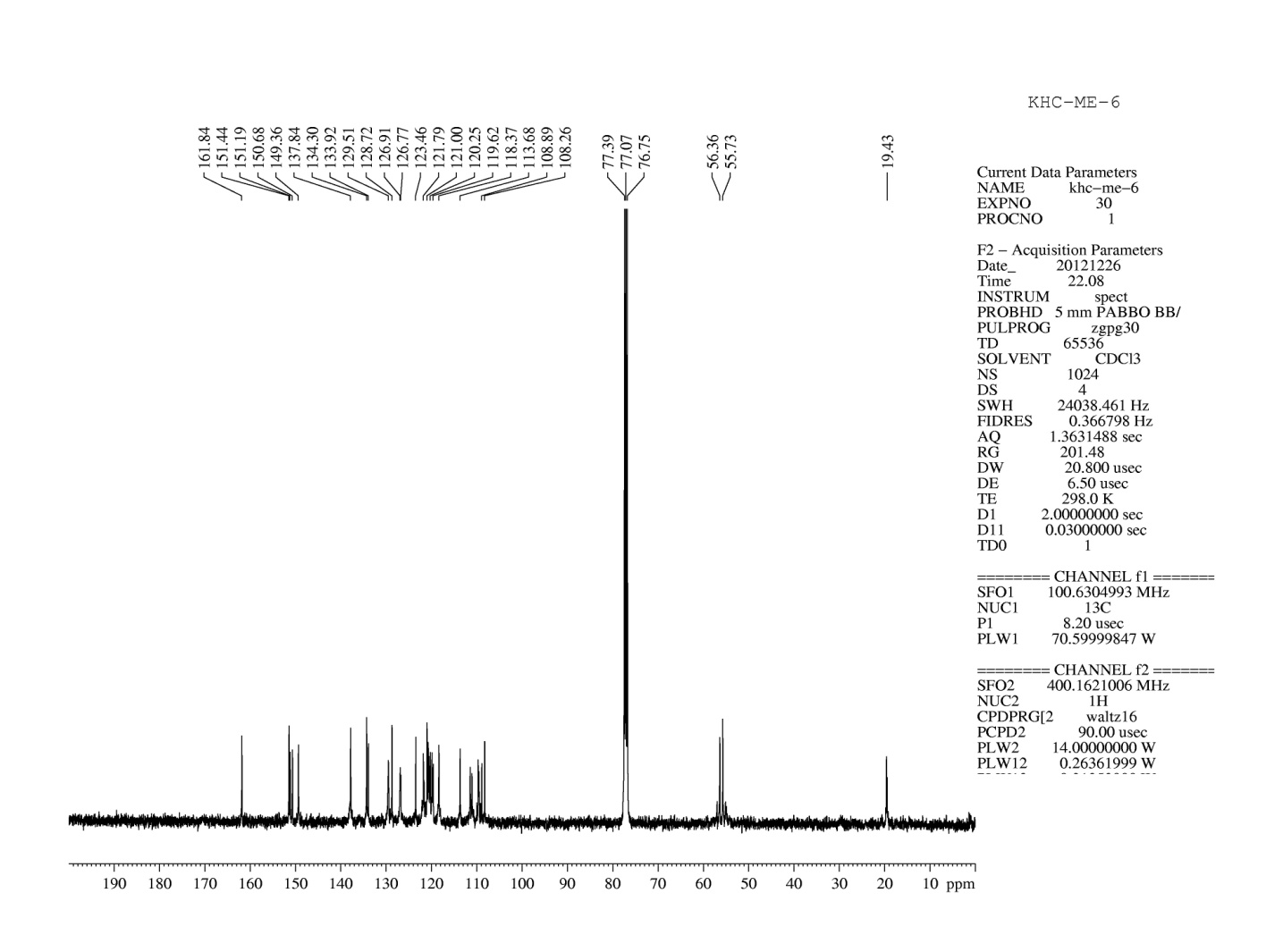


Supplementary figure 70 ^13^C NMR of compound 39

**Biological assays**

**Comet Assay**

Comet assay was performed as described by Olive et al ^3^ with slight modifications. MDA-MB-231 cells treated with compound **19** at 12µM for 48h. The harvested cells were mixed with low melting point agarose and pipet the 200μl of cell suspension onto agarose coated surface of frosted slides. The samples were lysed for overnight at 4^°C^ after the lysis, electrophoresis performed at 40mA for 30mins. The slides neutralized by water, stained with propidium iodide and images were captured by fluorescence microscope. As shown in Supplementary Fig. 71 the treated samples (B) have increased length of DNA tail as compared to control sample (A). This increase in the tail length of DNA occurs due to DNA damage which is induced by compound **19** in MDA-MB-231 cells.


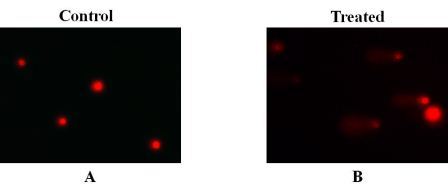


**Supplementary Fig.** **71** Fluorescent images of comet assay showed an increase in DNA damage (image B versus A) when cells were treated with compound **19**.

**Compound 19 does not inhibit the activity of FEN1 protein**

**Purification of FEN 1:** Full length FEN1 DNA template was taken from pShuttle-FEN1hWT, a gift from Sheila Stewart (Addgene plasmid#35027) is subcloned into a pET41a expression vector containing C-terminal His-tag. pET41FEN1His freshly transformed into expression host BL21DE3 *E. coli* cells (Novagene), single colonies picked and inoculated in 5ml of LB media containing 50μg/ml of Kanamycin, grown for 3 to 4 hrs and further subcultured in 100 ml of LB medium containing 50μg/ml of Kanamycin and grown at 37°C till the OD reached to 0.6-0.7, induced at 0.5 mM IPTG at 16°C for 18hrs. Pellets were collected and lysed in 20 mM tris 150 mM NaCl (Lysis buffer) containing 1mM PMSF and 1mM benzamidine hydrochloride and purified by Ni-NTA affinity chromatography.

**Flap cleavage assay:** All oligos were purchased from Integrated DNA Technologies and dissolved in nuclase free water at concentration of 100μM. FAM-labeled 25+6 Flap-mer (5’-GCCCTGCTGATCTACCAATCGATCGACGTAC-3’*6FAM)) was annealed with 52- mer (5’-GTACGTCGATCGATTGGTAGATCAGTGTCTATGTATGTCAGTGAGATAGTAC-3’) and 27-mer (5’-GTACTATCTCACTGACATACATAGACA-3’) to form a complementary double strand flap cleavage substrate. The reaction mixture (20μl) were prepared by 1 picomole annealed DNA substrate, 0.01 picomole purified FEN1 protein and containing freshly prepared assay buffer of 20 mM Tris pH 7.5, 10 mM MgCl2, 10 mM KCl, and 1 mM dithiothreitol. Reactions were performed at 37°C for 20 min in the presence (5, 10, 20 µM) or absence of compound **19** and stopped by adding 10 μl of stop buffer (90% formamide and 10% 50mM EDTA). The cleaved and uncleaved DNA molecules are separated on 15% Urea gel and bands were detected by Image Quant LAS 4010 (GE Healthcare). The gel picture (Supplementary Fig. 72) showed that the amount of cleaved oligo (25 Mer) remains same in compound **19** treated (Lane 4-6) and DMSO control sample (Lane 1) whereas the sample treated with positive control NSC13755 have less clevage the 25 mer oligo that indicates the inability of compound **19** for FEN1 activity inhibition.


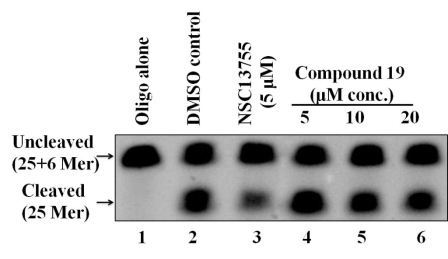


**Supplementary Fig. 72** A gel picture of flap cleavage activity performed by FEN1 protein in presence or absence of compound **19**. Unlike positive control NSC13755 compound **19** was unable to inhibit the activity of FEN1 protein significantly (lane 3 versus lanes 4-6).

**Compound 19 was not able to inhibit the activity of PARP1 protein**

**Purification of PARP1:** pET-PARP1 construct was a kind gift from Dr. John M. Pascal, (Thomas Jefferson University, Philadelphia, USA). Human PARP1 full length protein was purified as described previously ^4,5^.

**PARP1 Activity assay:** A total 25 µl reaction mixture contains 1.25 μM NAD, 2.5 μg/mL PARP, 15μg/mL nicked DNA substrate (The sequence of nicked DNA substrate is same as used in ligation assay but they are without fluorescent labelled) and PARP assay buffer (50mM Tris, 2mM MgCl2 at pH 8.0) incubated at room temperature for 20 minutes. After that 10μl of 2 M KOH and 10μl of 20% acetophenone added and incubated at 4˚C for 10 min then 45 μl of 88% formic acid added and heated at 110˚C for 5 min. Samples were cooled and fluorescence measurement carried out at excitation 360nm and emission range 445nm. As shown in Supplementary Fig. 73, the sample having only NAD have maximum fluorescent intensity whereas after the addition of PARP in reaction mixture, it utilizes the free NAD for DNA repair process that results the unavailability of free NAD and less fluorescent intensity. Unlike the Olaparib (a known PARP1 inhibitor) the addition of different concentration (0, 5, 10 and 20 µM) of compound **19** not able to increase the fluorescent intensity of NAD which indicates that the inability of compound **19** to inhibit the PARP1 that results the less fluorescent intensity of free NAD in control and treated samples.


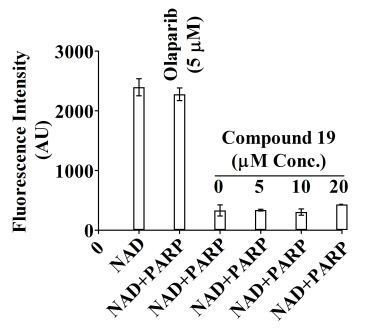


**Supplementary Fig. 73** Graph showing the effect of compound **19** on PARP1 activity. The fluorescence intensity of free NAD remains same in treated and control sample which indicates the inability of compound **19** to inhibit the PARP activity. A known PARP1 inhibitor Olaparib was used as positive control that results increase in fluorescence of free NAD.

**Compound 19 inhibit the ligation activity of cell lysate**

The MDA-MB-231 and 4T1 cells were treated with different concentrations of compound **19** for 48h. Cell lysate were prepared as described previously ^6^ for the ligation assay. Here we used same DNA substrate which was used in FEN1 flap cleavage assay so that we can check the activity of hLigI and FEN1 proteins in compound **19** treated samples. The reactions were performed as described previously ^6^. In the Supplementary Fig. 74 cleaved product remain (25 mer) same. This cleaved product occurs after the cleavage of 25+6 mer oligo by FEN1 protein, since the cleaved product (25 mer) remains same in treated as well as in control samples we can conclude that the compound **19** unable to inhibit the FEN1 protein inside the cells. The cleaved product (25 mer) make a double strand nicked substrate for hLig I protein with 27 mer and 52 mer oligo used in the reaction. hLigI protein gives rise to ligated (52 mer oligo) product after the completion of reaction. In the treated samples, ligated product (52 mer) decreases as compared to untreated samples. These results indicate that the compound **19** maintains its activity inside the cells and inhibits the ligase activity of cell lysate.


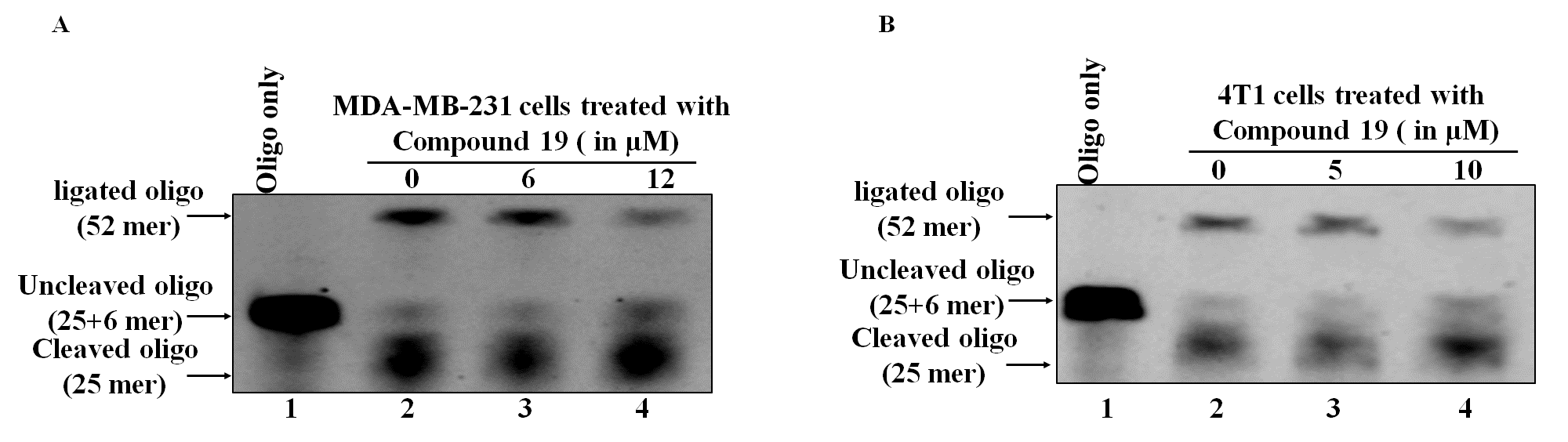


**Supplementary Fig. 74** The gel picture shows that as compared to control cell lysate, in treated samples flap cleaved oligo (25 mer) remain almost constant whereas the amount of ligated oligo (52 mer) decreases. This indicates that compound **19** can inhibit DNA ligase activity in cell free extracts of (A) MDA-MB-231 and (B) 4T1 cells.

***In-silico* interaction study between compound 19 and hLigI**

To elucidate the binding mode of compound **19**, we have thoroughly analysed the docked complex of compound **19** with hLigI to identify interactions portrayed by the ligand with the active site residues. It was found that the compound was docked very well inside the DNA binding cavity identified by Chen *et al* ^7^. Supplementary Fig. 75 shows docked conformation the compound in hLigI. As it can be seen from the Supplementary Fig., the compound **19** occupies position in the DNA binding domain of hLigI. The interacting residues with ligands are accommodated such that they don’t allow the DNA to interact with the protein, after the binding of ligand to protein. Key interactions of compound **19** can also be seen in Supplementary Fig. 75.


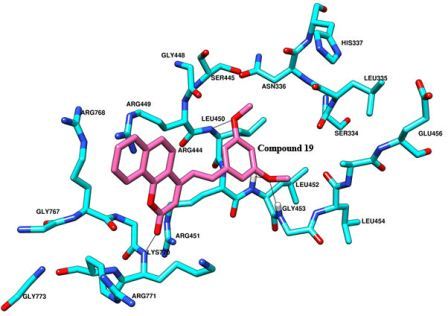


**Supplementary Fig. 75** Docking of Compound **19** (Pink sticks) with hLigI in the DNA binding site as predicted in the crystal structure (Cyan color). Hydrogen bonds are represented as black solid lines.

The backbone nitrogen atom of Lys770 residue of hLigI is forming a hydrogen bond with carbonyl oxygen attached with the pyran ring present in the compound. Another important residue Gly453 is interacting with a hydrogen bond in compound **19**. The backbone nitrogen of Leu450 is also forming a hydrogen bond with methoxy oxygen attached with one of the benzene ring of compound **19**. In compound **19** Leu452 is forming a hydrogen bond with methoxy oxygen attached with one of the benzene ring. All these interactions are known to involve in DNA binding by forming hydrogen bonds in the crystal structure of DNA. Since, it is evident from the above description that the interactions which are playing important role in ligand binding are also involved in DNA binding of the protein, therefore these interactions might contribute primarily in inhibition of ligation reaction.

**Tables.**

**Supplementary Table 1. Strucure and antiproliferative activity of Benzocoumarin-stelbene hybrids in MDA-MB-231 cells at 10μM concentration.**

| **Compound No** | **Structure** | **Antiproliferative activity at 10μM in MDA-MB-231** |
| --- | --- | --- |
| 10 |  | NA |
| 11 |  | 1.46 |
| 12 |  | 4.62 |
| 13 |  | NA |
| 14 |  | 12.43 |
| 15 |  | NA |
| 16 |  | 56.48 |
| 17 |  | NA |
| 18 |  | 8.2 |
| **19** |  | **51.35** |
| 20 |  | 7.53 |
| 21 |  | 0.16 |
| 22 |  | NA |
| 23 |  | 6.04 |
| 24 |  | 20.01 |
| 25 |  | 27.87 |
| 26 |  | 50.03 |
| 27 |  | 8.81 |
| 28 |  | NA |
| 29 |  | 10.4 |
| 30 |  | 17.9 |
| 31 |  | 1.7 |
| 32 |  | 18.2 |
| 33 |  | 6.12 |
| 34 |  | 0.54 |
| 35 |  | 12.8 |
| 36 |  | NA |
| 37 |  | 11.6 |
| 38 |  | 22.01 |
| 39 |  | 10.09 |

NA- Not active

**Supplementary Table 2. Percent inhibition of cell viability shown by compounds 16, 19 and 26**

**in different cancer cell lines at 10 µM concentration.**

|  | **% Inhibition of cell viability at 10 µM in different cancer cell lines** | | | | | | | | | |
| --- | --- | --- | --- | --- | --- | --- | --- | --- | --- | --- |
| Comp | PANC-1  (Pancreas) | DLD1  (Colon) | MDA-MB-231  (Breast) | PLC/PRF/5  (Liver) | A549  (Lung) | SK-OV-3 (Ovary) | A172  (Brain) | 4T1  (Mouse Breast Cancer) | HEK-293  (Non-cancerous cell) | MCF-10A (Normal breast epithelial cells) |
| 16 | NA | NA | **56.48** | NA | NA | NA | NA | 33.89 | ND | ND |
| **19** | **50.32** | **42.64** | **51.35** | **NA** | **NA** | **NA** | **43.71** | **53.18** | **37.83** | **50.83** |
| 26 | NA | NA | **50.93** | ND | NA | ND | ND | 36.12 | ND | ND |

NA= Not active (% Inhibition <30%), ND = Not done

**Supplementary Table 3. Docking energy of compound 19 with different DNA replication and repair proteins. The docking energy of compound 19 with hLigI was the most energetically favorable.**

| **Proteins** | **Docking energy (kJ/mol)** |
| --- | --- |
| Human DNA ligase I (hLigI) | -21.85 |
| Poly [ADP-ribose] polymerase 1 (PARP1) | -18.23 |
| Flap endonuclease 1 (FEN1) | -14.49 |
| Topoisomerase I (TOPO1) | -13.27 |
| Proliferating cell nuclear antigens (PCNA) | -10.67 |

**Supplementary Table 4. Compound 19 followed Lipiski’s rule of five. The values were calculated by online free server developed by Jayaram et al ^8^ (http://www.scfbio-iitd.res.in/software/drugdesign/lipinski.jsp#anchortag)**

| **Parameters** | **Values** |
| --- | --- |
| Molecular mass | 358 |
| High lipophilicity (expressed as LogP) | 4.87 |
| Hydrogen bond donors | 0 |
| Hydrogen bond acceptors | 4 |
| Molar refractivity | 106.13 |

**Refrences**

1 Dong, Y. *et al.* Antitumor agents. 272. Structure-activity relationships and in vivo selective anti-breast cancer activity of novel neo-tanshinlactone analogues. *J Med Chem* **53**, 2299-2308, DOI: 10.1021/jm1000858 (2010).

2 Choi, H. Y. & Chi, D. Y. Nonselective bromination-selective debromination strategy: selective bromination of unsymmetrical ketones on singly activated carbon against doubly activated carbon. *Org Lett* **5**, 411-414, DOI: 10.1021/ol0271638 (2003).

3 Olive, P. L. & Banath, J. P. The comet assay: a method to measure DNA damage in individual cells. *Nat Protoc* **1**, 23-29, DOI: 10.1038/nprot.2006.5 (2006).

4 Langelier, M. F., Planck, J. L., Servent, K. M. & Pascal, J. M. Purification of human PARP-1 and PARP-1 domains from Escherichia coli for structural and biochemical analysis. *Methods Mol Biol* **780**, 209-226, DOI: 10.1007/978-1-61779-270-0_13 (2011).

5 Hegde, M. *et al.* Novel PARP inhibitors sensitize human leukemic cells in an endogenous PARP activity dependent manner. *RSC Adv*, 6308-6319, DOI: 10.1039/C5RA19150E (2016).

6 Singh, D. K. *et al.* Identification of a novel human DNA ligase I inhibitor that promotes cellular apoptosis in DLD-1 cells: an in silico and in vitro mechanistic study. *RSC Adv*, 94574-94587, DOI: 10.1039/C6RA22364H (2016).

7 Chen, X. *et al.* Rational design of human DNA ligase inhibitors that target cellular DNA replication and repair. *Cancer Res* **68**, 3169-3177, DOI: 10.1158/0008-5472.CAN-07-6636 (2008).

8 Jayaram, B. *et al.* Sanjeevini: a freely accessible web-server for target directed lead molecule discovery. *BMC Bioinformatics* **13** Suppl 17, S7, DOI: 10.1186/1471-2105-13-S17-S7 (2012).
